# Oxidative Asymmetric Formal Aza-Diels–Alder Reactions of Tetrahydro-β-carboline with Enones in the Synthesis of Indoloquinolizidine-2-ones

**DOI:** 10.3390/molecules23092228

**Published:** 2018-09-01

**Authors:** Xiang Wu, Shi-Bao Zhao, Lang-Lang Zheng, You-Gui Li

**Affiliations:** Anhui Province Key Laboratory of Advanced Catalytic Materials and Reaction Engineering, School of Chemistry and Chemical Engineering, Hefei University of Technology, Hefei 230009, China; zsbhfut@mail.hfut.edu.cn (S.-B.Z.), zhengll@mail.hfut.edu.cn (L.-L.Z.)

**Keywords:** aza-Diels–Alder reaction, indoloquinolizidine-2-ones, enantioselective catalysis, cooperative catalysis, ruthenium

## Abstract

Ru-catalyzed tandem amine oxidative dehydrogenation/formal aza-Diels–Alder reaction for enantio- and diastereoselective synthesis of indoloquinolizidine-2-ones from tetrahydro-β-carbolines and α,β-unsaturated ketones is described. The reaction proceeds via tandem ruthenium-catalyzed amine dehydrogenation using tert-butyl hydroperoxide (TBHP) as the oxidant and a chiral thiourea-catalyzed formal aza-[4 + 2] cycloaddition, providing a step-economical strategy for the synthesis of these valuable heterocyclic products.

## 1. Introduction

**Figure 1:**
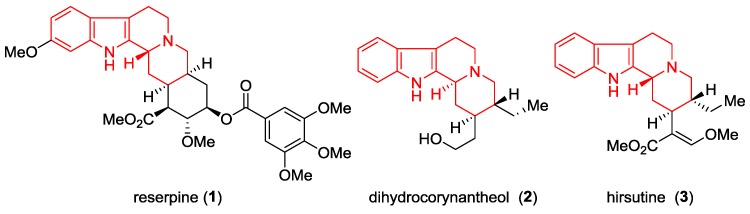
Selected indole alkaloids containing indoloquinolizidine motifs.

**Scheme 1:**
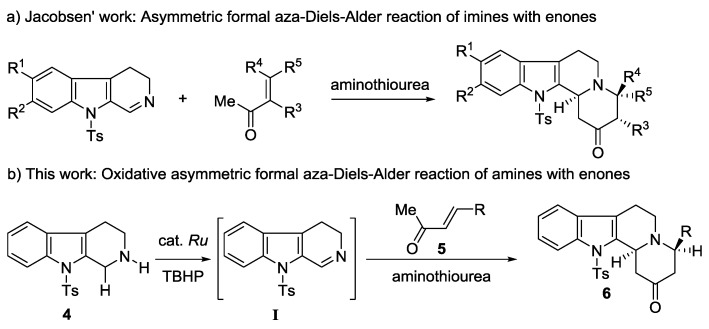
Asymmetric formal aza-Diels–Alder reaction for the synthesis of Indoloquinolizidine-2-ones.

Aza-Diels–Alder reaction is one of the most efficient and direct approaches for the synthesis of six-membered *N*-heterocycles, especially in the syntheses of biologically active natural products and pharmaceutical compounds [1,2,3,4,5,6,7,8,9]. Despite all these advantages, aza-Diels–Alder reaction has been rarely applied in the synthesis of indoloquinolizidine derivatives [10,11,12]. Indoloquinolizidine, a unique nitrogen-containing skeleton, is often embedded in indole alkaloids—such as reserpine **1**, dihydrocorynantheol **2**, and hirsutine **3**—which exhibit a wide range of biological activities (Figure 1) [13,14,15]. In 2006, Itoh and co-workers described a proline-catalyzed asymmetric formal aza-Diels–Alder reaction for the synthesis of *ent*-dihydrocorynantheol **2** [16,17]. In 2013, Jacobsen et al. reported a highly enantio- and diastereoselective synthesis of indololizidine compounds through the formal aza-Diels–Alder reaction of cyclic imines with enones catalyzed by a bifunctional primary aminothiourea (Scheme 1a) [18], which has been approved to be an efficient and practical method by a successful enantioselective total synthesis of reserpine **1** [19]. Recently, the oxidative aza-Diels–Alder reaction, an emerging efficient approach combining two processes of oxidative generation of imines and subsequent cycloaddition, has been applied in synthesis of *N*-heterocycles [20,21,22,23,24,25,26,27,28,29,30,31,32,33,34,35,36]. In this process, C–C and C–N bonds are formed simultaneously via direct oxidative functionalization of C–H [37,38,39] and N–H bonds, and tedious pre-functionalization and intermediate purification are avoided. In continuation of our ongoing studies on the oxidative cycloadditions for the synthesis of structurally complex compounds [24,36], herein we report the Ru-catalyzed tandem amine oxidative dehydrogenation/formal aza-Diel–Alder reaction for enantio- and diastereoselective synthesis of indoloquinolizidine-2-ones **6** from α,β-unsaturated ketones **5**, in which the cyclic imine dienophiles **I** were generated in situ from tetrahydro-β-carbolines **4** by oxidation (Scheme 1b).

## 2. Results and Discussion

At the beginning of this work, we evaluated the model reaction of tetrahydro-β-carboline **4** with enone **5a**, in the presence of acetic acid, using *tert*-butyl hydroperoxide (TBHP) in decane as the oxidant and primary amine-thiourea **T** as the bifunctional catalyst which allows dual activation of the reaction components by the hydrogen bond donor and primary amine (Table 1) [18,19,36]. In the presence of the oxidant alone, the desired formal aza-Diels–Alder product **6** could not be obtained (entry 1). In the presence of Cu salt, the diastereoisomers **6aa** and **7aa** were obtained in 22% yield with poor diastereomeric ratio (dr) and enantioselectivities (entry 2). Ruthenium complexes exhibit cytochrome P450-like activity to catalyze selective oxidative demethylation of tertiary methyl amines in the presence of peroxides or molecular oxygen [40,41,42]. When several r4uthenium catalysts were employed in the reaction system, the desired products could be obtained in moderate yields with moderate dr and ee (entries 3−5). Among these, RuCl_2_(PPh_3_)_3_ gave the best enantioselectivety (69% ee of **6aa** and 66% ee of **7aa**) albeit in lower yield and poorer dr (entry 5). Interestingly, when the amount of acetic acid decreased to 15 mol %, the ee values increased significantly to 87% and 83% respectively and the yield and dr were both improved slightly (entry 6 vs. entry 5). Compared to PhCOOH, *o*-F-PhCOOH or the absence of acid, CH_3_CO_2_H gave the best results in the reaction (entry 6 vs. entries 7–9). Several oxidants were further investigated, among them only TBHP could afford the desired products effectively (entry 6 vs. entries 10–12). Final evaluation of the solvents showed that toluene was the most suitable medium to give the best results (entry 6 vs. entries 13–16). When the reaction solution was diluted to 0.2 M in toluene, the best yield (73%) and enatioselectivities (94% ee of **6aa** and 90% ee of **7aa**) were obtained with moderate diastereomeric ratio (entry 17).

Having established the optimal reaction conditions, the generality of this oxidative formal aza-Diel–Alder reaction for enones was then investigated. A variety of aryl-substituted enones **5b**-**n** reacted smoothly with tetrahydro-β-carboline **4** under the optimized conditions to afford products **6ab**-**an** in moderate to satisfactory yields with good diastereoselectivities and high enantioselectivities (Table 2). Regardless of the position of the methyl group on the phenyl ring, moderate yields and high ee values were obtained (entries 2–4). Electron-withdrawing groups in the different positions of the phenyl ring afforded the corresponding products **6af**-**ai** with good results (entries 5–8). Interestingly, substrate **5j** with five fluorine atoms on the phenyl ring or the substrates **5k** and **5l** with two substituents on the phenyl rings gave the desired products with excellent diastereoselectivities (>10:1) in moderate yields. Moreover, α,β-unsaturated ketones bearing 2-thiophene or 2-furan also performed well and led to the products with good stereochemical outcomes (**6am** and **6an**). Importantly, linear alkyl group subtituted enone **5o** could also undergo the reaction smoothly and afford the product **6ao** with excellent diastereoselectivities (>10:1) and good enantioselectivity, albeit the yield was low relatively, even prolonging reaction time to 96 h. Some other enones such as 5-methyl-3-methylenehexan-2-one and (*E*)-1-phenylpent-1-en-3-one have been employed in the reaction, unfortunately, no desired products were obtained (see Supplementary Materials). The absolute configuration of **6** and **7** was established according to the retention time in HPLC using chiral columns and comparison with the data obtained for the same known products reported by Jacobsen et al. [18].

**Figure 2:**
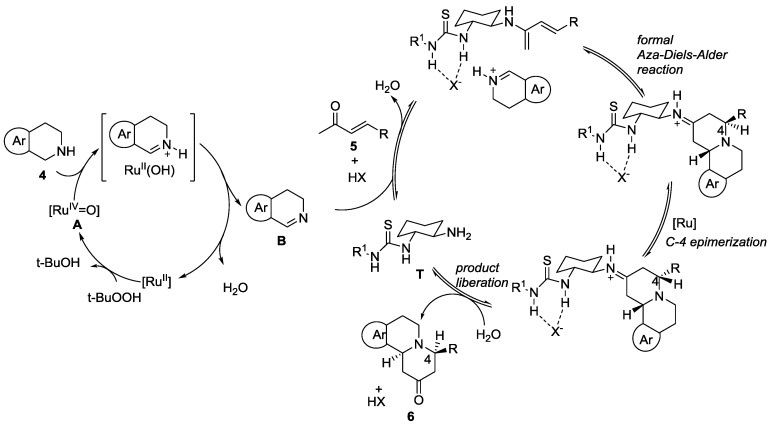
Proposed reaction pathway.

Similar to the results of the 1,2,3,4-tetrahydroisoquinolines [36], under oxidative conditions, tetrahydro-β-carbolines afforded corresponding (4*R*,12*bS*)-**6** as the major stereoisomer having a *cis-*H/H relationship which is different from the outcomes of the formal aza-Diels–Alder reaction of imines with enones [18]. Therefore, the same plausible mechanism can be proposed (Figure 2). Intermediate imine **B** (dihydro-β-carboline) can be formed through the oxidation of **4** by oxoruthenium (IV) intermediate **A** which is generated from the Ru(II) catalyst in the presence of TBHP. Then, imine **B** enters another catalytic cycle and undergoes a formal aza-Diels–Alder reaction with enone, which is similar to the pathway hypothesized by Jacobsen’s group. Finally, the Ru complex present in the reaction mixture promotes epimerization at C-4, leading to the thermodynamic (4*R*,12*bS*)-adducts (**6**) after liberation from the thiourea catalyst **T**. The epimerization can take place by either retro-Mannich/Mannich or amine β-elimination/conjugated addition sequences [18]. 

## 3. Materials and Methods 

NMR spectra were recorded on Aglient-600 MHz (Agilent Technologies, PaloAlto, CA, USA) or Brucker-400 MHz spectrometer (Bruker, Billerica, MA, USA) using CDCl_3_ as solvent and TMS as internal standard unless otherwise stated. Mass spectra were recorded on a Thermo LTQ Orbitrap XL (ESI+) (Bruker, Billerica, MA, USA). HPLC analysis was performed on Agilent 1200 (UV detection monitored at 210 nm) (Waters, Milford, MA, USA). Chiralpak OD-H, AD-H, and IC-H columns were purchased from Daicel Chemical Industries, LTD. (Shanghai, China) Specific optical rotations ([α]) were measured using a Perkin-Elmer 341 polarimeter (PerkinElmer, Waltham, MA, USA) at 25 °C with a sodium lamp (D line, 589 nm). Column chromatography was performed on silica gel (200–300 mesh) eluting with ethyl acetate and petroleum ether. TLC was performed on glass-backed silica plates. Ketone substrates were prepared according to the literature report [43]. Thiourea **T** was prepared according to the literature report [18]. 9-Tosyl-2,3,4,9-tetrahydro-1*H*-pyrido[3,4-*b*]indole was prepared according to the literature report [44,45]. 

Typical procedure for the ruthenium-catalyzed enantioselective oxidative formal aza-Diels–Alder reactions: Thiourea **T** (14.0 mg, 0.03 mmol, 0.15 equiv.), tris(triphenylphosphine)ruthenium (II) dichloride (3.8 mg, 0.004 mmol, 0.06 equiv.), 9-tosyl-2,3,4,9-tetrahydro-1*H*-pyrido[3,4-*b*]indole (**4**) (65.2 mg, 0.2 mmol, 1.0 equiv.), (*E*)-4-(4-methoxyphenyl)but-3-en-2-one (**5a**) (52.8 mg, 0.3 mmol, 1.5 equiv.) were loaded into a tube equipped with a stir bar. A stock solution of glacial acetic acid in anhydrous toluene (0.5 M) was added in one portion at room temperature via syringe (60 µL, 0.03 mmol AcOH, 0.15 equiv.). Anhydrous toluene (1 mL) was then added. The reaction mixture was stirred at 0 °C for 10 min, then the solution of tert-butyl hydroperoxide in decane (5.5 M) was added dropwise at 0 °C via syringe (32 µL, 0.2 mmol TBHP, 1 equiv.) over 45 min. The reaction was stirred at 0 °C for 72 h. The crude mixture was concentrated and was purified through column chromatography on silica gel (petroleum ether/EtOAc = 30/1 to 5/1) to afford title compounds **6aa** and **7aa**.

## 4. Conclusions

In conclusion, we have presented the diastereo- and enantioselective oxidative formal aza-Diels–Alder reaction of tetrahydro-β-carboline and α,β-unsaturated ketones under the cooperative catalysis of Ru(II) salt and chiral aminothiourea in the presence of TBHP, yielding functionalized (4*R*,12b*S*)-indoloquinolizidine-2-ones. The practical protocol of asymmetric oxidative formal aza-Diels–Alder enlarges the substrate scope and offers interesting new opportunities to synthesize the natural products and pharmaceutical compounds further.

## Figures and Tables

**Table 1:**
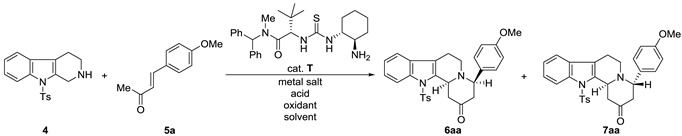
Optimization of the reaction conditions *.

Entry	Metal Salt	Acid (mol %)	Oxidant	Solvent	Yield ** (%)	dr *** (6aa:7aa)	ee **** (%) (6aa/7aa)
1	–	CH_3_COOH (30)	TBHP	toluene	–	–	–
2	Cu(OAc)_2_	CH_3_COOH (30)	TBHP	toluene	22	1.8:1	15/14
3	RuCl_3_·xH_2_O	CH_3_COOH (30)	TBHP	toluene	44	3.4:1	27/35
4	[RuCl_2_(*p*-cymene)]_2_	CH_3_COOH (30)	TBHP	toluene	50	1.8:1	60/59
5	RuCl_2_(PPh_3_)_3_	CH_3_COOH (30)	TBHP	toluene	37	1.3:1	69/66
6	RuCl_2_(PPh_3_)_3_	CH_3_COOH (15)	TBHP	toluene	40	2:1	87/83
7	RuCl_2_(PPh_3_)_3_	PhCOOH (15)	TBHP	toluene	32	1:1	79/76
8	RuCl_2_(PPh_3_)_3_	*o*-F-PhCOOH (15)	TBHP	toluene	30	2.8:1	71/71
9	RuCl_2_(PPh_3_)_3_	–	TBHP	toluene	24	2.2:1	51/51
10	RuCl_2_(PPh_3_)_3_	CH_3_COOH (15)	PhI=O	toluene	–	–	–
11	RuCl_2_(PPh_3_)_3_	CH_3_COOH (15)	DDQ	toluene	–	–	–
12	RuCl_2_(PPh_3_)_3_	CH_3_COOH (15)	H_2_O_2_	toluene	trace	–	–
13	RuCl_2_(PPh_3_)_3_	CH_3_COOH (15)	TBHP	THF	17	1.8:1	89/89
14	RuCl_2_(PPh_3_)_3_	CH_3_COOH (15)	TBHP	CHCl_3_	7	6:1	64/70
15	RuCl_2_(PPh_3_)_3_	CH_3_COOH (15)	TBHP	CH_3_CN	11	10:1	75/72
16	RuCl_2_(PPh_3_)_3_	CH_3_COOH (15)	TBHP	CH_2_Cl_2_	21	8:1	70/70
17 ^	RuCl_2_(PPh_3_)_3_	CH_3_COOH (15)	TBHP	toluene	73	1.8:1	94/90

* Unless indicated otherwise, the reaction of **4** (0.2 mmol), **5a** (0.3 mmol), thiourea catalyst **T** (0.03 mmol), metal salt (0.004 mmol), acid and oxidant (0.2 mmol) were carried out in 0.25 mL of solvent at 0 °C over 72 h. ** Yields of isolated diastereomeric mixture following flash column chromatography on silica gel. *** Determined by ^1^H-NMR analysis. **** Determined by HPLC analysis. ^ 1 mL of anhydrous toluene was used.

**Table 2:**
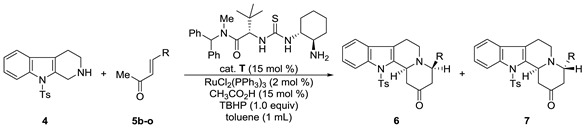
Scope of various α,β-unsaturated ketones. *

Entry	5	R	t (h)	6	Yield ** (%)	dr *** (6:7)	ee **** (%) (6/7)
1	**5b**	C_6_H_5_	48	6ab	31	1:1	96/91
2	**5c**	*o*-MeC_6_H_4_	72	6ac	36	1.8:1	82/95
3	**5d**	*m*-MeC_6_H_4_	72	6ad	41	1:1	88/92
4	**5e**	*p*-MeC_6_H_4_	72	6ae	61	1.5:1	94/96
5	**5f**	*p*-ClC_6_H_4_	72	6af	34	1:1	93/91
6	**5g**	*p*-BrC_6_H_4_	48	6ag	65	1.2:1	85/87
7	**5h**	*p*-CF_3_C_6_H_4_	36	6ah	35	1.6:1	86/93
8	**5i**	*m*-NO_2_C_6_H_4_	72	6ai	37	1:1	94/85
9	**5j**	C_6_F_5_	48	6aj	57	>10:1	86
10	**5k**	2,3-Cl_2_C_6_H_3_	72	6ak	45	>10:1	92
11	**5l**	2,4-(NO_2_)_2_C_6_H_3_	72	6al	45	>10:1	85
12	**5m**	2-thienyl	60	6am	48	1.3:1	89/93
13	**5n**	2-furyl	48	6an	42	1:1	96/95
14	**5o**	CH=CMe_2_	96	6ao	24	>10:1	85

* Reaction conditions: **4** (0.2 mmol), **5** (0.3 mmol), **T** (0.03 mmol), AcOH (0.03 mmol), RuCl_2_(PPh_3_)_3_ (0.004 mmol), and TBHP (0.2 mmol) in toluene (1.0 mL) at 0 °C. ** Yields of isolated diastereomeric mixture following flash column chromatography on silica gel. *** Determined by ^1^H-NMR analysis. **** Determined by HPLC analysis.

## Supplementary Materials

Electronic Supplementary Information (ESI) are available online: General and characterization data, ^1^H- and ^13^C-NMR spectra for all compounds, are available online.
