# Supplementary material for: Oxidative Asymmetric Formal Aza-Diels–Alder Reactions of Tetrahydro-β-carboline with Enones in the Synthesis of Indoloquinolizidine-2-ones
Source: Molecules. 2018 Sep 1;23(9):2228. doi: 10.3390/molecules23092228 (PMC6225203; doi:10.3390/molecules23092228)
Supplement: Supplementary file 1 [file molecules-23-02228-s001.pdf]

Oxidative Asymmetric Formal Aza-Diels−Alder Reactions of Tetrahydro-β-carboline with Enones in the Synthesis of Indoloquinolizidine-2-ones

Xiang Wu,* Shi-Bao Zhao, Lang-Lang Zheng, You-Gui Li*

Anhui Province Key Laboratory of Advanced Catalytic Materials and Reaction Engineering, School of Chemistry and Chemical Engineering, Hefei University of Technology, Hefei 230009, China.
*Correspondence: E-mail: wuxiang@hfut.edu.cn; liyg@hfut.edu.cn

1. General Data S2

2. Typical Procedure for the Ruthenium-Catalyzed Enantioselective Oxidative Formal Aza-Diels−Alder Reactions S2

3. Optimization of the reaction conditions and Scope of α, β-unsaturated ketones S3

4. Characterization Data for the Products S3

5. Reference S15

6. ^{1}H NMR and ^{13}C NMR Spectra for the Products S16

7. HPLC Analysis for the Products S40

1. General Data

NMR spectra were recorded on Aglient-600 MHz or Brucker-400 MHz spectrometer using $CDCl_3$ as solvent and TMS as internal standard unless otherwise stated. Mass spectra were recorded on a Thermo LTQ Orbitrap XL (ESI+). HPLC analysis was performed on Agilent 1200 (UV detection monitored at 210 nm). Chiralpak OD-H, AD-H, IC-H columns were purchased from Daicel Chemical Industries, LTD. Specific optical rotations ([α]) were measured using a Perkin-Elmer 341 polarimeter at 25 ℃ with a sodium lamp (D line, 589 nm).Column chromatography was performed on silica gel (200-300 mesh) eluting with ethyl acetate and petroleum ether. TLC was performed on glass-backed silica plates. Ketone substrate were prepared following the literature report[1]. Thiourea **T** was prepared following the literature report[2]. 9-Tosyl-2,3,4,9-tetrahydro-1H-pyrido[3,4-b]indole was prepared following the literature report[3-4]

2. Typical Procedure for the Ruthenium-Catalyzed Enantioselective Oxidative Formal Aza-Diels−Alder Reactions

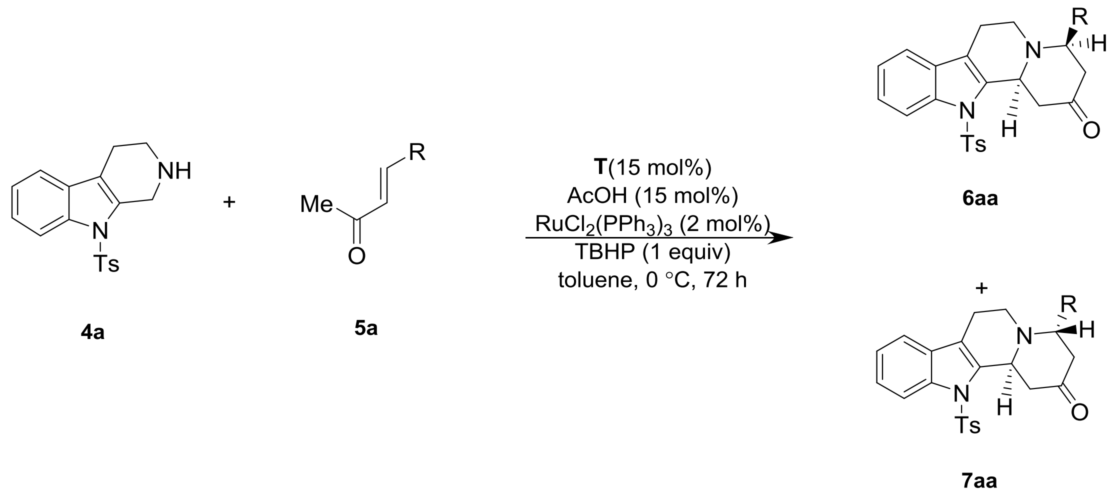

Typical Procedure for the Ruthenium-Catalyzed Enantioselective Oxidative Formal Aza-Diels−Alder Reactions: Thiourea **T** (14.0 mg, 0.03 mmol, 0.15 equiv), tris(triphenylphosphine)ruthenium (II) dichloride (3.8 mg, 0.004 mmol, 0.06 equiv.), 9-tosyl-2,3,4,9-tetrahydro-1*H*-pyrido[3,4-*b*]indole (**4a**) (65.2 mg, 0.2 mmol, 1.0 equiv.), (*E*)-4-(4-methoxyphenyl)but-3-en-2-one (**5a**) (52.8 mg, 0.3 mmol, 1.5 equiv) were loaded into a tube equipped with a stir bar. A stock solution of glacial acetic acid in anhydrous toluene (0.5 M) was added in one portion at room temperature via syringe (60 μL, 0.03 mmol AcOH, 0.15 equiv.). Anhydrous toluene (1 mL) was then added. The reaction mixture was stirred at 0 ℃ for 10 minutes,

then the solution of tert-butyl hydroperoxide in decane (5.5 M) was added dropwise at 0 ℃ via syringe (32 μL, 0.2 mmol TBHP, 1 equiv.) over 45 minutes. The reaction was stirred at 0 ℃ for 72 hours. The crude mixture was concentrated and was purified through column chromatography on silica gel (petroleum ether/EtOAc = 30/1 to 5/1) to afford title compounds **6aa** and **7aa**.

3. Optimization of the reaction conditions and Scope of α, β-unsaturated ketones

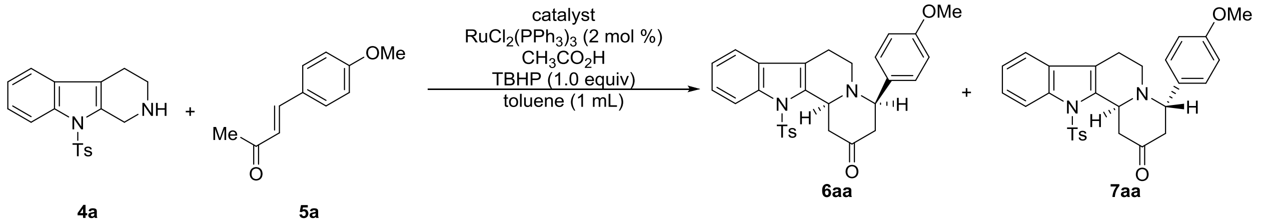

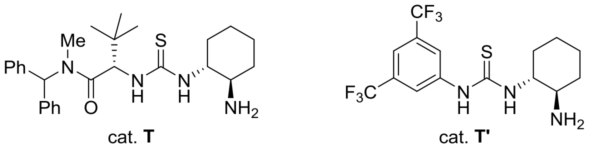

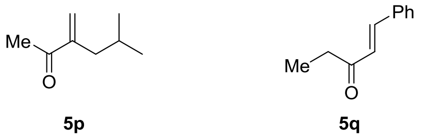

entry	5	catalyst (mol %)	acid (mol %)	yield (%)	dr(6aa:7aa)	ee (%) (6aa/7aa)
1	**5a**	cat. **T** (15)	CH_3COOH (5)	22%	>10:1	81
2	**5a**	cat. **T** (15)	CH_3COOH (0)	24%	2.2:1	51/51
3	**5a**	cat. **T** (5)	CH_3COOH (5)	NP	-	-
4	**5a**	cat. **T′** (15)	CH_3COOH (15)	NP	-	-
5	**5p**	cat. **T** (15)	CH_3COOH (15)	NP	-	-
6	**5q**	cat. **T** (15)	CH_3COOH (15)	NP	-	-

4. Characterization Data for the Products

(4R,12bS)-4-(4-methoxyphenyl)-12-tosyl-1,3,4,6,7,12b-hexahydroindolo[2,3-a]quinolizin-2(12H)-one (6aa: major diastereomer) and (4S,12bS)-4-(4-methoxyphenyl)-12-tosyl-1,3,4,6,7,12b-hexahydroindolo[2,3-a]quinolizin-2(12H)-one (7aa: minor diastereomer): 6aa and **7aa** were obtained as a white solid in 73% yield after flash chromatography.

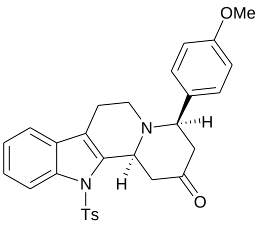

Major diastereomer (6aa): the enantiomeric excess was determined to be 94% by HPLC analysis on Chiralpak AD-H column (30% 2-propanol/n-hexane, 1 mL/min), UV 254 nm, t (major) = 13.58 min, t (minor) = 28.03 min; $[\alpha]_D^{25}$ = +3.1 (c 0. 194, $CHCl_3$); ^{1}H NMR (600 MHz, $CDCl_3$) δ

8.06 (d, *J* = 8.1 Hz, 1H), 7.50 (d, *J* = 7.3 Hz, 2H), 7.32 (d, *J* = 7.3 Hz, 2H), 7.26 (d, *J* = 6.3 Hz, 2H), 7.19 (t, *J* = 7.1 Hz, 1H), 7.09 (d, *J* = 7.6 Hz, 2H), 6.92 (d, *J* = 7.9 Hz, 2H), 4.62 (d, *J* = 10.0 Hz, 1H), 4.17 (s, 1H), 3.81 (s, 3H), 3.48 (d, *J* = 14.7 Hz, 1H), 2.94 (s, 1H), 2.73 (d, *J* = 6.2 Hz, 2H), 2.66 – 2.59 (m, 2H), 2.45 (s, 2H), 2.26 (s, 3H). ^{13}C NMR (101 MHz, $CDCl_3$) δ 207.15, 159.27, 144.75, 137.84, 136.21, 133.97, 130.50, 129.51, 129.25, 128.75, 126.63, 124.94, 124.24, 121.58, 118.62, 116.04, 114.18, 65.00, 58.72, 55.35, 46.21, 45.46, 41.37, 22.35, 21.57. HRMS (ESI) m/z $(M+H)^+$ calculated for $C_{29}H_{29}N_2O_4S$: 501.1843, observed: 501.1845.

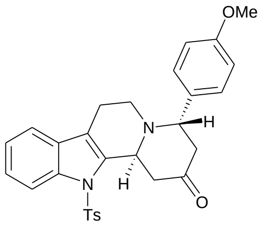

Minor diastereomer (7aa): the enantiomeric excess was determined to be 90% by HPLC analysis on Chiralpak AD-H column (30% 2-propanol/n-hexane, 1 mL/min), UV 254 nm, t (major) = 12.08 min, t (minor) = 10.65 min; $[\alpha]_D^{25}$ = +24.55 (c 0. 128, $CHCl_3$); ^{1}H NMR (600 MHz, $CDCl_3$) δ 8.15 (d, *J* = 8.0 Hz, 1H), 7.39 – 7.20 (m, 5H), 7.09 (d, *J* = 7.6 Hz, 2H), 6.91 (d, *J* = 7.8 Hz, 4H), 4.60 – 4.48 (m, 2H), 3.82 (s, 3H), 3.42 (s, 1H), 3.17 – 3.06 (m, 2H), 2.91 (dt, *J* = 15.5, 10.8 Hz, 3H), 2.77 (d, *J* = 15.2 Hz, 1H), 2.65 – 2.57 (m, 1H), 2.24 (s, 3H). ^{13}C NMR (101 MHz, $CDCl_3$) δ 208.00, 158.86, 137.02, 136.03, 134.50, 133.08, 130.31, 129.58, 129.21, 126.25, 124.76, 124.03, 118.52, 115.56, 113.74, 63.91, 55.36, 50.80, 44.73, 43.89, 39.89, 22.41, 21.57. HRMS (ESI) m/z $(M+H)^+$ calculated for $C_{29}H_{29}N_2O_4S$: 501.1843, observed: 501.1846.

(4R,12bS)-4-phenyl-12-tosyl-1,3,4,6,7,12b-hexahydroindolo[2,3-a]quinolizin-2(12H)-one (6ab: major diastereomer) and (4S,12bS)-4-phenyl-12-tosyl-1,3,4,6,7,12b-hexahydroindolo[2,3-a]quinolizin-2(12H)-one (7ab: minor diastereomer): 6ab and 7ab were obtained as a white solid in 31% yield after flash chromatography.

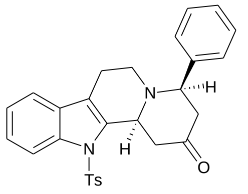

Major diastereomer (6ab): the enantiomeric excess was determined to be 96% by HPLC analysis on Chiralpak AD-H column (30% 2-propanol/n-hexane, 1 mL/min), UV 254 nm, t (major) = 12.15 min, t (minor) = 22.72 min; $[\alpha]_D^{25}$ = +43.0 (c 0.068, $CHCl_3$); ^{1}H NMR (600 MHz, $CDCl_3$) δ 8.07 (d, *J* = 8.0 Hz, 1H), 7.50 (d, *J* = 7.7 Hz, 2H), 7.45 – 7.35 (m, 4H), 7.33 (d, *J* = 6.2 Hz, 1H), 7.30 – 7.24 (m, 2H), 7.20 (t, *J* = 7.3 Hz, 1H), 7.09 (d, *J* = 7.8 Hz, 2H), 4.63 (d, *J* = 10.7 Hz, 1H), 4.20 (d, *J* = 6.0 Hz, 1H), 3.52 (d, *J* = 14.8 Hz, 1H), 3.01 – 2.92 (m, 1H), 2.76 (dt, *J* = 25.4, 12.9 Hz, 2H), 2.69 – 2.59 (m, 2H), 2.47 (d, *J* = 16.3 Hz, 2H), 2.27 (s, 3H). ^{13}C NMR (101 MHz, $CDCl_3$) δ 207.22, 144.77, 141.53,

137.95, 136.38, 133.88, 130.61, 129.49, 128.91, 127.92, 127.52, 126.63, 124.96, 124.31, 121.98, 118.64, 116.14, 65.70, 58.85, 46.56, 45.83, 42.14, 22.53, 21.58. HRMS (ESI) m/z (M+H)$^+$ calculated for $C_{28}H_{27}N_2O_3S$: 471.1737, observed: 471.1740.

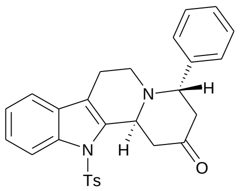

Minor diastereomer (7ab): the enantiomeric excess was determined to be 91% by HPLC analysis on Chiralpak AD-H column (30% 2-propanol/n-hexane, 1 mL/min), UV 254 nm, t (major) = 10.20 min, t (minor) = 8.71 min; $[\alpha]_D^{25}$ = +337.0 (c 0.068, $CHCl_3$); ^{1}H NMR (600 MHz, $CDCl_3$) δ 8.16 (d, *J* = 8.2 Hz, 1H), 7.46 (d, *J* = 7.3 Hz, 2H), 7.41 (t, *J* = 7.3 Hz, 2H), 7.34 (m, 2H), 7.29 (t, *J* = 7.6 Hz, 1H), 7.23 (t, *J* = 7.4 Hz, 1H), 7.06 (d, *J* = 7.9 Hz, 2H), 6.91 (d, *J* = 7.9 Hz, 2H), 4.62 (d, *J* = 5.2 Hz, 1H), 4.54 (d, *J* = 10.5 Hz, 1H), 3.45 (dd, *J* = 9.7, 6.7 Hz, 1H), 3.13 (dd, *J* = 34.4, 12.3 Hz, 2H), 3.02 – 2.86 (m, 3H), 2.78 (d, *J* = 15.0 Hz, 1H), 2.67 – 2.60 (m, 1H), 2.24 (s, 3H). ^{13}C NMR (101 MHz, $CDCl_3$) δ 207.82, 144.57, 140.98, 137.04, 135.95, 134.43, 129.60, 128.52, 128.06, 127.46, 126.23, 124.78, 124.05, 119.12, 118.53, 118.42, 115.61, 64.42, 50.93, 44.90, 43.94, 39.83, 22.41, 21.57. HRMS (ESI) m/z (M+H)$^+$ calculated for $C_{28}H_{27}N_2O_3S$: 471.1737, observed: 471.1738.

(4R,12bS)-4-o-tolyl-12-tosyl-1,3,4,6,7,12b-hexahydroindolo[2,3-a]quinolizin-2(12H)-one (6ac: major diastereomer) and (4S,12bS)-4-o-tolyl-12-tosyl-1,3,4,6,7,12b-hexahydroindolo[2,3-a]quinolizin-2(12H)-one (7ac: minor diastereomer): 6ac and 7ac were obtained as a white solid in 36% yield after flash chromatography.

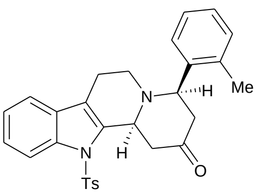

Major diastereomer (6ac): the enantiomeric excess was determined to be 82% by HPLC analysis on Chiralpak AD-H column (30% 2-propanol/n-hexane, 1 mL/min), UV 254 nm, t (major) = 18.42 min, t (minor) = 11.36 min; $[\alpha]_D^{25}$ = +42.0 (c 1.26, $CHCl_3$); ^{1}H NMR (600 MHz, $CDCl_3$) δ 8.10 (d, *J* = 7.7 Hz, 1H), 7.50 (d, *J* = 7.4 Hz, 2H), 7.37 – 7.18 (m, 7H), 7.09 (d, *J* = 7.6 Hz, 2H), 4.73 (s, 1H), 4.43 (d, *J* = 7.4 Hz, 1H), 3.43 (d, *J* = 14.3 Hz, 1H), 2.91 (dd, *J* = 33.4, 20.6 Hz, 2H), 2.70 – 2.57 (m, 3H), 2.52 – 2.37 (m, 5H), 2.28 (s, 3H). ^{13}C NMR (101 MHz, $CDCl_3$) δ 207.58, 144.79, 138.55, 137.79, 137.29, 136.35, 134.06, 131.07, 130.62, 129.51, 127.69, 126.63, 126.34 (d, *J* = 25.1 Hz), 124.95, 124.31, 121.24, 118.63, 116.11, 62.06, 58.44, 45.54, 43.68, 39.93, 22.33, 21.57, 19.18. HRMS (ESI) m/z (M+H)$^+$ calculated for $C_{29}H_{29}N_2O_3S$: 485.1894, observed: 485.1894.

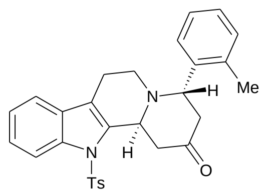

Minor diastereomer (7ac): the enantiomeric excess was determined to be 95% by HPLC analysis on Chiralpak AD-H column (30% 2-propanol/n-hexane, 1 mL/min), UV 254 nm, t (major) = 8.11 min, t (minor) = 5.51 min; $[\alpha]_D^{25}$ = +208.0 (c 0. 19, $CHCl_3$); ^{1}H NMR (600 MHz, $CDCl_3$) δ 8.15 (d, *J* = 8.2 Hz, 1H), 7.33 – 7.26 (m, 5H), 7.23 (m, *J* = 15.9, 8.3 Hz, 2H), 6.88 (d, *J* = 6.7 Hz, 2H), 6.82 (d, *J* = 7.9 Hz, 2H), 4.66 (s, 1H), 4.53 (d, *J* = 9.6 Hz, 1H), 3.47 (td, *J* = 11.0, 3.9 Hz, 1H), 3.20 – 3.10 (m, 2H), 2.94 – 2.82 (m, 3H), 2.74 (dd, *J* = 15.6, 3.4 Hz, 1H), 2.69 – 2.63 (m, 1H), 2.37 (s, 3H), 2.23 (s, 3H). ^{13}C NMR (101 MHz, $CDCl_3$) δ 208.62, 144.38, 137.97, 136.92, 134.47, 130.87, 130.24, 129.94, 129.52, 127.66, 127.14, 126.03, 124.76, 123.97, 118.39, 117.85, 115.62, 62.12, 59.55, 43.89, 42.87, 38.17, 22.14, 21.53, 19.51. HRMS (ESI) m/z $(M+H)^+$ calculated for $C_{29}H_{29}N_2O_3S$: 485.1894, observed: 485.1896.

(4R,12bS)-4-m-tolyl-12-tosyl-1,3,4,6,7,12b-hexahydroindolo[2,3-a]quinolizin-2(12H)-one (6ad: major diastereomer) and (4S,12bS)-4-m-tolyl-12-tosyl-1,3,4,6,7,12b-hexahydroindolo[2,3-a] quinolizin-2(12H)-one (7ad: minor diastereomer): 6ad and 7ad were obtained as a white solid in 41% yield after flash chromatography.

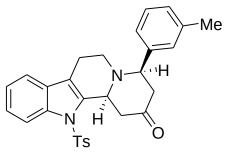

Major diastereomer (6ad): the enantiomeric excess was determined to be 88% by HPLC analysis on Chiralpak AD-H column (30% 2-propanol/n-hexane, 1 mL/min), UV 254 nm, t (major) = 9.60 min, t (minor) = 18.13 min; $[\alpha]_D^{25}$ = +9.4 (c 0.254, $CHCl_3$); ^{1}H NMR (600 MHz, $CDCl_3$) δ 8.07 (d, *J* = 8.1 Hz, 1H), 7.50 (d, *J* = 7.7 Hz, 2H), 7.26 (d, *J* = 8.6 Hz, 3H), 7.23 – 7.17 (m, 3H), 7.13 (d, *J* = 6.9 Hz, 1H), 7.09 (d, *J* = 7.7 Hz, 2H), 4.59 (d, *J* = 10.8 Hz, 1H), 4.17 – 4.10 (m, 1H), 3.51 (d, *J* = 14.8 Hz, 1H), 2.97 (d, *J* = 5.4 Hz, 1H), 2.75 (d, *J* = 6.3 Hz, 2H), 2.68 – 2.60 (m, 2H), 2.47 (d, *J* = 17.7 Hz, 2H), 2.38 (s, 3H), 2.27 (s, 3H). ^{13}C NMR (101 MHz, $CDCl_3$) δ 207.29, 144.73, 141.50, 138.57, 137.99, 136.42, 133.90, 130.64, 129.46, 128.70 (d, *J* = 10.9 Hz), 128.22, 126.65, 124.93, 124.58, 124.29, 122.06, 118.60, 116.17, 65.82, 58.94, 46.63, 46.03, 42.31, 22.56, 21.56. HRMS (ESI) m/z $(M+H)^+$ calculated for $C_{29}H_{29}N_2O_3S$: 485.1894, observed: 485.1893.

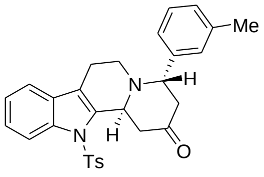

Minor diastereomer (7ad): the enantiomeric excess was determined to be 92% by HPLC analysis on Chiralpak AD-H column (30% 2-propanol/n-hexane, 1 mL/min), UV 254 nm, t (major) = 7.66 min, t

(minor) = 8.95 min; $[\alpha]_D^{25}$ = +201 (c 0.158, $CHCl_3$); ^{1}H NMR (600 MHz, $CDCl_3$) δ 8.16 (d, *J* = 8.0 Hz, 1H), 7.39 – 7.21 (m, 6H), 7.16 (d, *J* = 6.4 Hz, 1H), 7.09 (d, *J* = 7.4 Hz, 2H), 6.91 (d, *J* = 7.4 Hz, 2H), 4.57 (d, *J* = 6.4 Hz, 2H), 3.43 (s, 1H), 3.18 (d, *J* = 15.0 Hz, 1H), 3.10 (s, 1H), 3.02 – 2.86 (m, 3H), 2.78 (d, *J* = 15.2 Hz, 1H), 2.64 (dd, *J* = 25.0, 12.7 Hz, 1H), 2.39 (s, 3H), 2.24 (s, 3H). ^{13}C NMR (101 MHz, $CDCl_3$) δ 206.82, 143.43, 139.88, 137.02, 135.95, 134.97, 133.44, 129.24, 128.49, 127.76, 127.28, 127.10, 125.15, 123.92, 123.67, 122.94, 117.44 (d, *J* = 5.4 Hz), 114.49, 63.28, 49.76, 43.86, 42.85, 38.84, 21.32, 20.61, 20.47. HRMS (ESI) m/z $(M+H)^+$ calculated for $C_{29}H_{28}N_2O_3S$: 485.1894, observed: 485.1896.

(4R,12bS)-4-p-tolyl-12-tosyl-1,3,4,6,7,12b-hexahydroindolo[2,3-a]quinolizin-2(12H)-one (6ae: major diastereomer) and (4S,12bS)-4-p-tolyl-12-tosyl-1,3,4,6,7,12b-hexahydroindolo[2,3-a] quinolizin-2(12H)-one (7ae: minor diastereomer): 6aeand 7ae were obtained as a white so lid in 61% yield after flash chromatography.

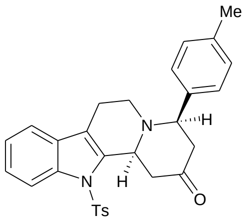

Major diastereomer (6ae): the enantiomeric excess was determined to be 94% by HPLC analysis on Chiralpak AD-H column (30% 2-propanol/n-hexane, 1 mL/min), UV 254 nm, t (major) = 10.46 min, t (minor) = 19.57 min; $[\alpha]_D^{25}$ = +79.0 (c 0.124, $CHCl_3$); ^{1}H NMR (600 MHz, $CDCl_3$) δ 8.07 (d, *J* = 8.1 Hz, 1H), 7.50 (d, *J* = 8.0 Hz, 2H), 7.28 (m, *J* = 21.3, 7.7 Hz, 4H), 7.20 (d, *J* = 7.1 Hz, 3H), 7.09 (d, *J* = 7.9 Hz, 2H), 4.61 (d, *J* = 10.9 Hz, 1H), 4.20 – 4.14 (m, 1H), 3.50 (d, *J* = 14.8 Hz, 1H), 2.98 – 2.91 (m, 1H), 2.75 (d, *J* = 5.5 Hz, 2H), 2.68 – 2.59 (m, 2H), 2.46 (d, *J* = 15.8 Hz, 2H), 2.37 (s, 3H), 2.27 (s, 3H). ^{13}C NMR (101 MHz, $CDCl_3$) δ 207.36, 144.75, 138.50, 137.95, 137.61, 136.46, 133.92, 130.64, 129.52 (d, *J* = 7.6 Hz), 127.43, 126.62, 124.92, 124.30, 121.98, 118.63, 116.14, 65.48, 58.90, 46.54, 45.91, 41.98, 22.55, 21.57, 21.18. HRMS (ESI) m/z $(M+H)^+$ calculated for $C_{29}H_{29}N_2O_3S$: 485.1894, observed: 485.1897.

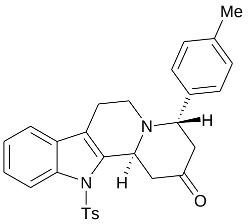

Minor diastereomer (7ae): the enantiomeric excess was determined to be 96% by HPLC analysis on Chiralpak OD-H column (15% 2-propanol/n-hexane, 1 mL/min), UV 254 nm, t (major) = 28.19 min, t (minor) = 24.56 min; $[\alpha]_D^{25}$ = +68.3 (c 0.46, $CHCl_3$); ^{1}H NMR (600 MHz, $CDCl_3$) δ 8.13 (d, *J* = 8.2 Hz, 1H), 7.33 – 7.27 (m, 3H), 7.26 – 7.14 (m, 4H), 7.08 (d, *J* = 7.5 Hz, 2H), 6.90 (d, *J* = 7.8 Hz, 2H), 4.56 (d, *J* = 17.9 Hz, 2H), 3.43 (s, 1H), 3.19 – 3.04 (m, 2H), 2.92 (dt, *J* = 22.8, 11.0 Hz, 3H), 2.76 (d, *J* = 15.2 Hz, 1H), 2.61 (s, 1H), 2.38 (s, 3H), 2.25 (s, 3H). ^{13}C NMR (101 MHz,

CDCl3) δ 207.88, 144.48, 137.86, 137.06, 135.98, 134.51, 130.28, 129.50, 129.17, 127.97, 126.31, 124.76, 123.99, 118.52, 118.43, 115.64, 64.17, 50.83, 43.91, 39.98, 22.37, 21.59, 21.17. HRMS (ESI) m/z $(M+H)^+$ calculated for $C_{29}H_{29}N_2O_3S$: 485.1894, observed: 485.1898.

(4R,12bS)-4-(4-chlorophenyl)-12-tosyl-1,3,4,6,7,12b-hexahydroindolo[2,3-a]quinolizin-2(12H)-one (6af: major diastereomer) and (4S,12bS)-4-(4-chlorophenyl)-12-tosyl-1,3,4,6,7,12b-hexa hydroindolo[2,3-a]quinolizin-2(12H)-one (7af: minor diastereomer): 6af and 7af were obta ined as a white solid in 34% yield after flash chromatography.

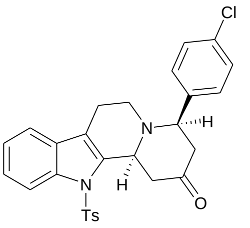

Major diastereomer (6af): the enantiomeric excess was determined to be 93% by HPLC analysis on Chiralpak AD-H column (30% 2-propanol/n-hexane, 1 mL/min), UV 254 nm, t (major) = 14.60 min, t (minor) = 19.85 min; $[\alpha]_D^{25}$ = +68.0 (c 0. 114, $CHCl_3$); ^1H NMR (600 MHz, $CDCl_3$) δ 8.06 (d, *J* = 8.1 Hz, 1H), 7.49 (d, *J* = 7.4 Hz, 2H), 7.35 (s, 4H), 7.26 (d, *J* = 6.0 Hz, 2H), 7.19 (t, *J* = 7.3 Hz, 1H), 7.09 (d, *J* = 7.7 Hz, 2H), 4.64 (d, *J* = 9.9 Hz, 1H), 4.19 (d, *J* = 6.6 Hz, 1H), 3.50 (d, *J* = 14.8 Hz, 1H), 2.92 (s, 1H), 2.77 (d, *J* = 14.3 Hz, 1H), 2.70 – 2.58 (m, 3H), 2.53 – 2.40 (m, 2H), 2.27 (s, 3H). ^{13}C NMR (101 MHz, $CDCl_3$) δ 206.75, 144.82, 140.16, 137.90, 136.20, 133.88, 133.59, 130.51, 129.52, 129.09, 128.84, 126.61, 125.02, 124.34, 121.79, 118.66, 116.10, 64.89, 58.60, 46.49, 45.50, 42.09, 22.49, 21.58. HRMS (ESI) m/z $(M+H)^+$ calculated for $C_{28}H_{26}ClN_2O_3S$: 505.1347, observed: 505.1348.

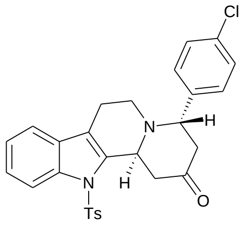

Minor diastereomer (7af): the enantiomeric excess was determined to be 91% by HPLC analysis on Chiralpak AD-H column (30% 2-propanol/n-hexane, 1 mL/min), UV 254 nm, t (major) = 9.75 min, t (minor) = 8.93 min; $[\alpha]_D^{25}$ = +41.0 (c 0.196, $CHCl_3$); ^1H NMR (600 MHz, $CDCl_3$) δ 8.17 (d, *J* = 8.0 Hz, 1H), 7.31 (m, *J* = 34.2, 24.9, 7.0 Hz, 7H), 7.07 (d, *J* = 7.5 Hz, 2H), 6.95 (d, *J* = 7.5 Hz, 2H), 4.57 (s, 1H), 4.39 (d, *J* = 10.7 Hz, 1H), 3.45 (d, *J* = 8.5 Hz, 1H), 3.14 (d, *J* = 16.3 Hz, 2H), 2.99 – 2.86 (m, 3H), 2.78 (d, *J* = 14.9 Hz, 1H), 2.68 – 2.59 (m, 1H), 2.28 (s, 3H). ^{13}C NMR (101 MHz, $CDCl_3$) δ 206.33, 143.68, 138.57, 135.99, 134.49, 133.64, 132.17, 129.00, 128.57, 128.40, 127.51, 125.03, 123.82, 122.95, 117.45, 117.18, 114.44, 62.85, 49.94, 43.70, 42.67, 38.48, 21.30, 20.52. HRMS (ESI) m/z $(M+H)^+$ calculated for $C_{28}H_{26}ClN_2O_3S$: 505.1347, observed: 505.1349.

(4R,12bS)-4-(4-bromophenyl)-12-tosyl-1,3,4,6,7,12b-hexahydroindolo[2,3-a]quinolizin-2(12H)-one(6ag: major diastereomer) and (4S,12bS)-4-(4-bromophenyl)-12-tosyl-1,3,4,6,7,12b-hexa hydroindolo[2,3-a]quinolizin-2(12H)-one (7ag: minor diastereomer): 6ag and 7ag were ob tained as a white solid in 65% yield after flash chromatography.

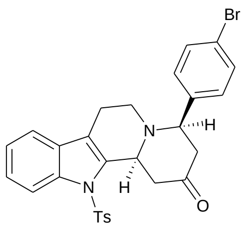

Major diastereomer (6ag): the enantiomeric excess was determined to be 85% by HPLC analysis on Chiralpak AD-H column (30 % 2-propanol/n-hexane, 1 mL/min), UV 254 nm, t (major) = 15.14 min, t (minor) = 22.48 min; $[\alpha]_D^{25}$ = +169.0 (c 0.206, $CHCl_3$); ^1H NMR (400 MHz, $CDCl_3$) δ 8.00 (d, J = 8.1 Hz, 1H), 7.44 (t, J = 8.5 Hz, 4H), 7.28 – 7.18 (m, 5H), 7.17 – 7.11 (m, 1H), 7.03 (d, J = 7.9 Hz, 2H), 4.57 (d, J = 10.3 Hz, 1H), 4.12 (d, J = 7.6 Hz, 1H), 3.44 (d, J = 15.0 Hz, 1H), 2.87 (d, J = 5.3 Hz, 1H), 2.72 (dd, J = 14.5, 3.3 Hz, 1H), 2.58 (dd, J = 26.1, 14.1 Hz, 3H), 2.46 – 2.34 (m, 2H), 2.21 (s, 3H). ^{13}C NMR (101 MHz, $CDCl_3$) δ 205.66, 143.74, 139.61, 136.85, 132.83, 131.00, 129.43, 128.89, 128.45, 128.12, 125.55, 123.96, 123.26, 120.69, 117.58, 115.06, 63.90, 57.54, 45.43, 44.40, 41.08, 21.44, 20.53. HRMS (ESI) m/z $(M+H)^+$ calculated for $C_{28}H_{26}BrN_2O_3S$: 549.0842, observed: 549.0844.

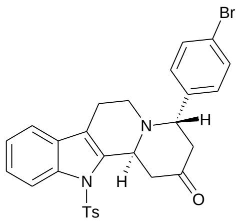

Minor diastereomer (7ag): the enantiomeric excess was determined to be 87% by HPLC analysis on Chiralpak AD-H column (15% 2-propanol/n-hexane, 1 mL/min), UV 254 nm, t (major) = 20.64 min, t (minor) = 16.86 min; $[\alpha]_D^{25}$ = +39.8 (c 0.5, $CHCl_3$); ^1H NMR (600 MHz, $CDCl_3$) δ 8.17 (d, J = 8.3 Hz, 1H), 7.49 (d, J = 8.4 Hz, 2H), 7.34 (d, J = 7.6 Hz, 1H), 7.30 (t, J = 8.9 Hz, 3H), 7.24 (dd, J = 9.1, 5.7 Hz, 1H), 7.07 (d, J = 8.1 Hz, 2H), 6.97 (d, J = 8.1 Hz, 2H), 4.54 (s, 1H), 4.38 (d, J = 10.7 Hz, 1H), 3.45 (td, J = 10.8, 4.2 Hz, 1H), 3.13 (dd, J = 24.8, 13.1 Hz, 2H), 2.97 – 2.87 (m, 3H), 2.78 (d, J = 14.0 Hz, 1H), 2.63 (dd, J = 14.3, 12.1 Hz, 1H), 2.29 (s, 3H). ^{13}C NMR (101 MHz, $CDCl_3$) δ 207.40, 144.78, 140.17, 137.04, 135.51, 134.71, 131.56, 130.06, 129.85, 129.69, 126.09, 124.90, 124.03, 121.42, 118.54, 118.24, 115.50, 63.97, 51.02, 44.76, 43.73, 39.48, 22.37, 21.60. HRMS (ESI) m/z $(M+H)^+$ calculated for $C_{28}H_{26}BrN_2O_3S$: 549.0842, observed: 549.0846.

(4R,12bS)-12-tosyl-4-(4-(trifluoromethyl)phenyl)-1,3,4,6,7,12b-hexahydroindolo[2,3-a]quinoliz in-2(12H)-one (6ah: major diastereomer) and (4S,12bS)-12-tosyl-4-(4-(trifluoromethyl)phe nyl)-1,3,4,6,7,12b-hexahydroindolo[2,3-a]quinolizin-2(12H)-one (7ah: minor diastereomer):6

ah and 7ah were obtained as a white solid in 35% yield after flash chromatography.

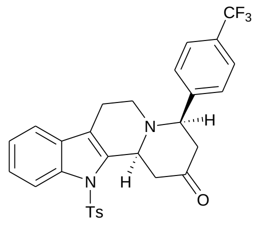

Major diastereomer (6ah): the enantiomeric excess was determined to be 86% by HPLC analysis on Chiralpak AD-H column (30% 2-propanol/n-hexane, 1 mL/min), UV 254 nm, t (major) = 10.71 min, t (minor) =18.96 min; $[\alpha]_D^{25}$ = -317 (c 0. 24, $CHCl_3$); ^1H NMR (600 MHz, $CDCl_3$) δ 8.07 (d, *J* = 8.2 Hz, 1H), 7.65 (d, *J* = 7.7 Hz, 2H), 7.55 (d, *J* = 7.3 Hz, 2H), 7.50 (d, *J* = 7.7 Hz, 2H), 7.29 (d, *J* = 7.4 Hz, 1H), 7.25 (s, 1H), 7.21 (t, *J* = 7.3 Hz, 1H), 7.10 (d, *J* = 7.8 Hz, 2H), 4.67 (d, *J* = 10.5 Hz, 1H), 4.28 (d, *J* = 6.5 Hz, 1H), 3.54 (d, *J* = 15.0 Hz, 1H), 2.95 – 2.90 (m, 1H), 2.84 (dd, *J* = 14.6, 3.1 Hz, 1H), 2.66 (dd, *J* = 28.0, 15.4 Hz, 3H), 2.53 – 2.42 (m, 2H), 2.28 (s, 3H). ^{13}C NMR (101 MHz, $CDCl_3$) δ 206.40, 145.83, 144.81, 137.95, 136.08, 133.90, 130.18 (q, *J* = 32.3 Hz), 129.50, 127.77, 126.62, 125.92 (q, *J* = 3.5 Hz), 125.07, 124.35, 124.04 (q, *J* = 272.7 Hz), 121.8, 118.63, 116.15, 65.13, 58.54, 46.63, 45.47, 42.55, 22.51, 21.57. HRMS (ESI) m/z $(M+H)^+$ calculated for $C_{29}H_{25}F_3N_2O_3S$: 539.1611, observed: 539.1612.

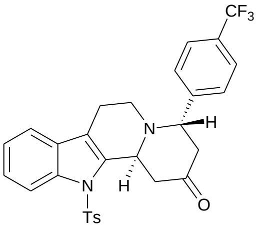

Minor diastereomer (7ah): the enantiomeric excess was determined to be 93% by HPLC analysis on Chiralpak AD-H column (20% 2-propanol/n-hexane, 1 mL/min), UV 254 nm, t (major) =10.45min, t (minor) =8.73 min; $[\alpha]_D^{25}$ = +69.0 (c 0. 064, $CHCl_3$); ^1H NMR (600 MHz, $CDCl_3$) δ 8.16 (d, *J* = 8.2 Hz, 1H), 7.64 (d, *J* = 7.9 Hz, 2H), 7.58 (d, *J* = 7.7 Hz, 2H), 7.35 (d, *J* = 7.4 Hz, 1H), 7.30 (t, *J* = 7.5 Hz, 1H), 7.24 (t, *J* = 7.2 Hz, 1H), 7.05 (d, *J* = 7.7 Hz, 2H), 6.91 (d, *J* = 7.8 Hz, 2H), 4.64 (s, 1H), 4.40 (d, *J* = 10.4 Hz, 1H), 3.48 (d, *J* = 9.8 Hz, 1H), 3.16 (d, *J* = 14.5 Hz, 2H), 3.03 – 2.90 (m, 3H), 2.80 (d, *J* = 15.3 Hz, 1H), 2.71 – 2.62 (m, 1H), 2.25 (s, 3H). ^{13}C NMR (101 MHz, $CDCl_3$) δ 207.12, 145.19, 144.84, 137.05, 135.35, 134.66, 130.06, 129.63 (q, *J* = 32.3 Hz), 129.59, 128.44, 126.02, 125.38 (q, *J* = 3.6 Hz), 124.96, 124.19 (q, *J* = 272.1 Hz), 124.09, 118.57, 118.36, 115.51, 64.21, 51.31, 44.88, 43.79, 39.52, 22.35, 21.49. HRMS (ESI) m/z $(M+H)^+$ calculated for $C_{29}H_{26}F_3N_2O_3S$: 539.1611, observed: 539.1615.

(4R,12bS)-4-(3-nitrophenyl)-12-tosyl-1,3,4,6,7,12b-hexahydroindolo[2,3-a]quinolizin-2(12H)-one (6ai: major diastereomer) and (4S,12bS)-4-(3-nitrophenyl)-12-tosyl-1,3,4,6,7,12b-hexahydroindolo[2,3-a]quinolizin-2(12H)-one (7ai: minor diastereomer): 6ai and 7ai were obtained as a white solid in 37% yield after flash chromatography.

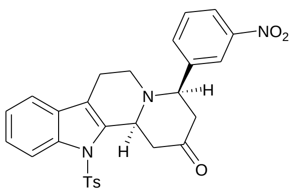

Major diastereomer (6ai): the enantiomeric excess was determined to be 94% by HPLC analysis on Chiralpak AD-H column (30% 2-propanol/n-hexane, 1 mL/min), UV 254 nm, t (major) =21.61 min, t (minor) =29.93 min; $[\alpha]_D^{25}$ = +28.0 (c 0.178, $CHCl_3$); ^{1}H NMR (600 MHz, $CDCl_3$) δ 8.30 (s, 1H), 8.18 (d, *J* = 7.8 Hz, 1H), 8.06 (d, *J* = 8.2 Hz, 1H), 7.77 (d, *J* = 7.2 Hz, 1H), 7.58 (t, *J* = 7.8 Hz, 1H), 7.51 (d, *J* = 7.7 Hz, 2H), 7.31 – 7.24 (m, 2H), 7.20 (t, *J* = 7.2 Hz, 1H), 7.11 (d, *J* = 7.7 Hz, 2H), 4.74 (d, *J* = 10.7 Hz, 1H), 4.41 – 4.34 (m, 1H), 3.53 (d, *J* = 15.2 Hz, 1H), 2.90 (t, *J* = 12.0 Hz, 2H), 2.68 (ddd, *J* = 14.2, 12.7, 7.7 Hz, 3H), 2.55 – 2.45 (m, 2H), 2.28 (s, 3H). ^{13}C NMR (101 MHz, $CDCl_3$) δ 206.02, 148.73, 144.90, 143.91, 137.81, 135.90, 133.93, 133.47, 130.37, 130.04, 129.58, 126.57, 125.10, 124.35, 123.03, 122.48, 121.41, 118.67, 116.04, 64.56, 58.24, 46.33, 44.78, 42.06, 22.45, 21.58. HRMS (ESI) m/z $(M+H)^+$ calculated for $C_{28}H_{26}N_3O_5S$: 516.1588, observed: 516.1589.

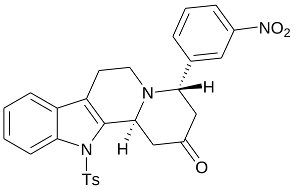

Minor diastereomer (7ai): the enantiomeric excess was determined to be 85% by HPLC analysis on Chiralpak AD-H column (30% 2-propanol/n-hexane, 1 mL/min), UV 254 nm, t (major) =14.74 min, t (minor) = 10.74 min; $[\alpha]_D^{25}$ = +317.0 (c 0.1, $CHCl_3$); ^{1}H NMR (600 MHz, $CDCl_3$) δ 8.44 (s, 1H), 8.17 (d, *J* = 8.0 Hz, 1H), 8.11 (d, *J* = 8.2 Hz, 1H), 7.76 (d, *J* = 7.7 Hz, 1H), 7.57 (t, *J* = 8.0 Hz, 1H), 7.36 (d, *J* = 7.6 Hz, 1H), 7.29 (t, *J* = 7.6 Hz, 1H), 7.24 (m, *J* = 9.7, 4.7 Hz, 1H), 7.08 (d, *J* = 8.2 Hz, 2H), 6.92 (d, *J* = 8.1 Hz, 2H), 4.67 (s, 1H), 4.47 (d, *J* = 10.2 Hz, 1H), 3.51 (td, *J* = 11.0, 4.0 Hz, 1H), 3.20 (dd, *J* = 11.1, 6.4 Hz, 1H), 3.13 – 3.09 (m, 1H), 3.06 – 2.97 (m, 3H), 2.82 (dd, *J* = 15.7, 3.2 Hz, 1H), 2.67 (dd, *J* = 14.8, 11.6 Hz, 1H), 2.24 (s, 3H). ^{13}C NMR (101 MHz, $CDCl_3$) δ 206.43, 148.82, 145.05, 143.35, 137.09, 135.24, 134.44, 133.20, 130.03, 129.71, 129.60 125.93, 125.02, 124.12, 123.33, 122.41, 118.60, 118.43, 115.54, 64.10, 51.68, 44.86, 43.71, 39.42, 22.31, 21.47. HRMS (ESI) m/z $(M+H)^+$ calculated for $C_{28}H_{26}N_3O_5S$: 516.1588, observed: 516.1588.

(4R,12bS)-4-(perfluorophenyl)-12-tosyl-1,3,4,6,7,12b-hexahydroindolo[2,3-a]quinolizin-2(12H)-one (6aj)

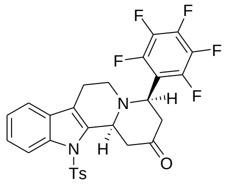

6aj was obtained as a white solid in 57% yield after flash chromatography and the enantiomeric excess was determined to be 86% by HPLC analysis on

Chiralpak AD-H column (30% 2-propanol/n-hexane, 1 mL/min), UV 254 nm, t (major) = 9.16 min, t (minor) = 5.98 min; $[\alpha]_D^{25}$ = +61.0 (c 0.114, $CHCl_3$); ^1H NMR (600 MHz, $CDCl_3$) δ 8.10 (d, *J* = 8.2 Hz, 1H), 7.36 (d, *J* = 7.8 Hz, 2H), 7.30 (m, *J* = 14.6, 7.6 Hz, 2H), 7.23 (m, *J* = 13.7, 6.3 Hz, 1H), 7.03 (d, *J* = 7.7 Hz, 2H), 4.82 (d, *J* = 5.8 Hz, 2H), 3.44 (d, *J* = 17.3 Hz, 1H), 3.23 (d, *J* = 6.3 Hz, 1H), 3.01 (dd, *J* = 16.4, 5.6 Hz, 1H), 2.82 (dd, *J* = 16.4, 7.0 Hz, 1H), 2.75 (d, *J* = 12.3 Hz, 2H), 2.68 (t, *J* = 11.4 Hz, 1H), 2.59 (dd, *J* = 17.3, 11.5 Hz, 1H), 2.26 (s, 3H). ^{13}C NMR (101 MHz, $CDCl_3$) δ 205.56, 145.25 (m), 144.96, 140.66 (m), 137.66 (m), 137.64, 135.16, 134.55, 130.02, 129.58, 126.15, 125.08, 124.24, 119.60, 118.63, 115.77, 114.34 (t, *J* = 16.8 Hz), 55.21, 51.22, 46.06, 44.79, 41.32, 22.20, 21.48. HRMS (ESI) m/z $(M+H)^+$ calculated for $C_{28}H_{22}F_5N_2O_3S$: 561.1266, observed: 561.1268.

(4R,12bS)-4-(2,3-dichlorophenyl)-12-tosyl-1,3,4,6,7,12b-hexahydroindolo[2,3-a]quinolizin-2(12H)-one (6ak)

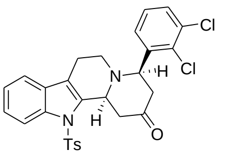

6ak was obtained as a white solid in 45% yield after flash chromatography and the enantiomeric excess was determined to be 92% by HPLC analysis on Chiralpak AD-H column (30% 2-propanol/n-hexane, 1 mL/min), UV 254 nm, t (major) = 8.50 min, t (minor) = 6.36 min; $[\alpha]_D^{25}$ = +56.2 (c 0.156, $CHCl_3$); ^1H NMR (600 MHz, $CDCl_3$) δ 8.13 (d, *J* = 7.0 Hz, 1H), 7.48 (d, *J* = 6.2 Hz, 1H), 7.27 (m, *J* = 42.5, 36.8 Hz, 5H), 6.92 (s, 2H), 6.82 (d, *J* = 5.6 Hz, 2H), 4.84 (s, 1H), 4.55 (d, *J* = 9.9 Hz, 1H), 3.40 (s, 1H), 3.22 – 3.08 (m, 2H), 2.93 (d, *J* = 8.6 Hz, 1H), 2.82 (d, *J* = 15.0 Hz, 2H), 2.69 (d, *J* = 12.0 Hz, 2H), 2.18 (s, 3H). ^{13}C NMR (101 MHz, $CDCl_3$) δ 208.18, 144.81, 142.00, 137.03, 135.38, 134.34, 134.01, 133.42, 130.32, 129.60, 127.08, 126.70, 125.75, 124.91, 124.18, 118.69 (d, *J* = 7.4 Hz), 115.59, 62.57, 50.56, 44.22, 43.18, 40.07, 22.07, 21.51. HRMS (ESI) m/z $(M+H)^+$ calculated for $C_{28}H_{25}Cl_2N_2O_3S$: 539.0958, observed: 539.0959.

(4R,12bS)-4-(2,4-dinitrophenyl)-12-tosyl-1,3,4,6,7,12b-hexahydroindolo[2,3-a]quinolizin-2(12H)-one (6al)

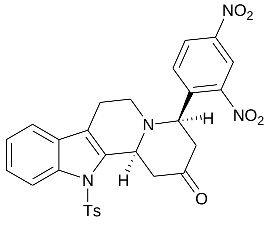

6al was obtained as a white solid in 45% yield after flash chromatography and the enantiomeric excess was determined to be 85% by HPLC analysis on Chiralpak AD-H column (30% 2-propanol/n-hexane, 1 mL/min), UV 254 nm, t (major) = 18.33 min, t (minor) = 31.48 min; $[\alpha]_D^{25}$ = +40.7 (c 0.218,

$CHCl_3$); ^{1}H NMR (600 MHz, cdcl$_3$) δ 8.45 (d, *J* = 2.0 Hz, 1H), 8.42 (m, *J* = 8.5, 2.0 Hz, 1H), 8.02 (d, *J* = 8.2 Hz, 1H), 7.72 (d, *J* = 8.5 Hz, 1H), 7.32 (d, *J* = 7.4 Hz, 1H), 7.25 (d, *J* = 5.3 Hz, 1H), 7.22 (m, *J* = 7.4 Hz, 1H), 7.00 (m, *J* = 20.3, 8.3 Hz, 4H), 5.29 (d, *J* = 5.5 Hz, 1H), 4.24 (d, *J* = 11.0 Hz, 1H), 3.38 (td, *J* = 11.3, 4.0 Hz, 1H), 3.14 – 3.02 (m, 4H), 2.90 – 2.83 (m, 1H), 2.71 (dd, *J* = 15.7, 3.6 Hz, 1H), 2.64 (dd, *J* = 14.7, 11.8 Hz, 1H), 2.30 (s, 3H). ^{13}C NMR (101 MHz, $CDCl_3$) δ 206.00, 144.94, 142.14, 134.15, 130.27, 129.94, 129.67, 126.37, 125.69, 125.16, 124.21, 119.77, 118.75, 118.69, 115.58, 61.02, 52.26, 44.41, 43.56, 38.58, 21.56, 21.21. HRMS (ESI) m/z $(M+H)^+$ calculated for $C_{28}H_{25}N_4O_7S$: 561.1439, observed: 561.1437.

(4R,12bS)-4-(thiophen-2-yl)-12-tosyl-1,3,4,6,7,12b-hexahydroindolo[2,3-a]quinolizin-2(12H)-one (6am: major diastereomer) and (4S,12bS)-4-(thiophen-2-yl)-12-tosyl-1,3,4,6,7,12b-hexahydroindolo[2,3-a]quinolizin-2(12H)-one (7am: minor diastereomer): 6am and 7am were obtained as a white solid in 48% yield after flash chromatography.

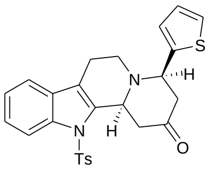

Major diastereomer (6am): the enantiomeric excess was determined to be 89% by HPLC analysis on Chiralpak AD-H column (30% 2-propanol/n-hexane, 1 mL/min), UV 254 nm, t (major) = 16.27 min, t (minor) = 40.16 min; $[\alpha]_D^{25}$ = +83 (c 0.1, $CHCl_3$);^{1}H NMR (600 MHz, $CDCl_3$) δ 8.05 (d, *J* = 8.2 Hz, 1H), 7.54 (d, *J* = 7.0 Hz, 2H), 7.34 (d, *J* = 4.4 Hz, 1H), 7.31 – 7.24 (m, 2H), 7.21 (t, *J* = 7.4 Hz, 1H), 7.13 (d, *J* = 8.1 Hz, 2H), 7.00 (d, *J* = 4.1 Hz, 2H), 4.81 (d, *J* = 9.5 Hz, 1H), 4.60 (d, *J* = 8.8 Hz, 1H), 3.39 (d, *J* = 14.9 Hz, 1H), 2.95 (s, 1H), 2.90 – 2.73 (m, 3H), 2.66 (d, *J* = 14.4 Hz, 3H), 2.29 (s, 3H).^{13}C NMR (101 MHz, $CDCl_3$) δ 206.31, 145.40, 144.82, 137.33, 136.10, 134.49, 130.25, 129.68, 126.57, 125.92, 124.91, 124.81, 124.09, 120.22, 118.67, 115.64, 60.85, 57.79, 44.78, 43.99, 39.29, 22.24, 21.58. HRMS (ESI) m/z $(M+H)^+$ calculated for $C_{26}H_{25}N_2O_3S_2$: 477.1301, observed: 477.1304.

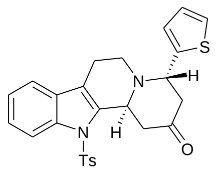

Minor diastereomer (7am): the enantiomeric excess was determined to be 93% by HPLC analysis on Chiralpak AD-H column (30% 2-propanol/n-hexane, 1 mL/min), UV 254 nm, t (major) = 10.89 min, t (minor) = 13.39 min; $[\alpha]_D^{25}$ = +14.9 (c 0.146, $CHCl_3$);^{1}H NMR (600 MHz, $CDCl_3$) δ 8.15 (d, *J* = 8.3 Hz, 1H), 7.33 (d, *J* = 6.8 Hz, 2H), 7.30 – 7.21 (m, 4H), 7.00 (d, *J* = 8.1 Hz, 4H), 4.80 (d, *J* = 5.0 Hz, 1H), 4.73 (d, *J* = 10.6 Hz, 1H), 3.42 (m, *J* = 10.7, 4.1 Hz, 1H), 3.15 (t, *J* = 10.7 Hz, 2H), 2.99 (m, *J* = 17.2, 8.1 Hz, 2H), 2.95 – 2.88 (m, 1H), 2.78 (d, *J* = 14.7 Hz, 1H), 2.60 (dd, *J* = 14.4, 11.8 Hz, 1H), 2.26 (s, 3H).^{13}C NMR

(101 MHz, $CDCl_3$) δ 206.49, 145.78, 144.64, 137.04, 135.77, 134.42, 130.23, 129.59, 127.13, 126.48, 126.26, 125.73, 124.82, 124.04, 118.63, 118.54, 115.59, 61.68, 51.82, 44.55, 44.00, 40.55, 22.36, 21.57. HRMS (ESI) m/z $(M+H)^+$ calculated for $C_{26}H_{25}N_2O_3S_2$: 477.1301, observed: 477.1302.

(4R,12bS)-4-(furan-2-yl)-12-tosyl-1,3,4,6,7,12b-hexahydroindolo[2,3-a]quinolizin-2(12H)-one (6an: major diastereomer) and (4S,12bS)-4-(furan-2-yl)-12-tosyl-1,3,4,6,7,12b-hexahydroin dolo[2,3-a]quinolizin-2(12H)-one (7an: minor diastereomer): 6an and 7an were obtained a s a white solid in 42% yield after flash chromatography

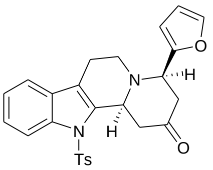

Major diastereomer (6an): the enantiomeric excess was determined to be 96% by HPLC analysis on Chiralpak AD-H column (30% 2-propanol/n-hexane, 1 mL/min), UV 254 nm, t (major) = 13.86 min, t (minor) = 35.90 min; $[\alpha]_D^{25}$ = +10.3 (c 0.112, $CHCl_3$).

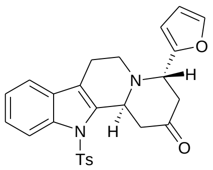

Minor diastereomer (7an): the enantiomeric excess was determined to be 95% by HPLC analysis on Chiralpak IC-H column (30% 2-propanol/n-hexane, 1 mL/min), UV 254 nm, t (major) = 21.55 min, t (minor) = 16.81 min; $[\alpha]_D^{25}$ = +11.8 (c 0.126, $CHCl_3$);^{1}H NMR (600 MHz, $CDCl_3$) δ 8.15 (d, J = 8.2 Hz, 1H), 7.48 (s, 1H), 7.29 (m, J = 14.9, 7.5 Hz, 2H), 7.25 – 7.17 (m, 3H), 6.97 (d, J = 7.6 Hz, 2H), 6.43 (s, 1H), 6.32 (s, 1H), 4.64 (d, J = 6.4 Hz, 1H), 4.47 (d, J = 10.9 Hz, 1H), 3.37 (s, 1H), 3.25 (d, J = 15.0 Hz, 1H), 3.06 – 3.00 (m, 1H), 2.94 (dd, J = 14.7, 6.3 Hz, 1H), 2.80 (dt, J = 27.0, 11.8 Hz, 3H), 2.57 – 2.50 (m, 1H), 2.24 (s, 3H).^{13}C NMR (101 MHz, $CDCl_3$) δ 206.57, 153.42, 144.74, 142.37, 137.43, 135.82, 134.29, 130.43, 129.55, 126.18, 124.88, 124.19, 119.43, 118.53, 115.88, 110.52, 109.65, 59.31, 51.88, 45.14, 44.94, 40.21, 22.30, 21.54. HRMS (ESI) m/z $(M+H)^+$ calculated for $C_{26}H_{25}N_2O_4S$: 461.1530, observed: 461.1533.

(4R,12bS)-4-(2-methylprop-1-enyl)-12-tosyl-1,3,4,6,7,12b-hexahydroindolo[2,3-a]quinolizin-2(12H)-one (6ao)

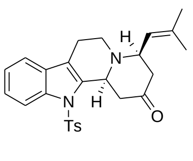

6ao was obtained as a white solid in 24% yield after flash chromatography and the enantiomeric excess was determined to be 85% by HPLC analysis on Chiralpak AD-H column (10% 2-propanol/n-hexane, 1 mL/min), UV 254 nm, t (major) = 23.89 min, t (minor) = 21.46 min; $[\alpha]_D^{25}$ = +29.0 (c 0.272, $CHCl_3$); ^{1}H NMR (600 MHz, $CDCl_3$) δ

8.02 (d, J = 7.8 Hz, 1H), 7.44 (d, J = 7.1 Hz, 2H), 7.24 (m, J = 19.1, 7.5 Hz, 2H), 7.18 (d, J = 6.9 Hz, 1H), 7.03 (d, J = 7.2 Hz, 2H), 5.19 (d, J = 6.4 Hz, 1H), 4.38 (d, J = 8.9 Hz, 1H), 3.79 (s, 1H), 3.40 (d, J = 14.4 Hz, 1H), 3.28 (s, 1H), 2.71 (s, 1H), 2.67 – 2.54 (m, 2H), 2.52 – 2.45 (m, 1H), 2.40 (s, 2H), 2.22 (s, 3H), 1.78 (s, 3H), 1.72 (s, 3H). ^{13}C NMR (101 MHz, $CDCl_3$) δ 207.49, 144.70, 138.02, 133.74, 130.70, 129.41, 126.60, 125.01, 124.90, 124.33, 122.22, 118.64, 116.18, 59.82, 59.09, 46.52, 45.91, 42.18, 26.01, 22.66, 21.54, 18.61. HRMS (ESI) m/z $(M+H)^+$ calculated for $C_{28}H_{25}N_4O_7S$: 449.1894, observed: 449.1896.

5. Reference

[1] Viviano M,; Glasnov T N,; Reichart B.; Tekautz, G.; Kappe, C. O., A Scalable Two-Step Continuous Flow Synthesis of Nabumetone and Related 4-Aryl-2-butanones. *Org. Process Res. Dev*. **2011**, 15, *4*, 858-870.

[2] Lalonde, M. P.; McGowan, M. A.; Rajapaksa, N. S.; Jacobsen, E. N., Enantioselective formal aza-Diels-Alder reactions of enones with cyclic imines catalyzed by primary aminothioureas. *J. Am. Chem. Soc.* **2013,** *135*, 1891-1894.

[3] Cochrane E. J.; Hassall, L. A.; Coldham, I., Preparation of 1-Substituted Tetrahydro-beta-carbolines by Lithiation-Substitution. *J. Org. Chem.*, **2015**, *80*, 5964-5969.

[4] Cole, D. C.; Lennox, W. J.; Stock, J. R.; Ellingboe, J. W.; Mazandarani, H.; Smith, D. L. Zhang, G.; Tawa, G. J. Schechter, L. E. Conformationally constrained N1-arylsulfonyltryptamine derivatives as 5-HT6 receptor antagonists. *Bioorg. Med. Chem. Lett.*, **2005**, *15*, 4780-4785.

6. ^{1}H NMR and ^{13}C NMR Spectra for the Products

(4R,12bS)-4-(4-methoxyphenyl)-12-tosyl-1,3,4,6,7,12b-hexahydroindolo[2,3-a]quinolizin-2(12H)-one (6aa)

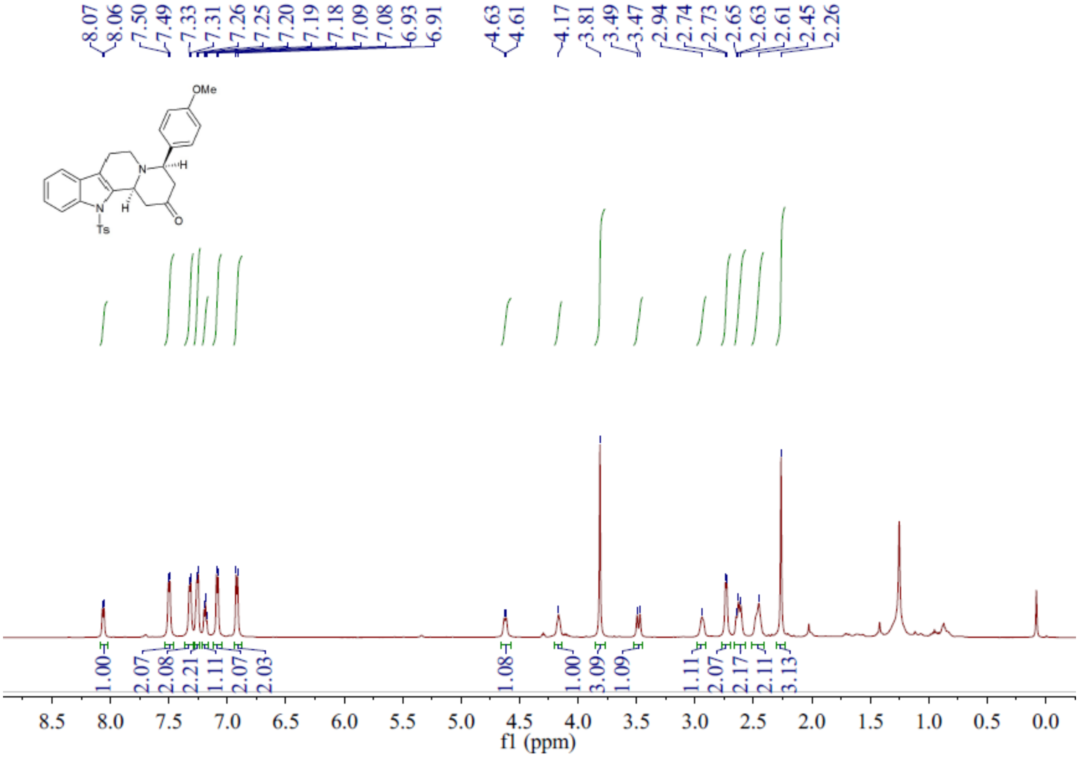

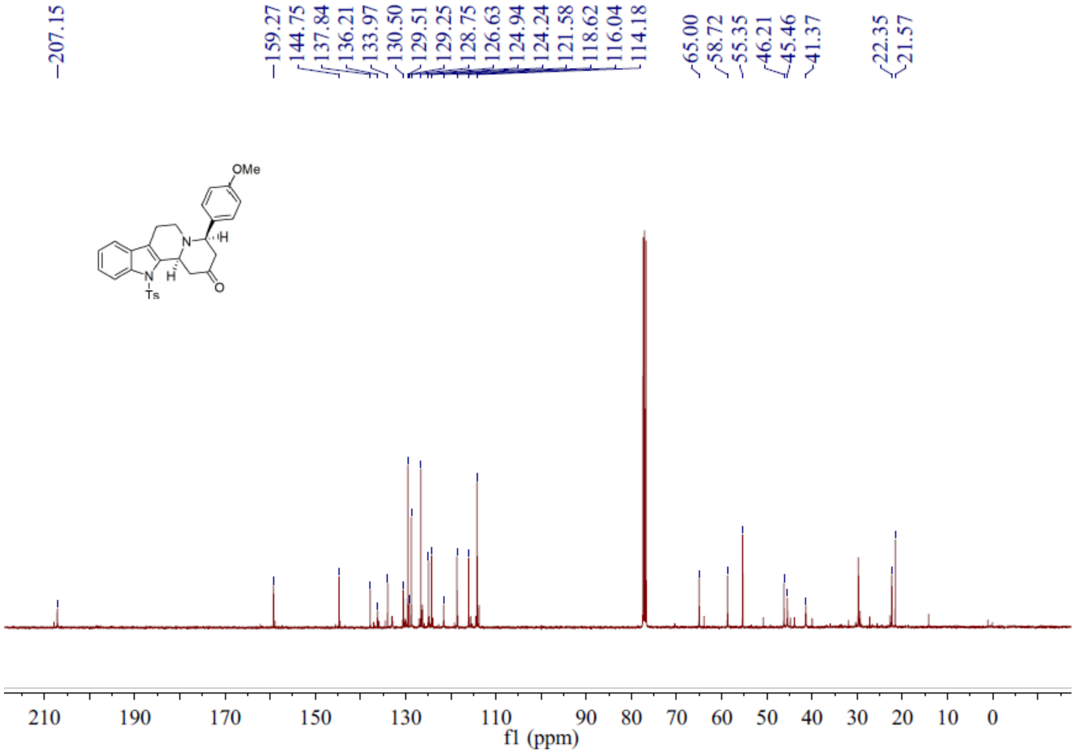

(4S,12bS)-4-(4-methoxyphenyl)-12-tosyl-1,3,4,6,7,12b-hexahydroindolo[2,3-a]quinolizin-2(12H)-one (7aa)

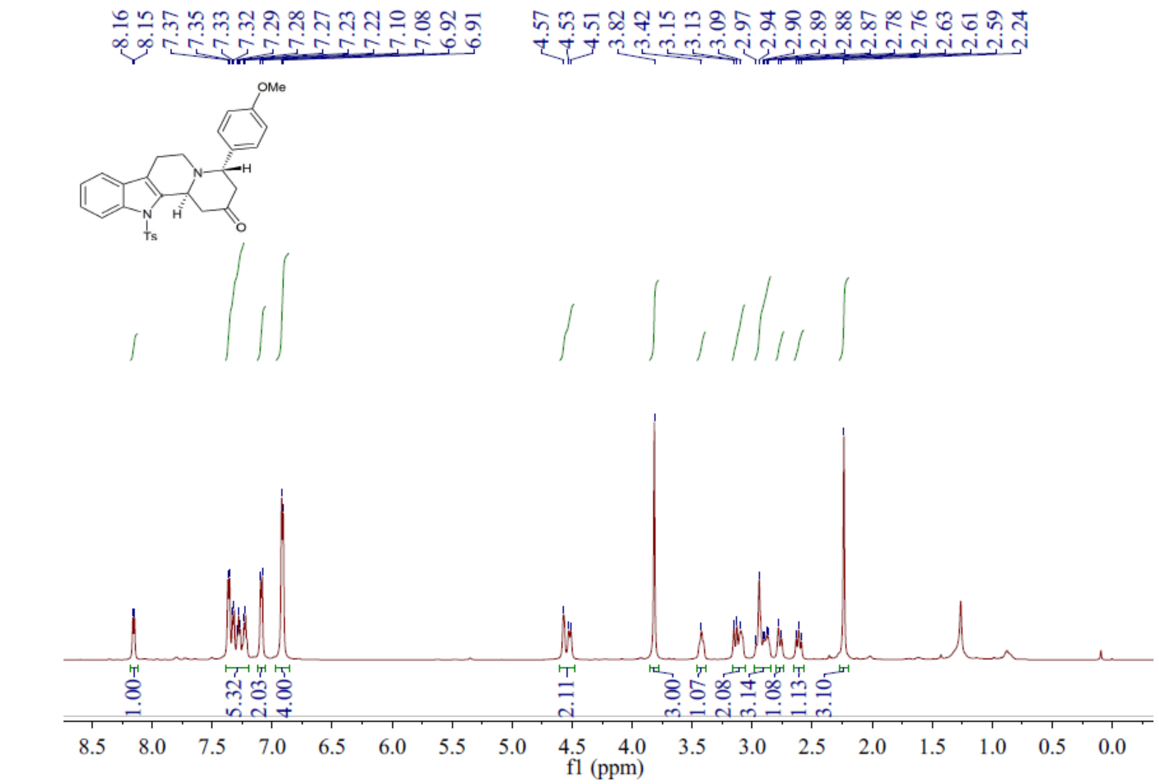

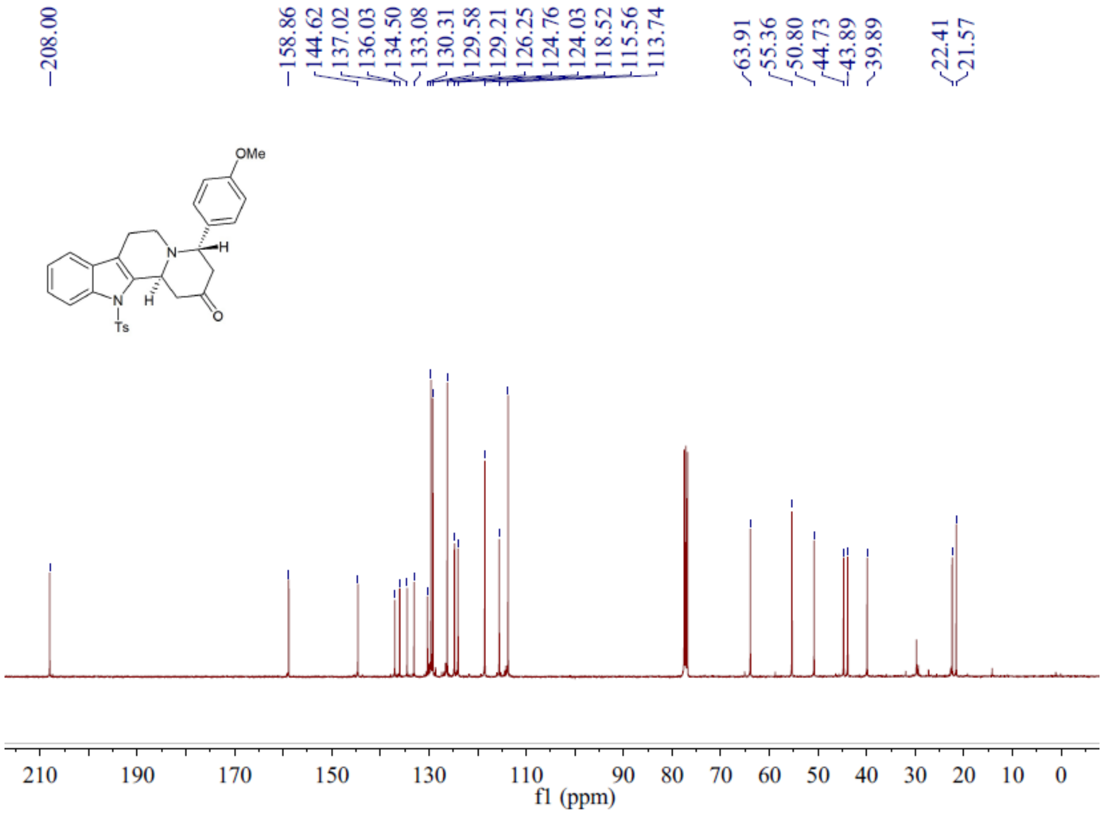

(4R,12bS)-4-phenyl-12-tosyl-1,3,4,6,7,12b-hexahydroindolo[2,3-a]quinolizin-2(12H)-one (6ab)

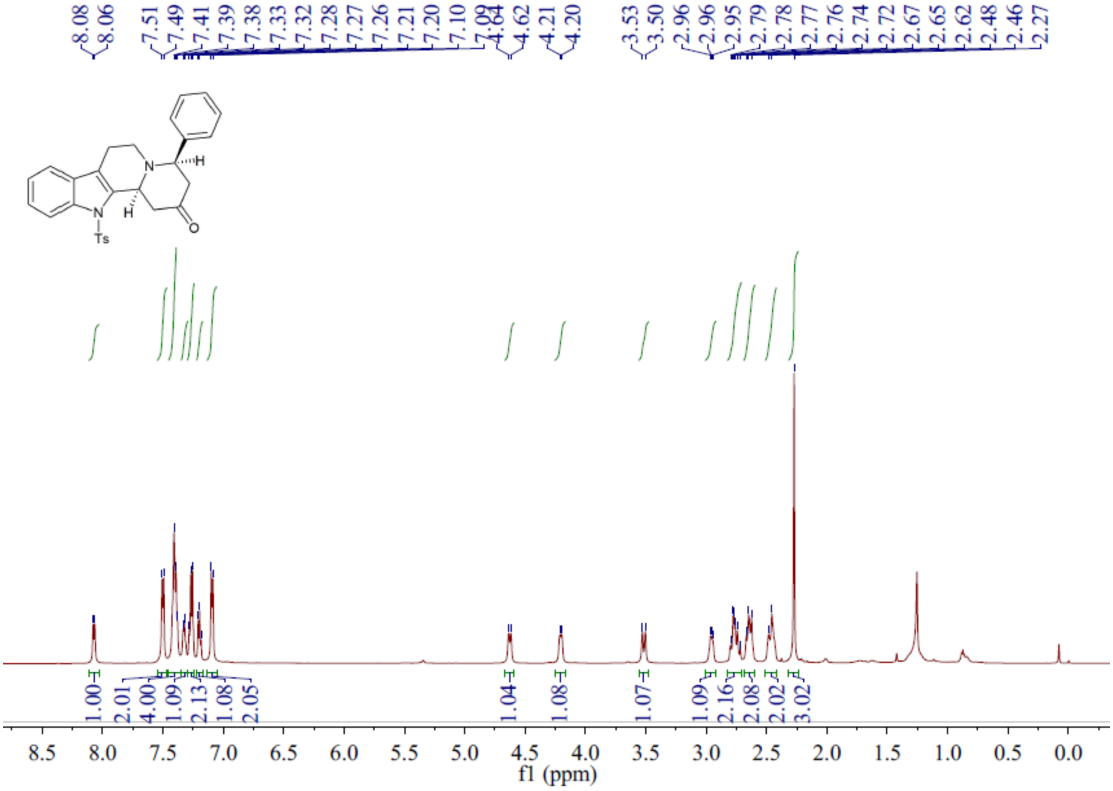

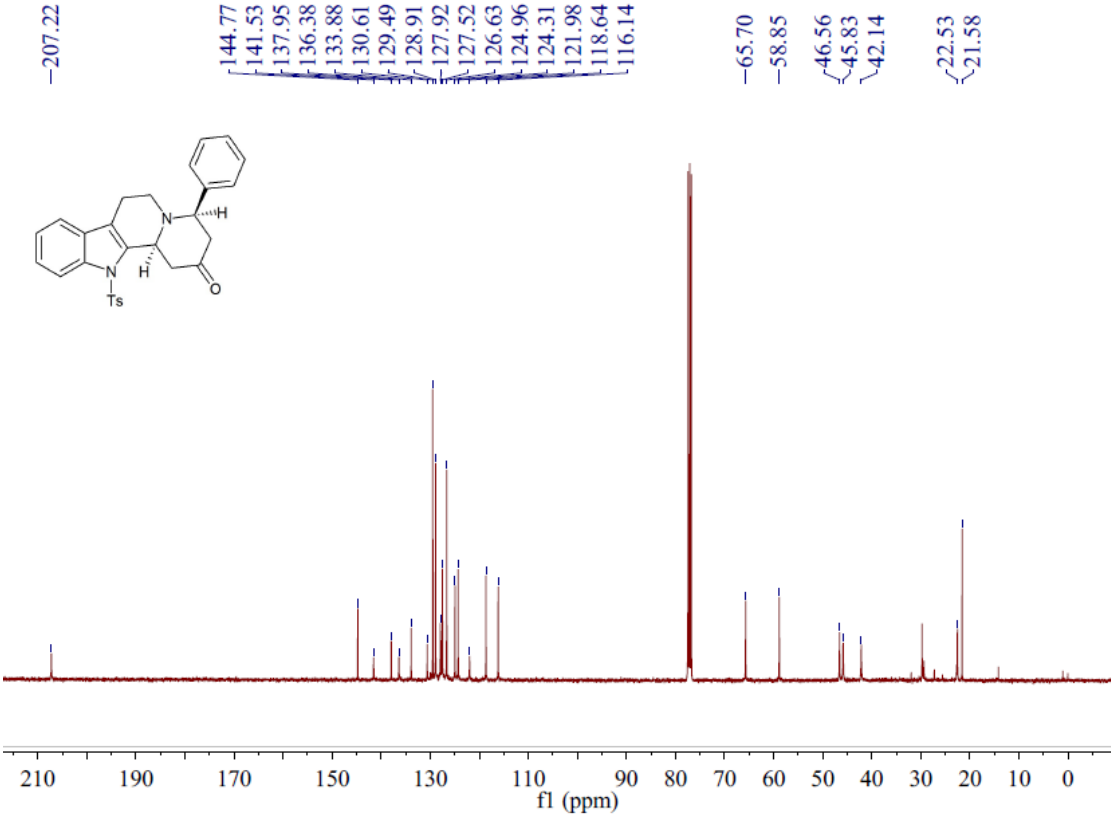

(4S,12bS)-4-phenyl-12-tosyl-1,3,4,6,7,12b-hexahydroindolo[2,3-a]quinolizin-2(12H)-one (7ab)

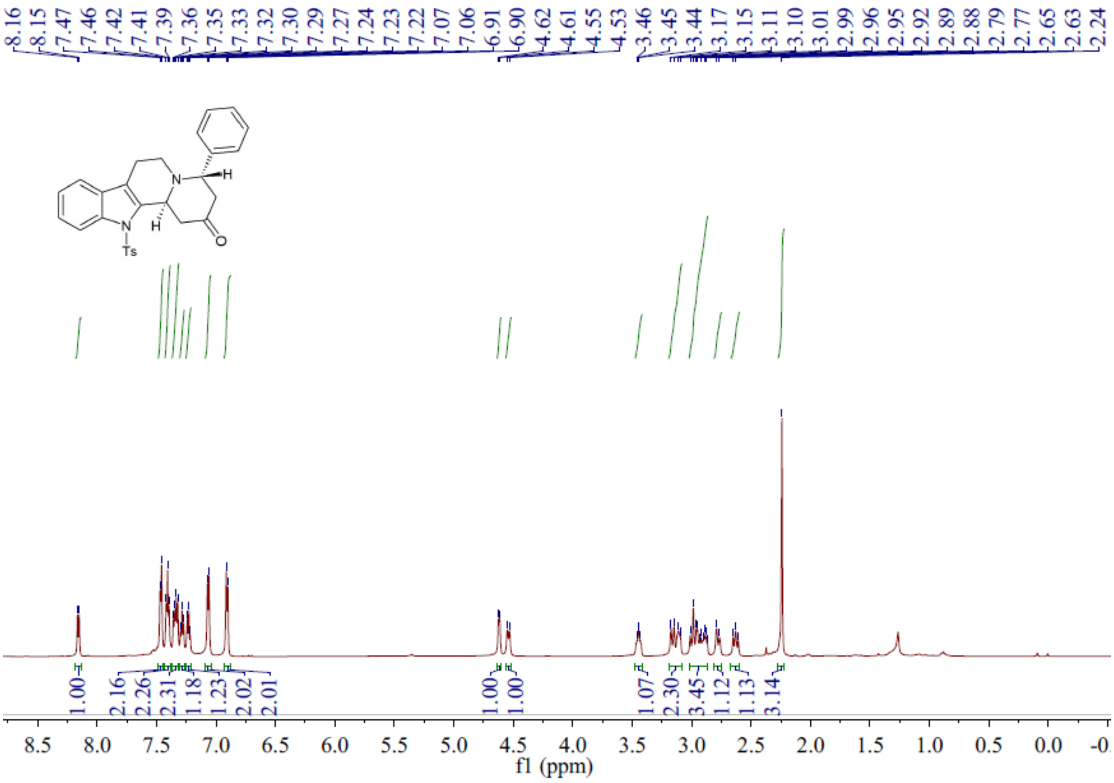

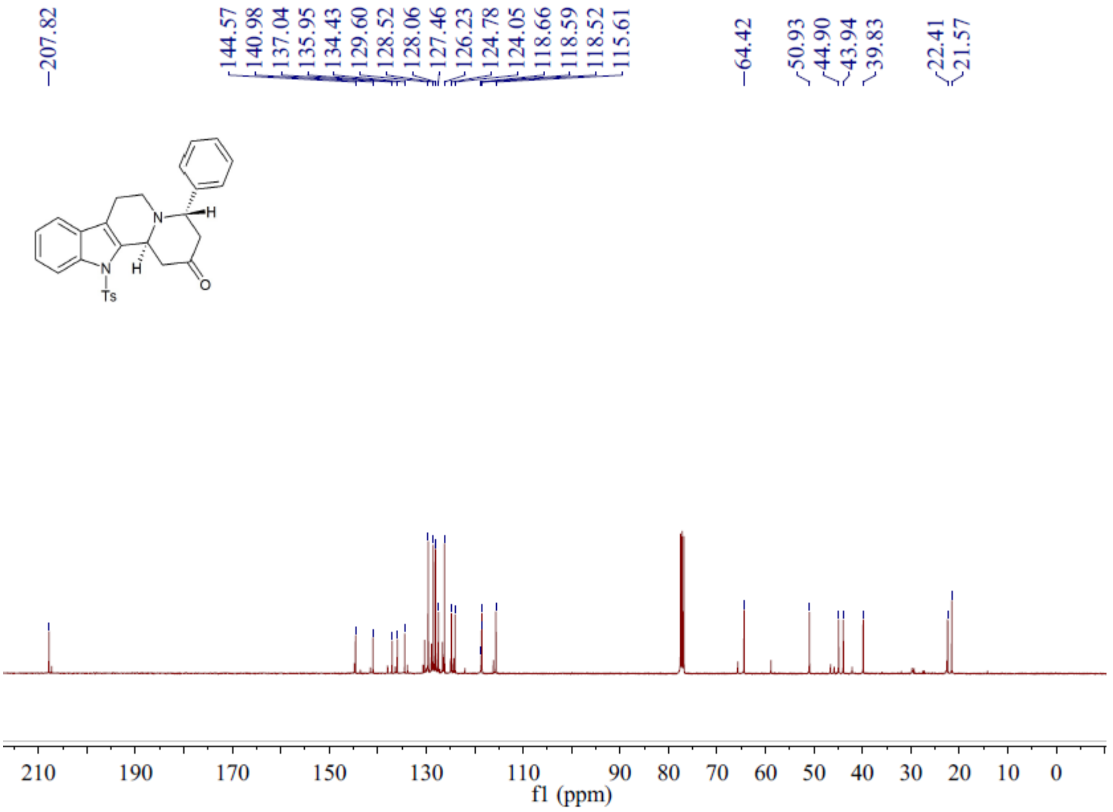

4R,12bS)-4-o-tolyl-12-tosyl-1,3,4,6,7,12b-hexahydroindolo[2,3-a]quinolizin-2(12H)-one (6ac)

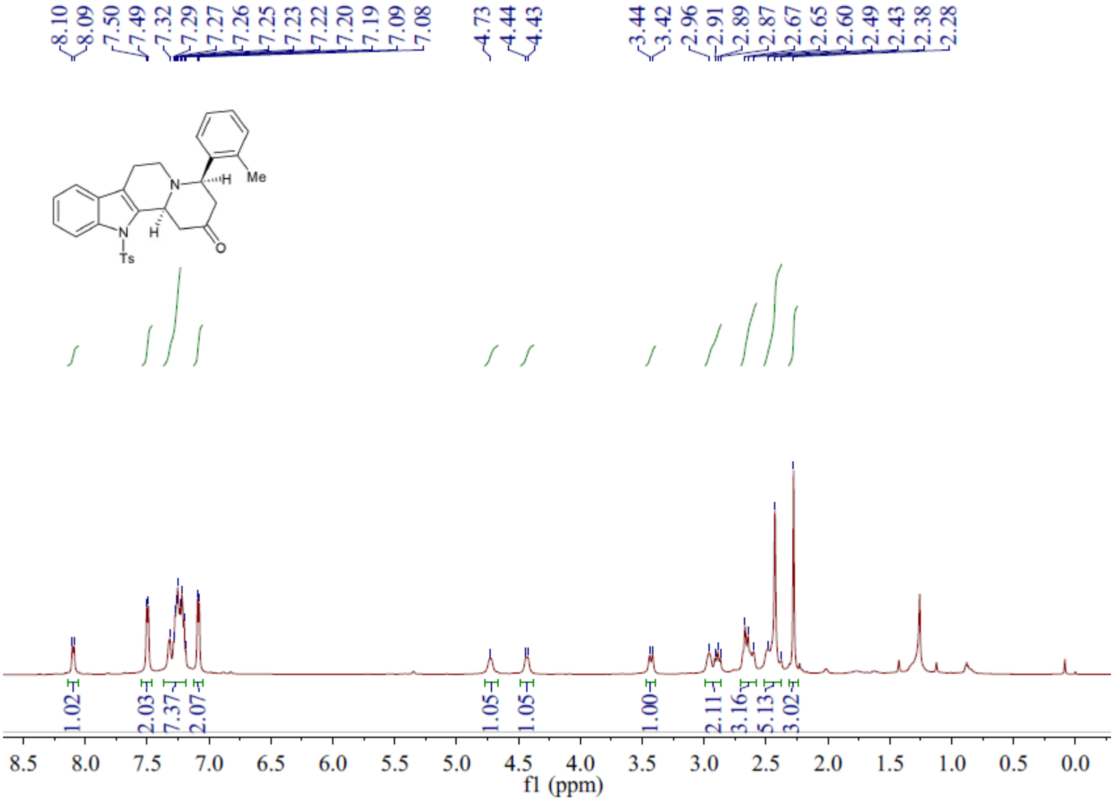

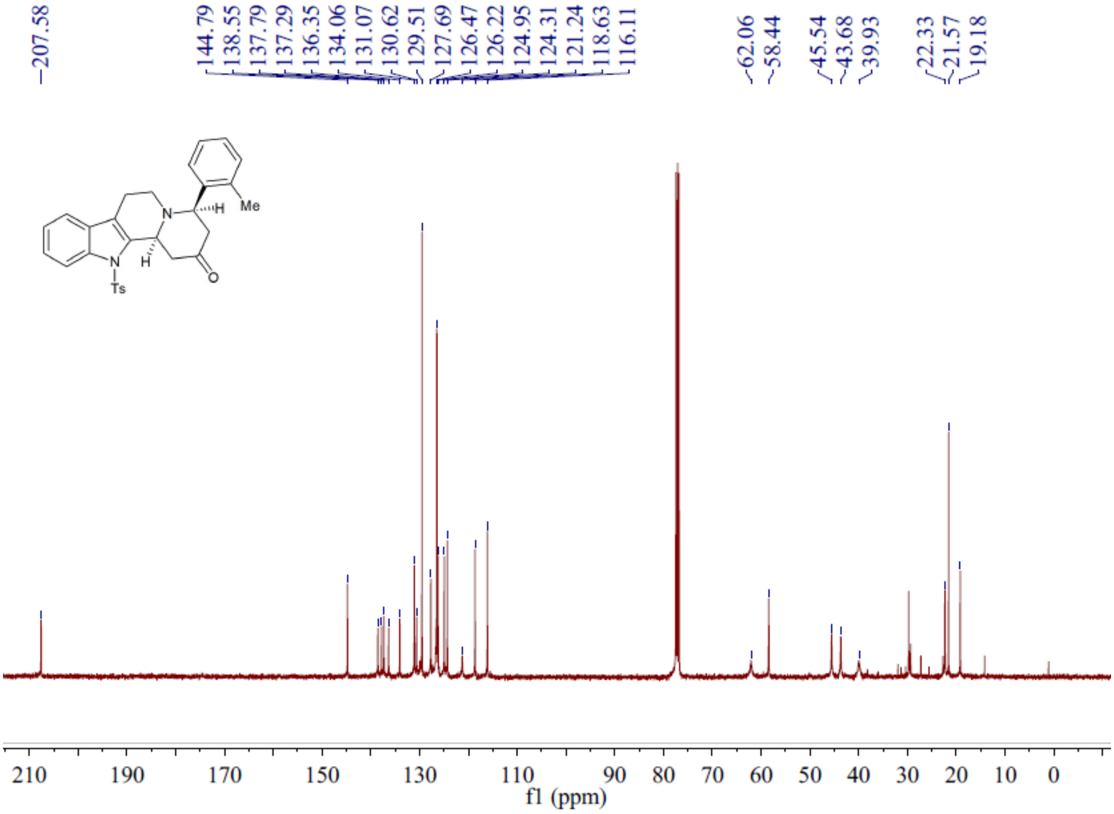

(4S,12bS)-4-o-tolyl-12-tosyl-1,3,4,6,7,12b-hexahydroindolo[2,3-a]quinolizin-2(12H)-one (7ac)

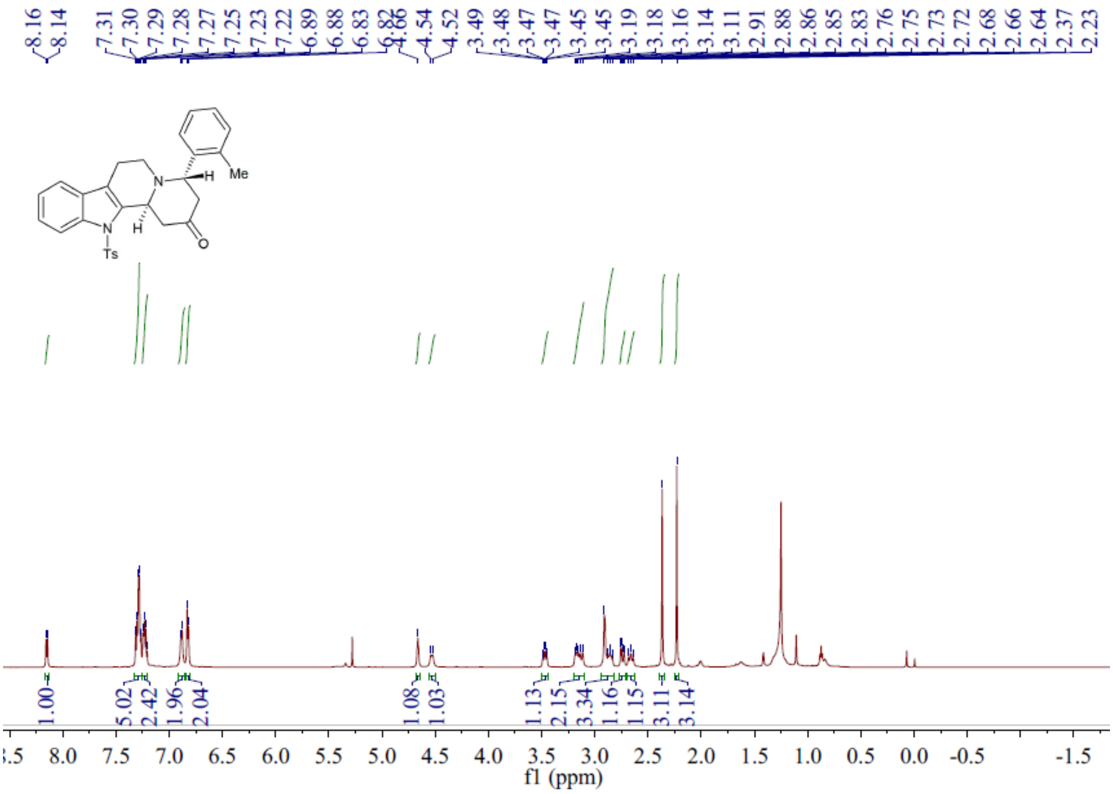

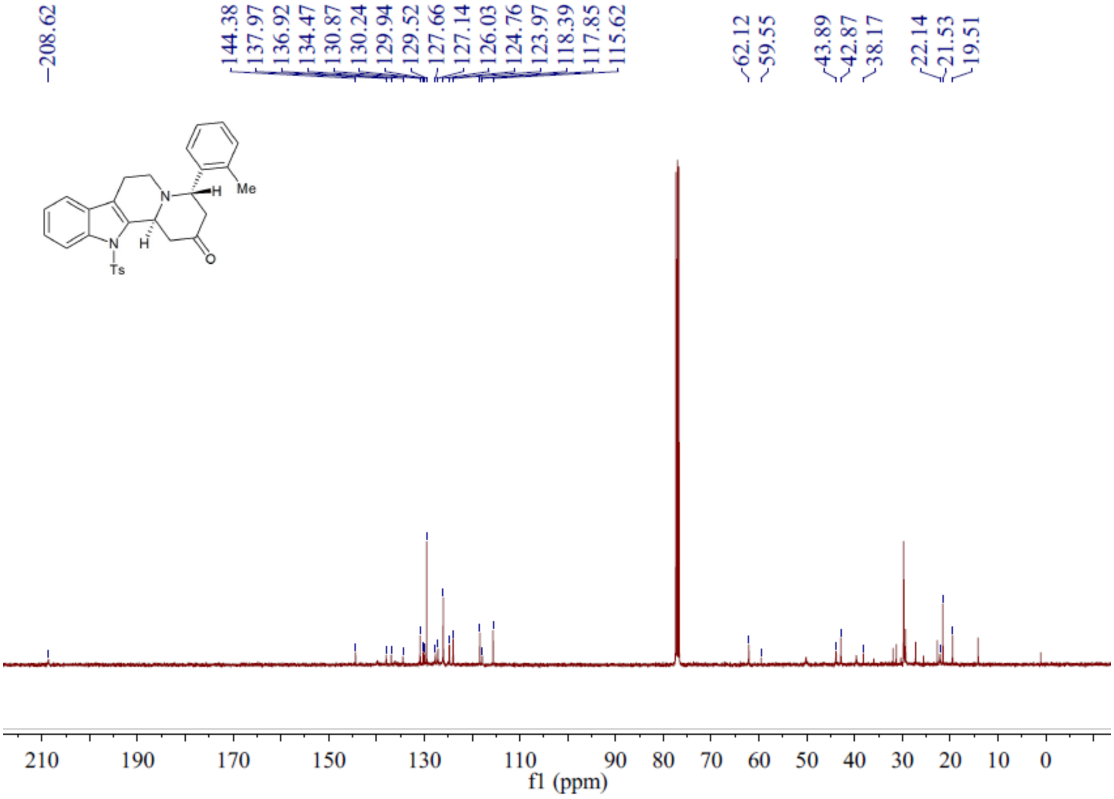

(4R,12bS)-4-m-tolyl-12-tosyl-1,3,4,6,7,12b-hexahydroindolo[2,3-a]quinolizin-2(12H)-one (6ad)

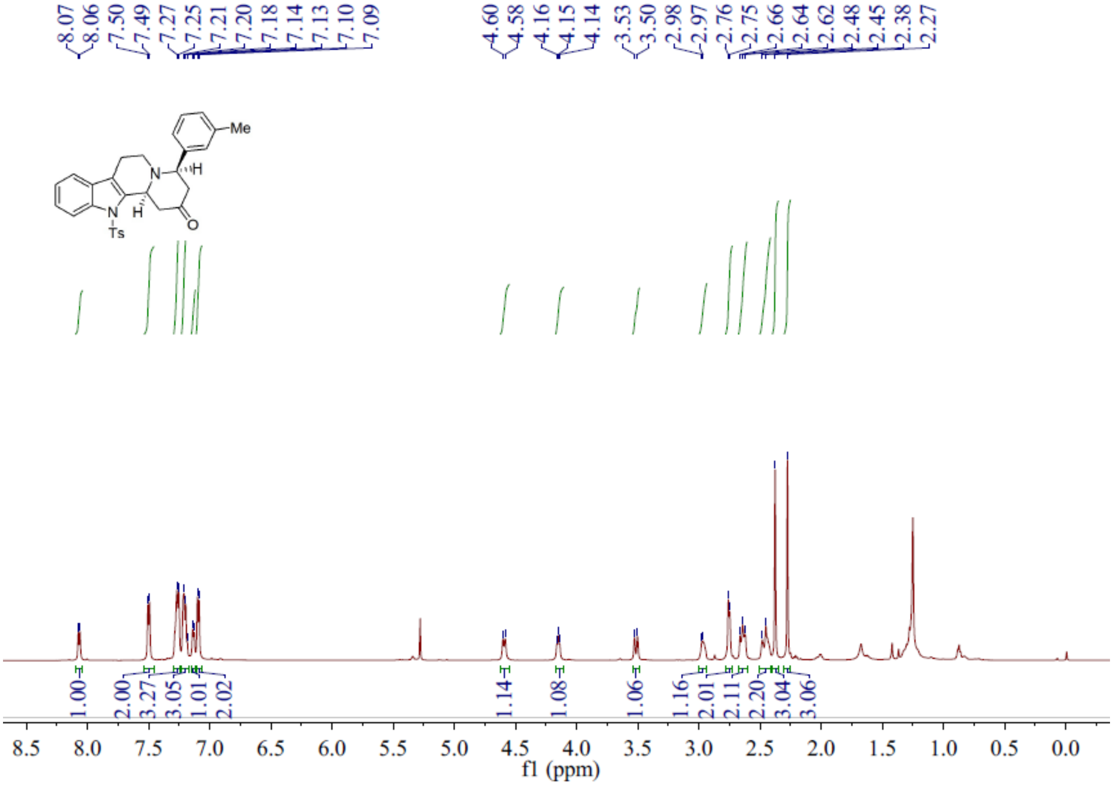

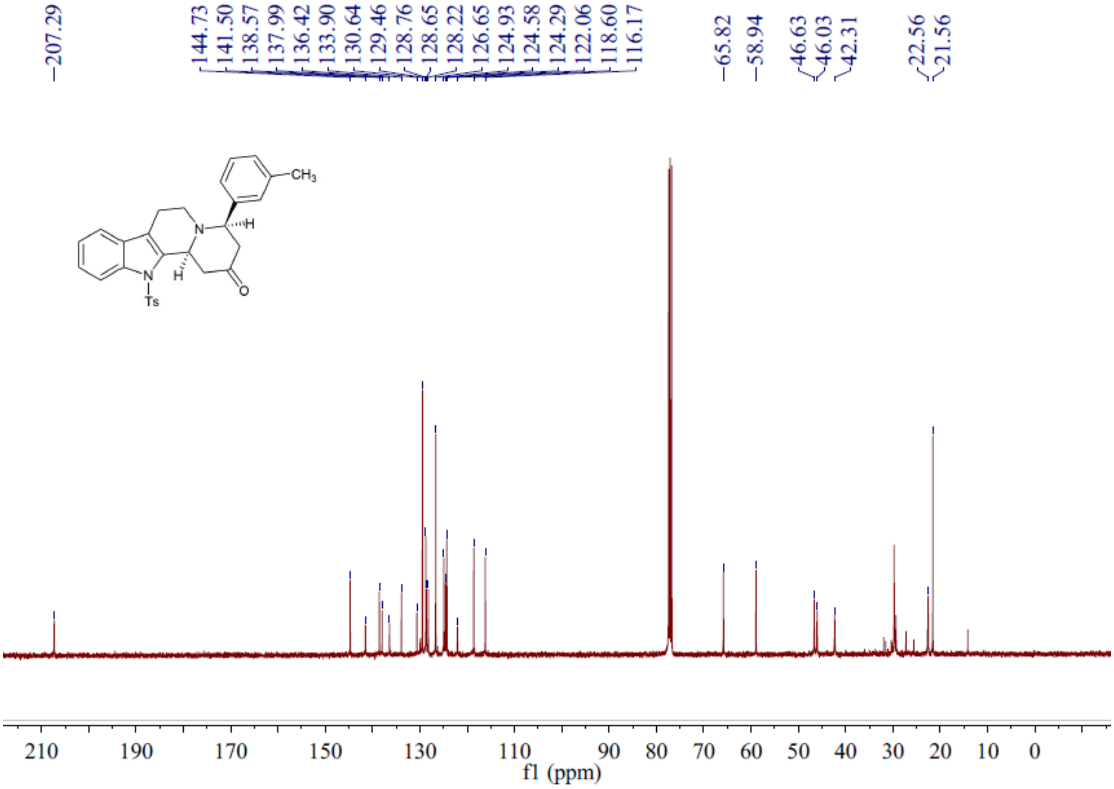

(4S,12bS)-4-m-tolyl-12-tosyl-1,3,4,6,7,12b-hexahydroindolo[2,3-a]quinolizin-2(12H)-one (7ad)

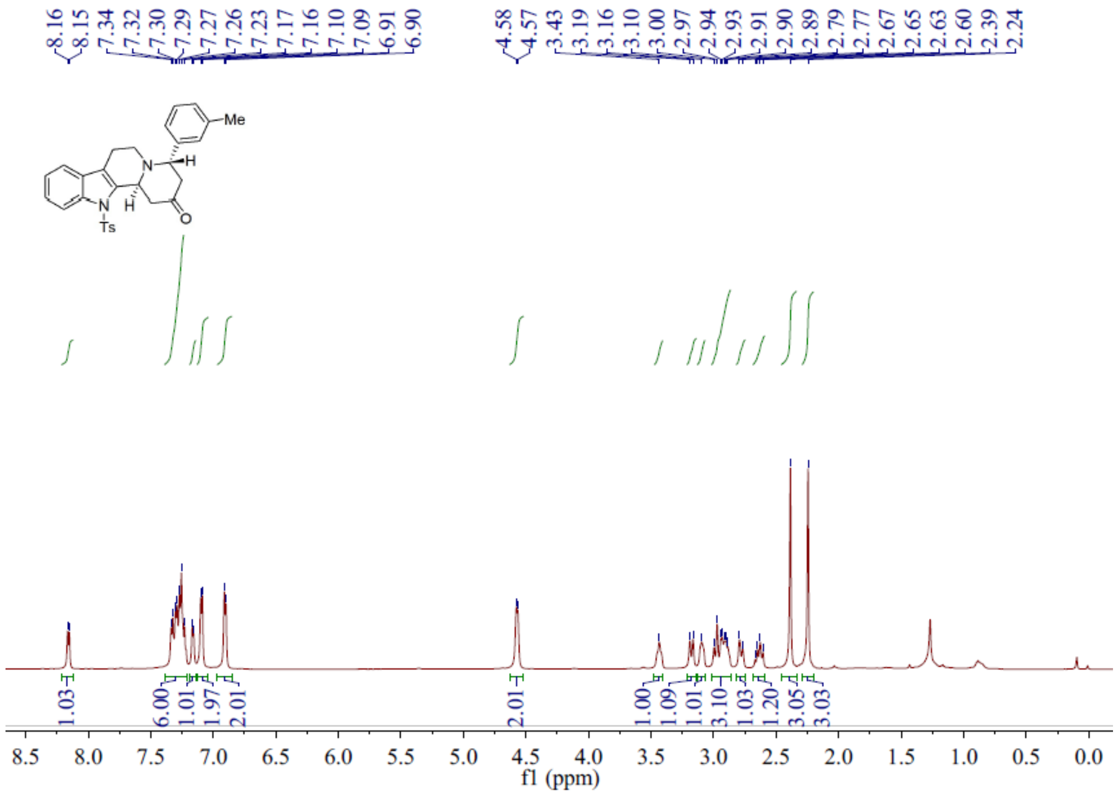

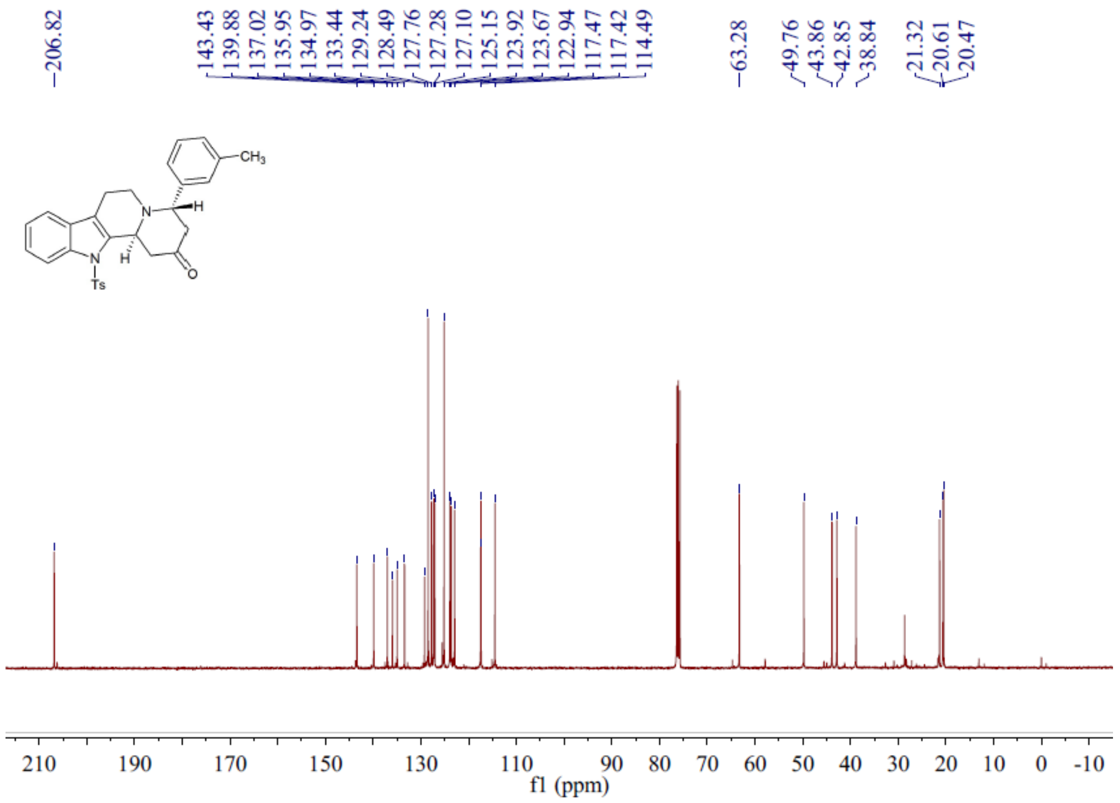

(4R,12bS)-4-p-tolyl-12-tosyl-1,3,4,6,7,12b-hexahydroindolo[2,3-a]quinolizin-2(12H)-one (6ae)

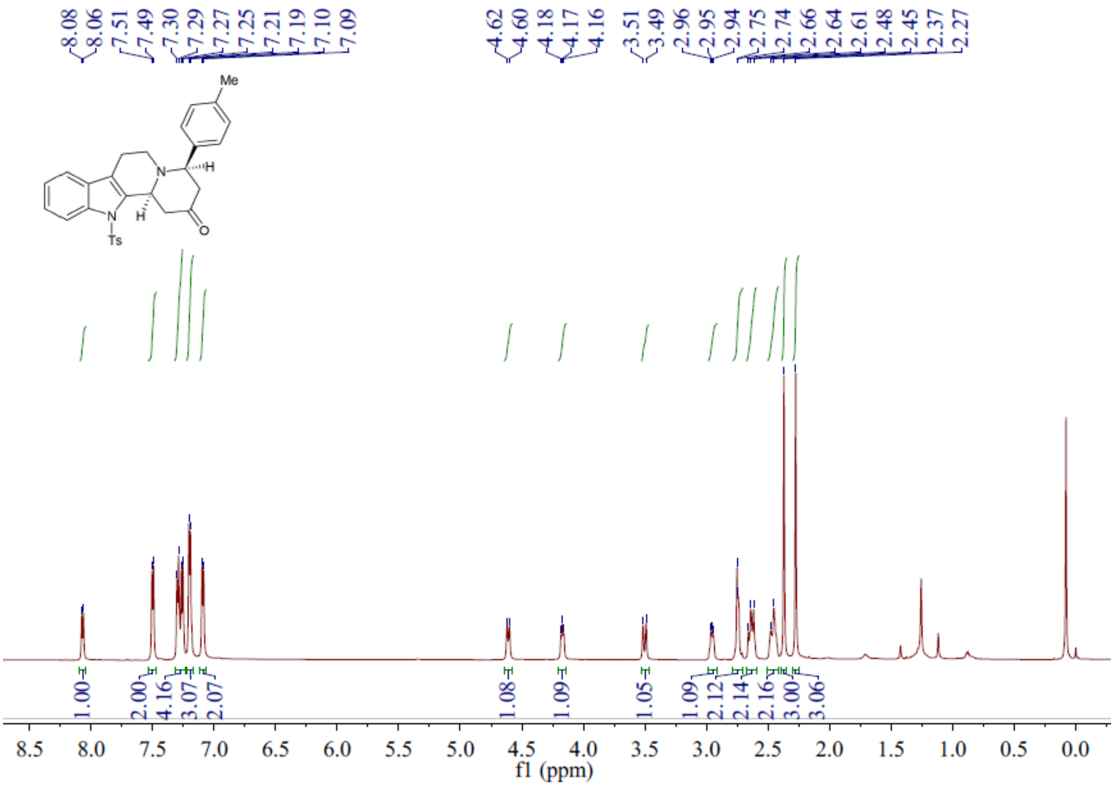

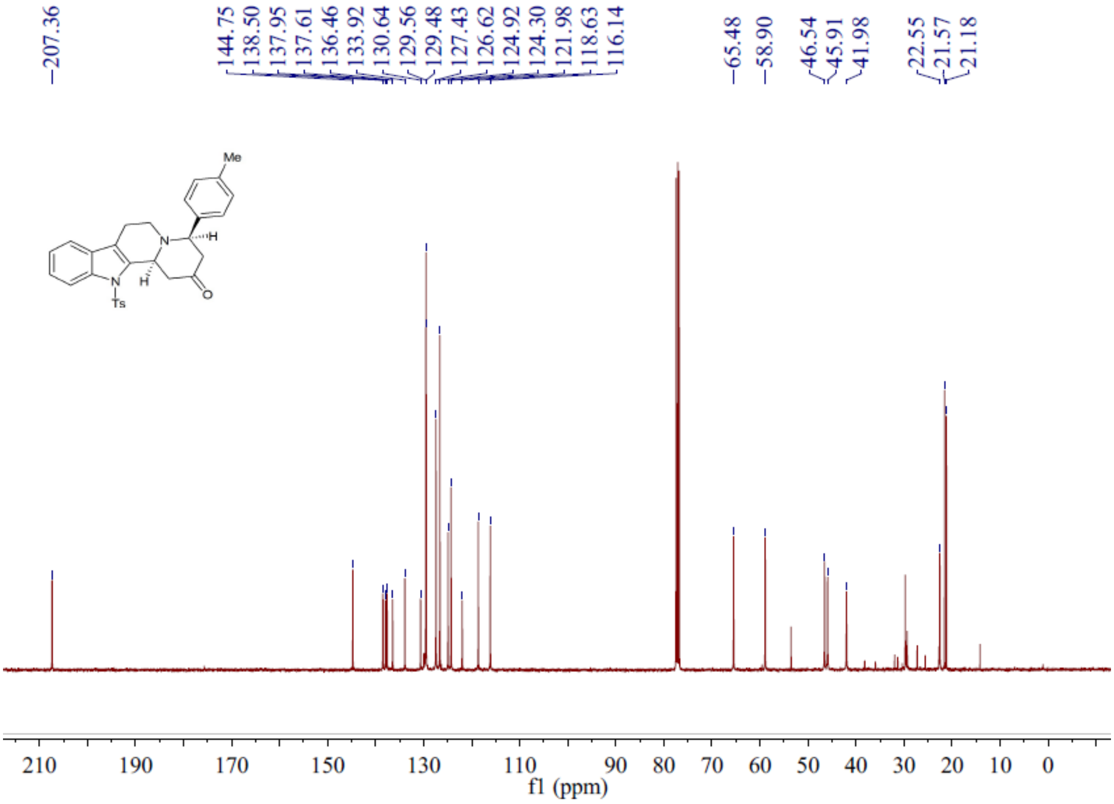

(4S,12bS)-4-p-tolyl-12-tosyl-1,3,4,6,7,12b-hexahydroindolo[2,3-a]quinolizin-2(12H)-one (7ae)

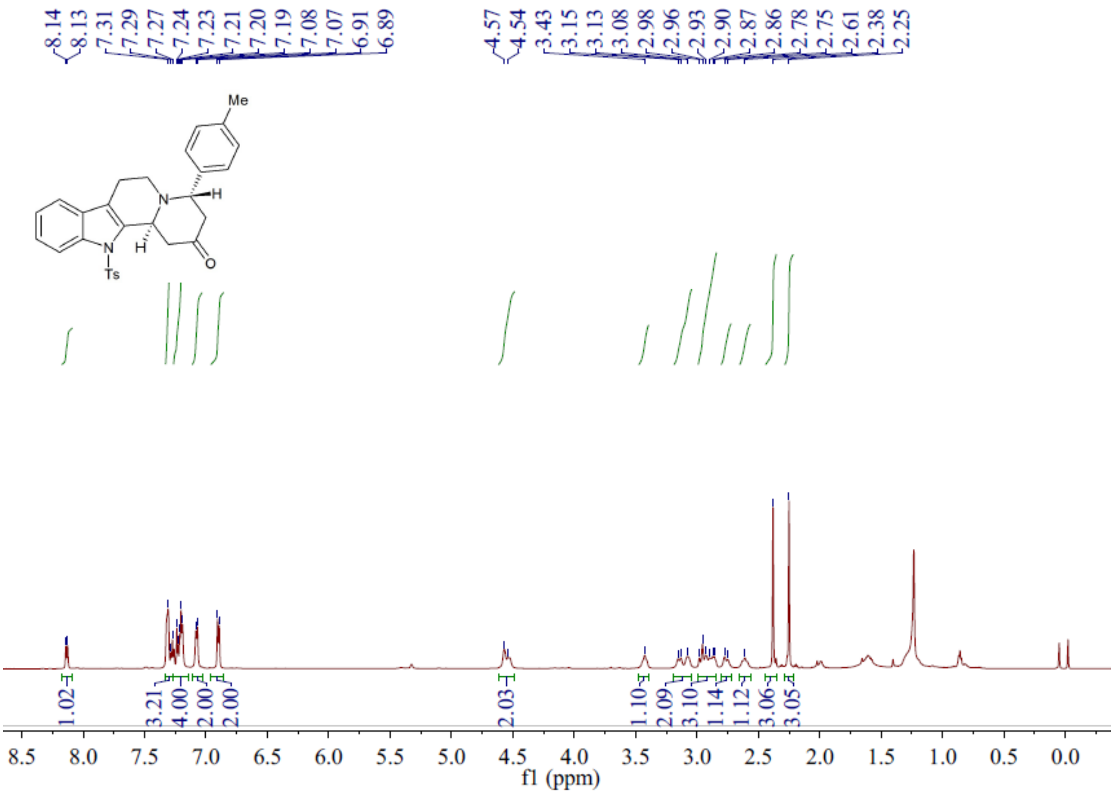

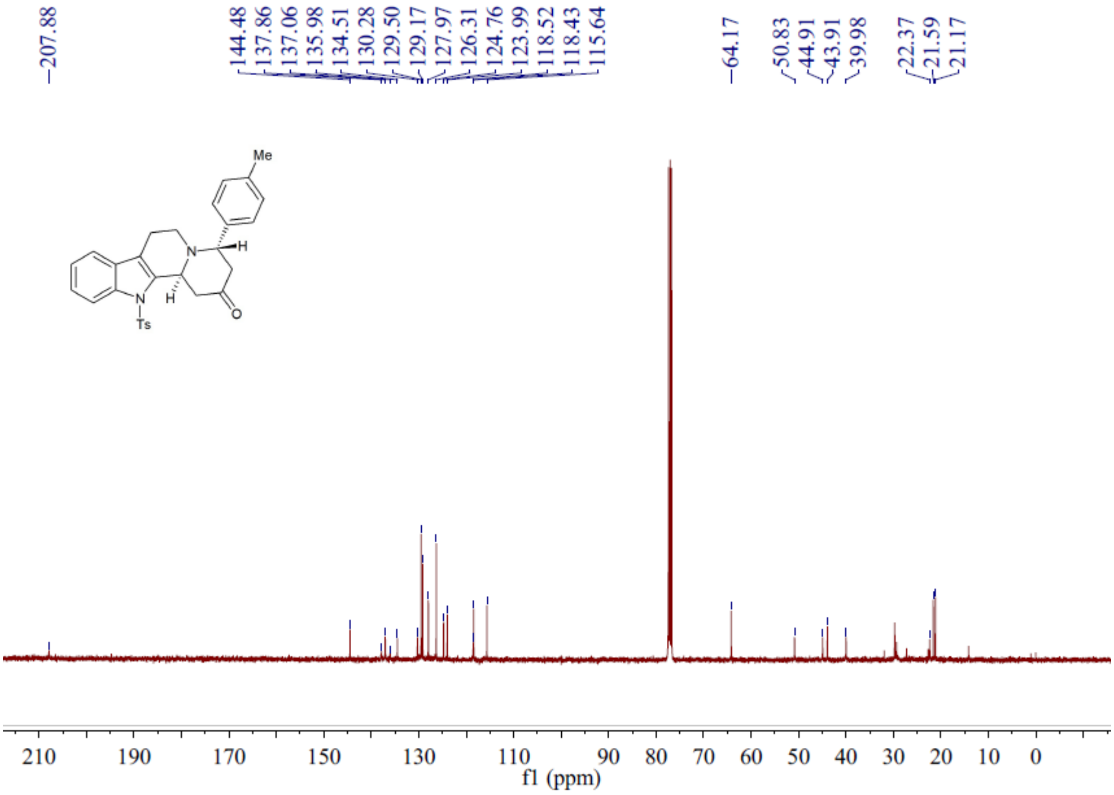

(4R,12bS)-4-(4-chlorophenyl)-12-tosyl-1,3,4,6,7,12b-hexahydroindolo[2,3-a]quinolizin-2(12H)-one (6af)

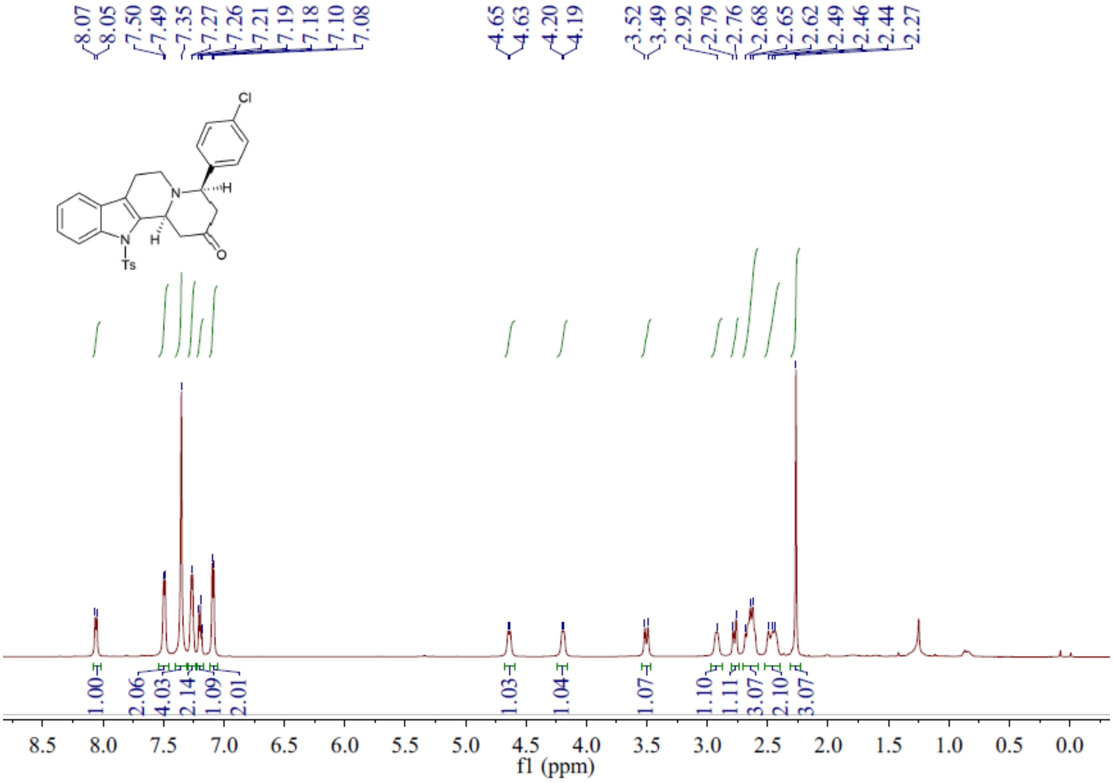

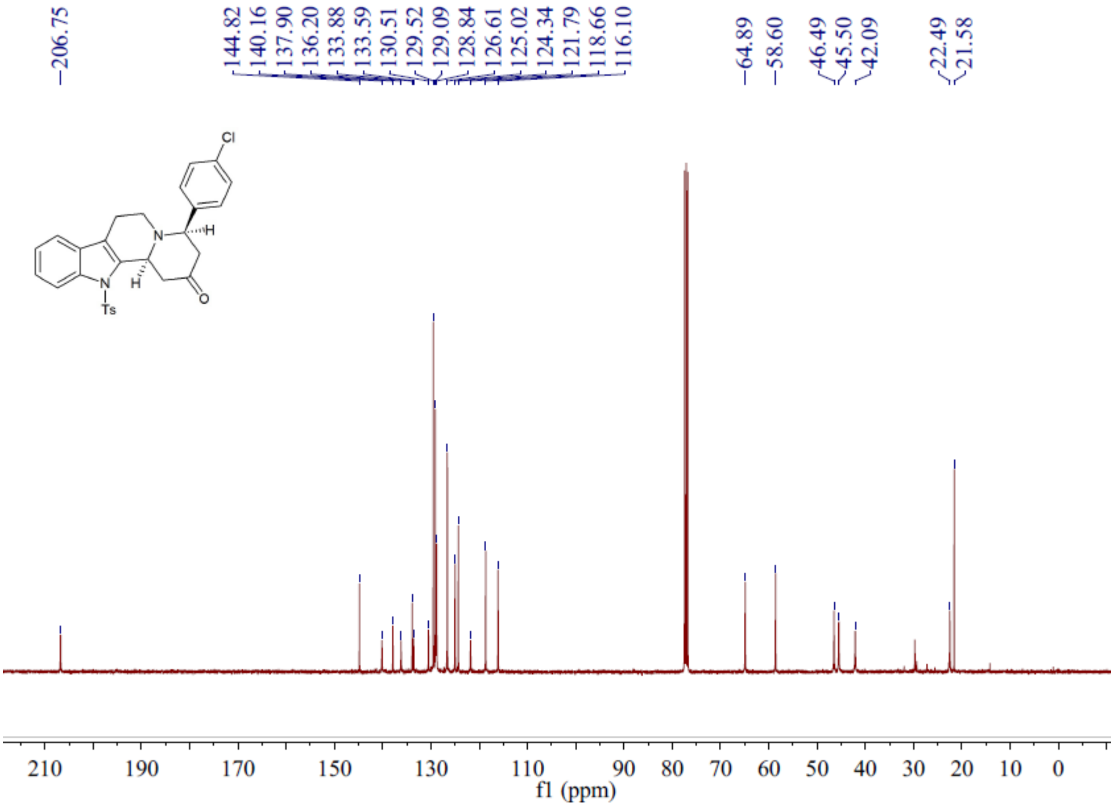

(4S,12bS)-4-(4-chlorophenyl)-12-tosyl-1,3,4,6,7,12b-hexahydroindolo[2,3-a]quinolizin-2(12H)-one (7af)

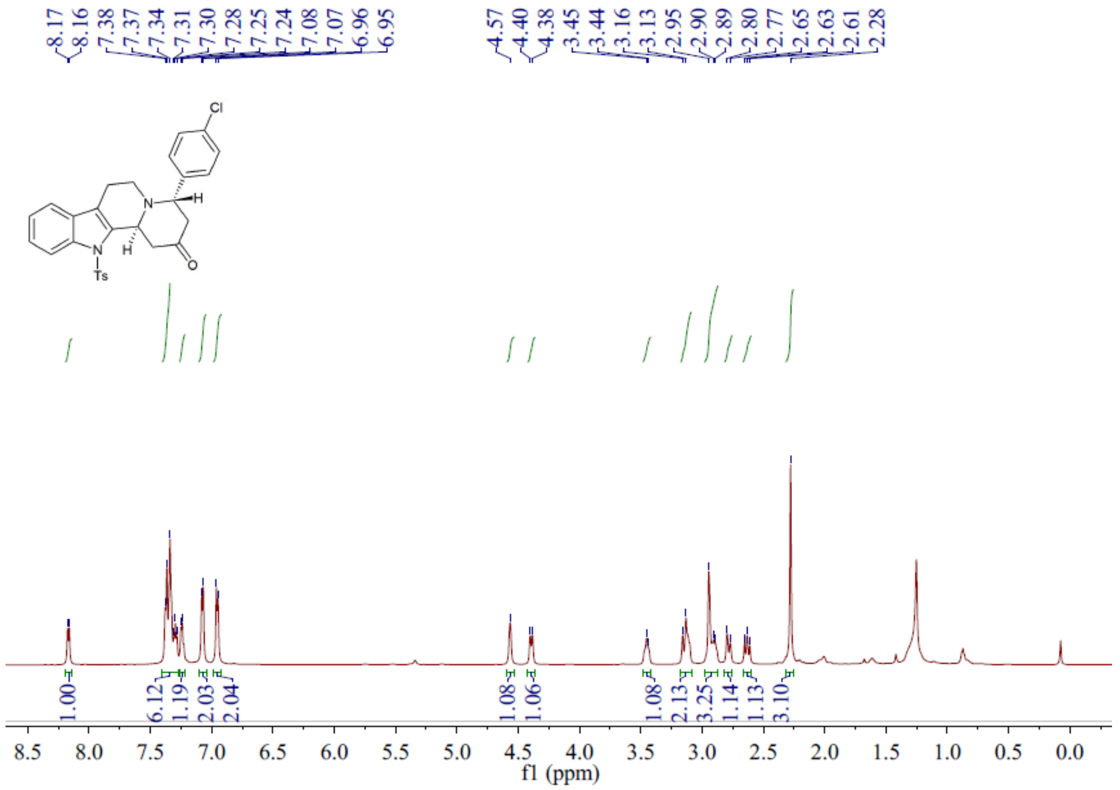

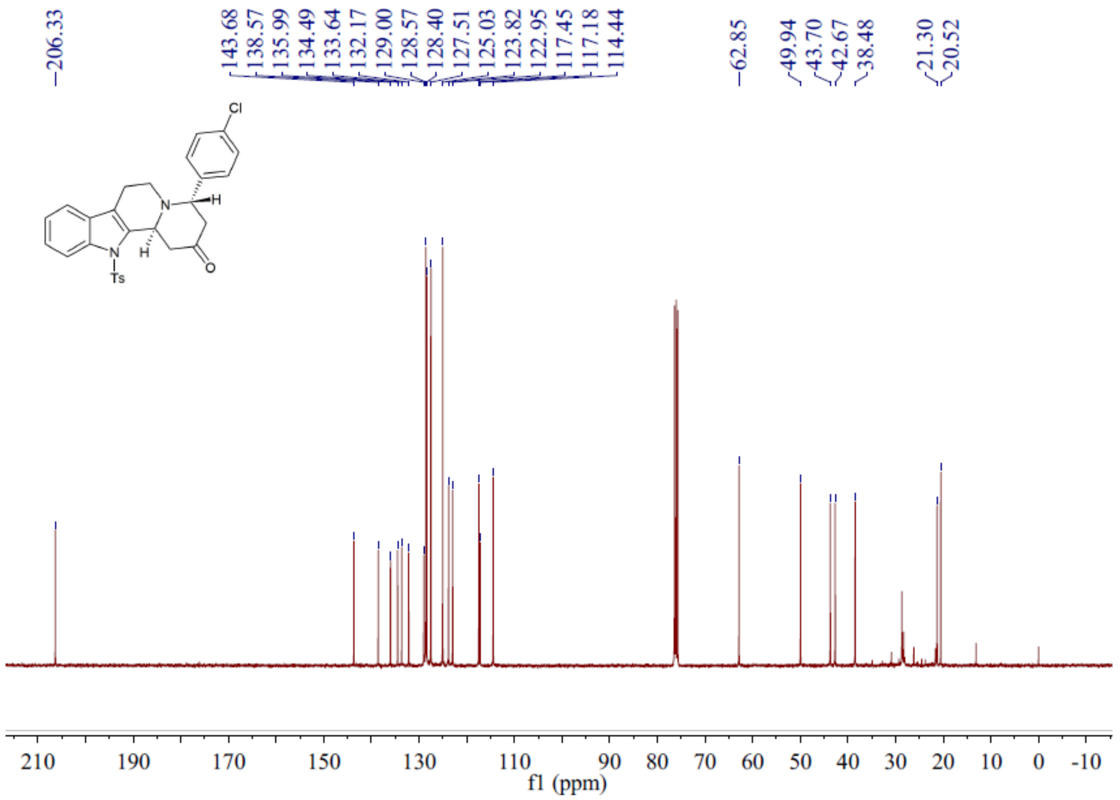

(4R,12bS)-4-(4-bromophenyl)-12-tosyl-1,3,4,6,7,12b-hexahydroindolo[2,3-a]quinolizin-2(12H)-one (6ag)

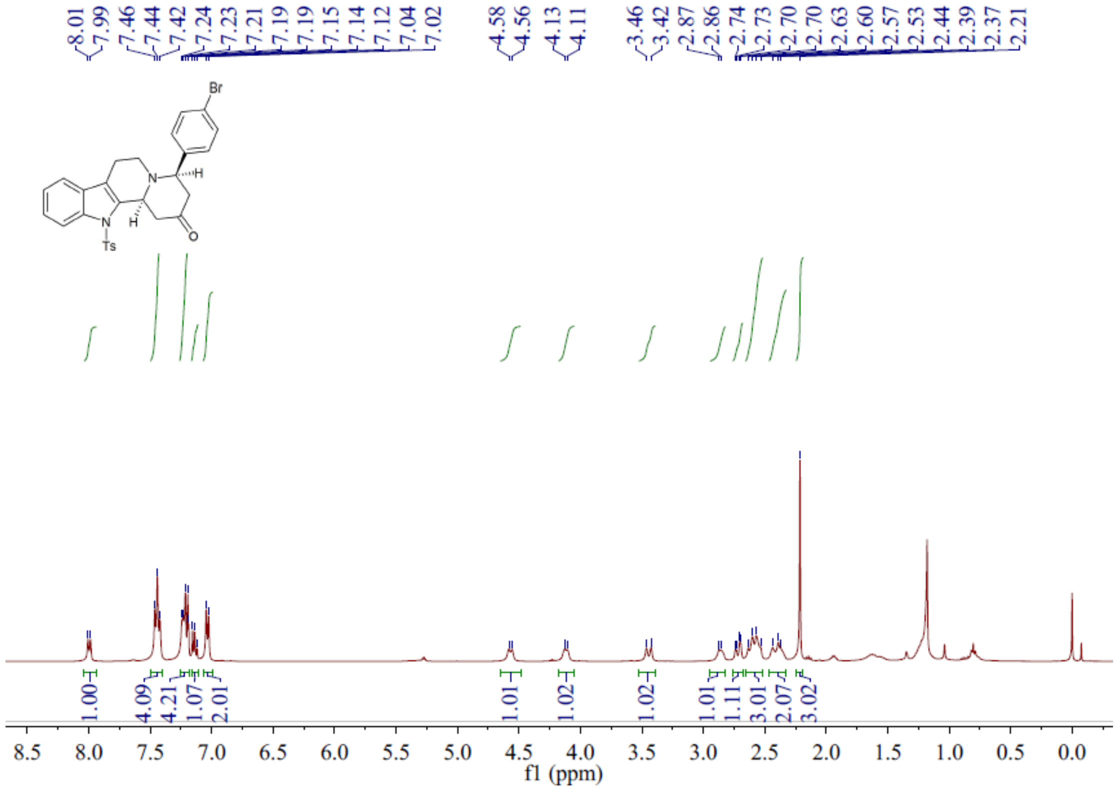

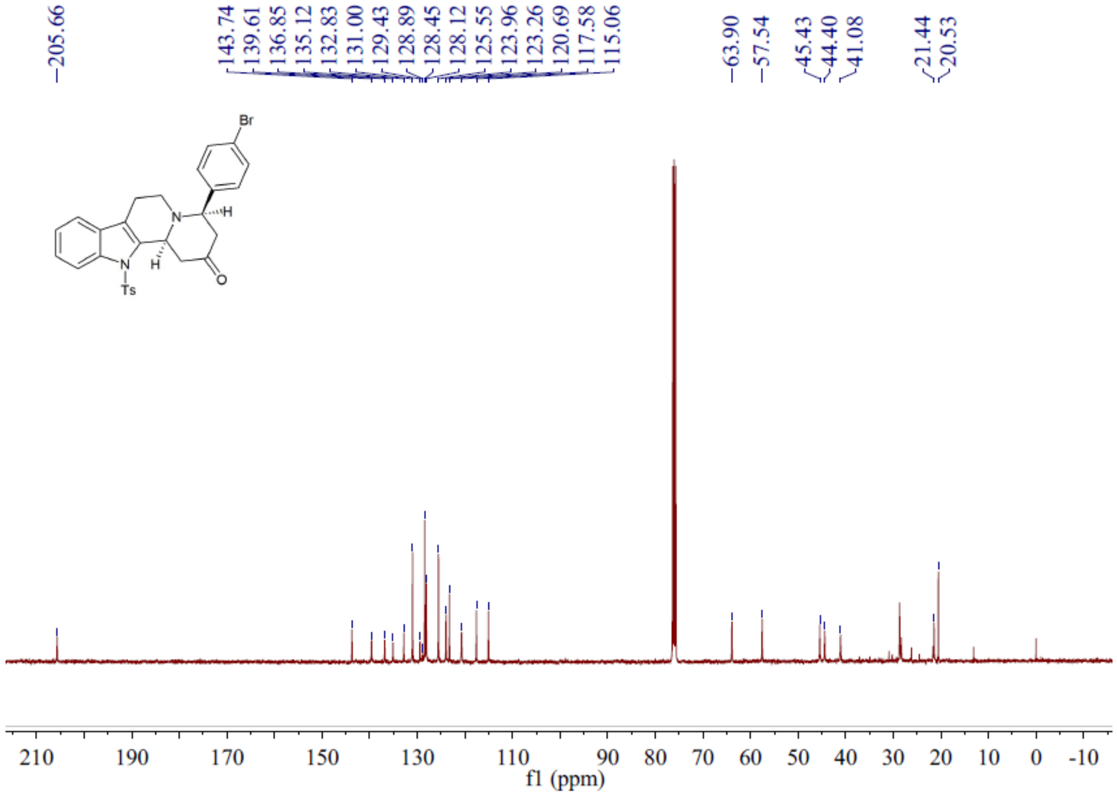

(4S,12bS)-4-(4-bromophenyl)-12-tosyl-1,3,4,6,7,12b-hexahydroindolo[2,3-a]quinolizin-2(12H)-one (7ag)

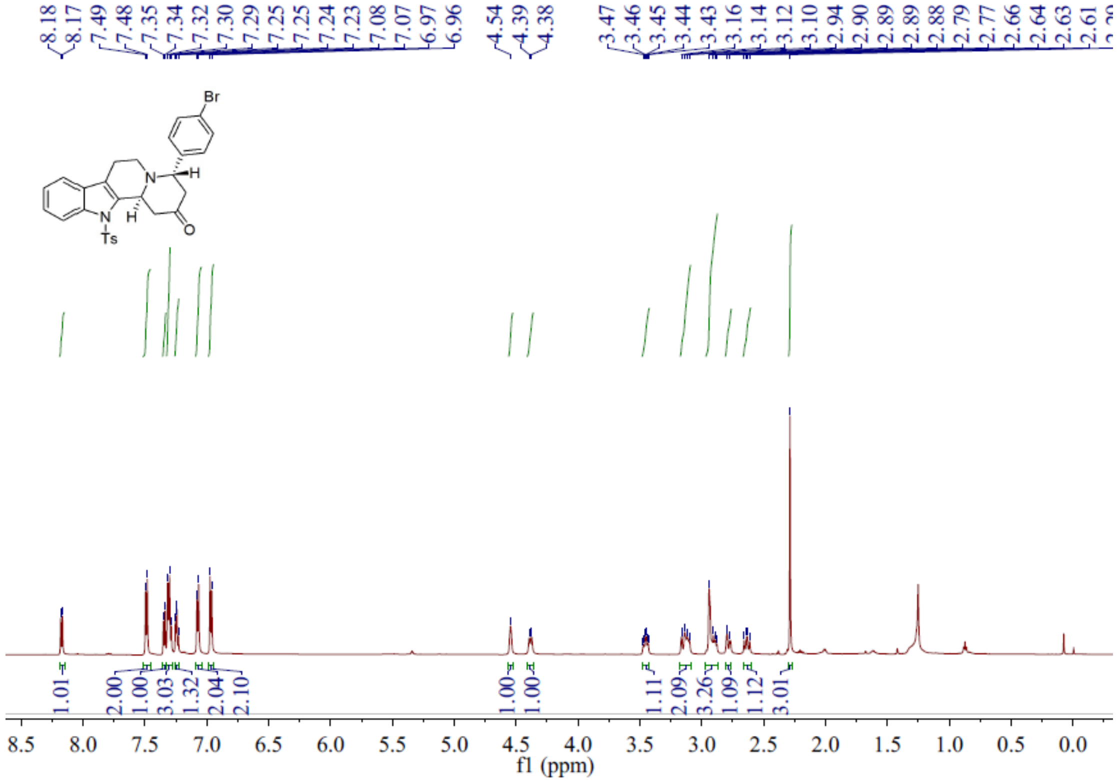

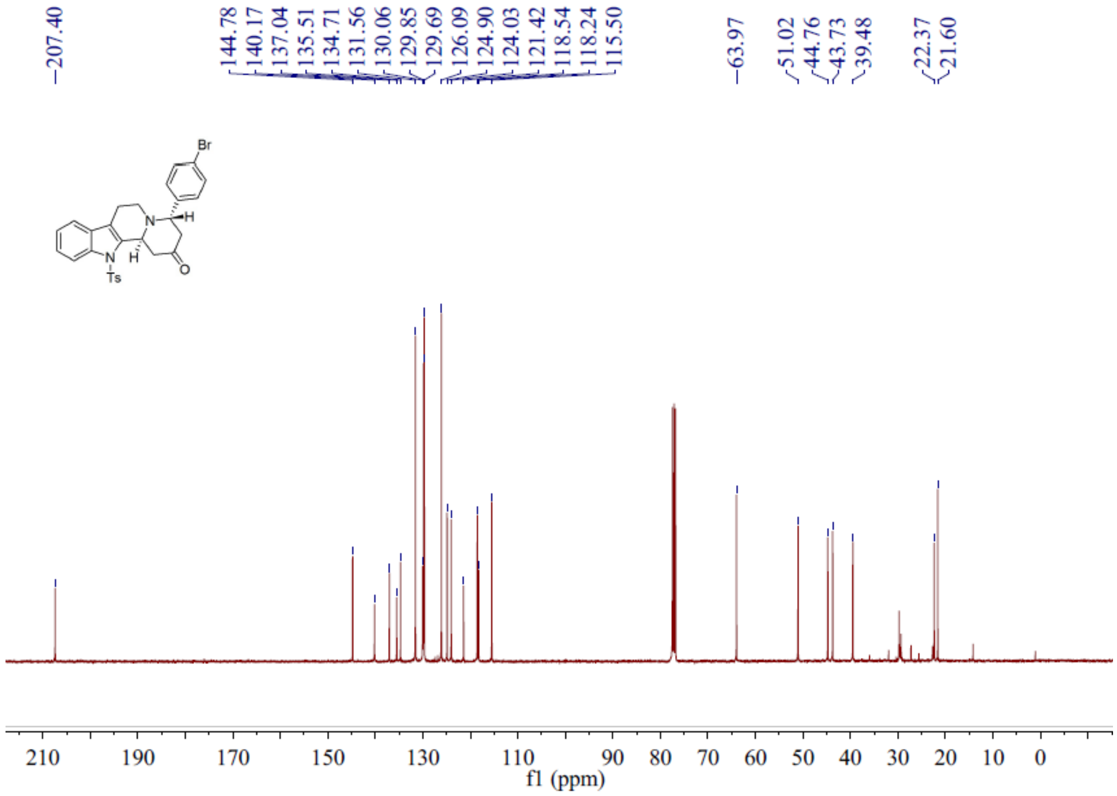

(4R,12bS)-12-tosyl-4-(4-(trifluoromethyl)phenyl)-1,3,4,6,7,12b-hexahydroindolo[2,3-a]quinolizin-2(12H)-one (6ah)

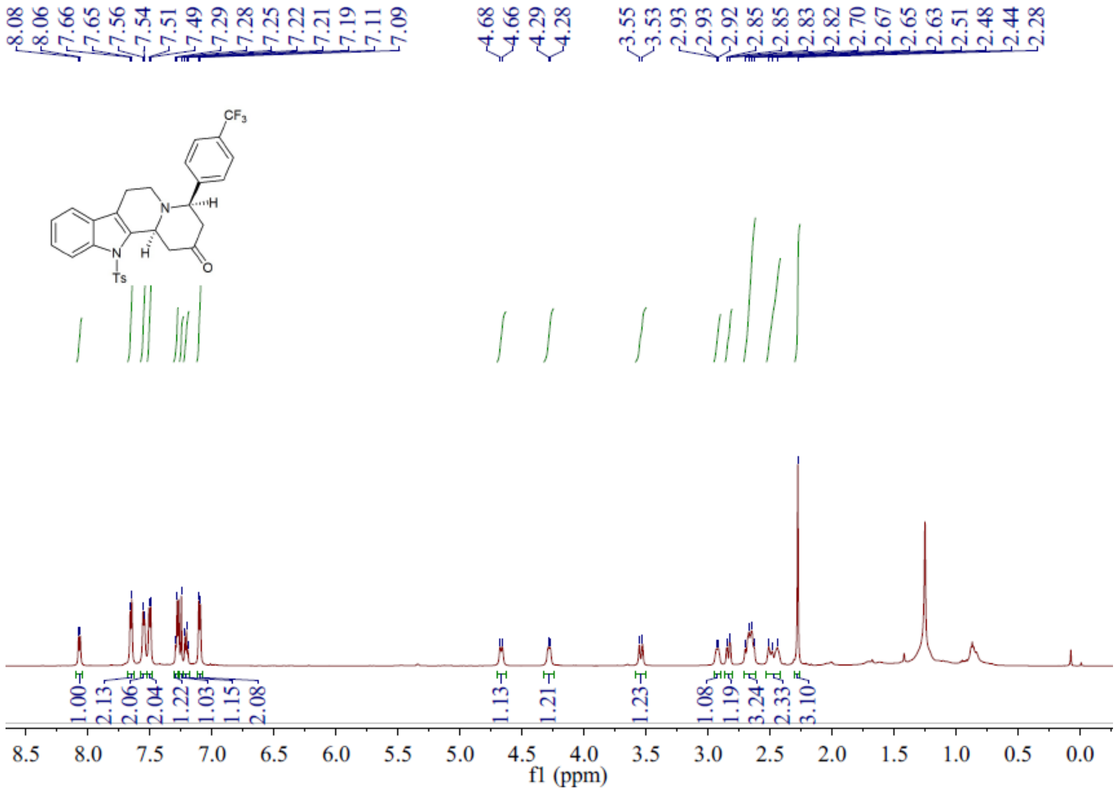

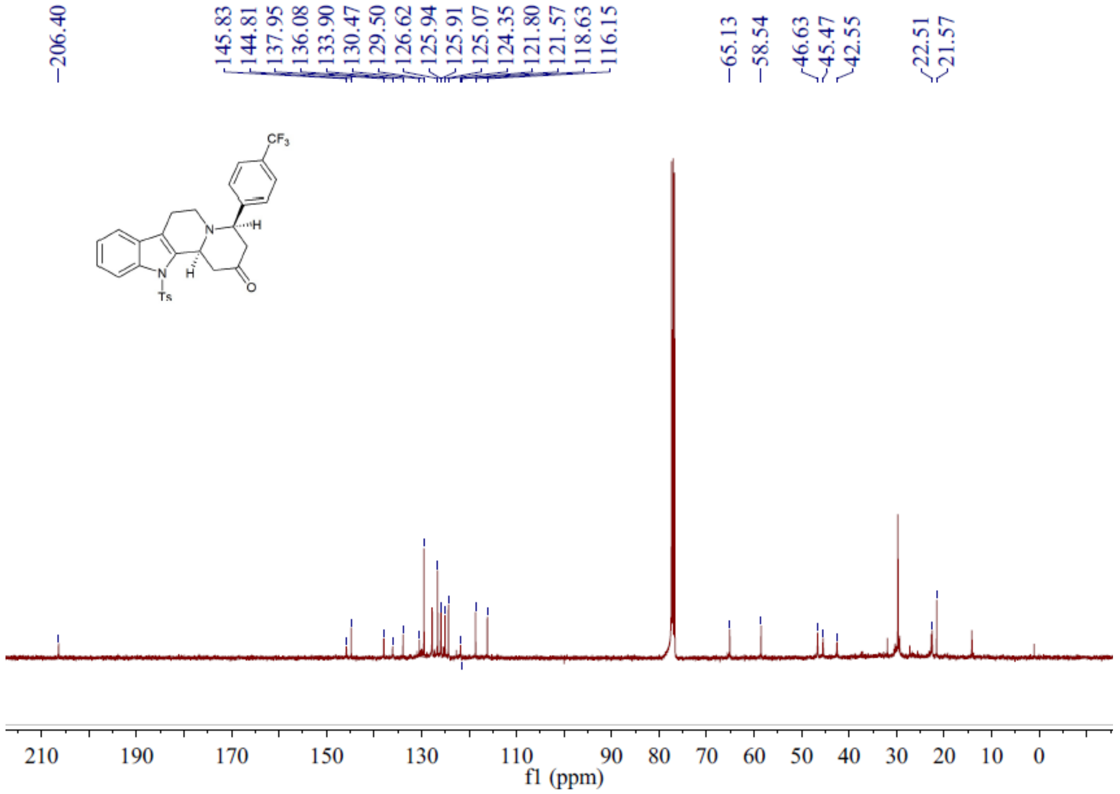

(4S,12bS)-12-tosyl-4-(4-(trifluoromethyl)phenyl)-1,3,4,6,7,12b-hexahydroindolo[2,3-a]quinolizin-2(12H)-one (7ah)

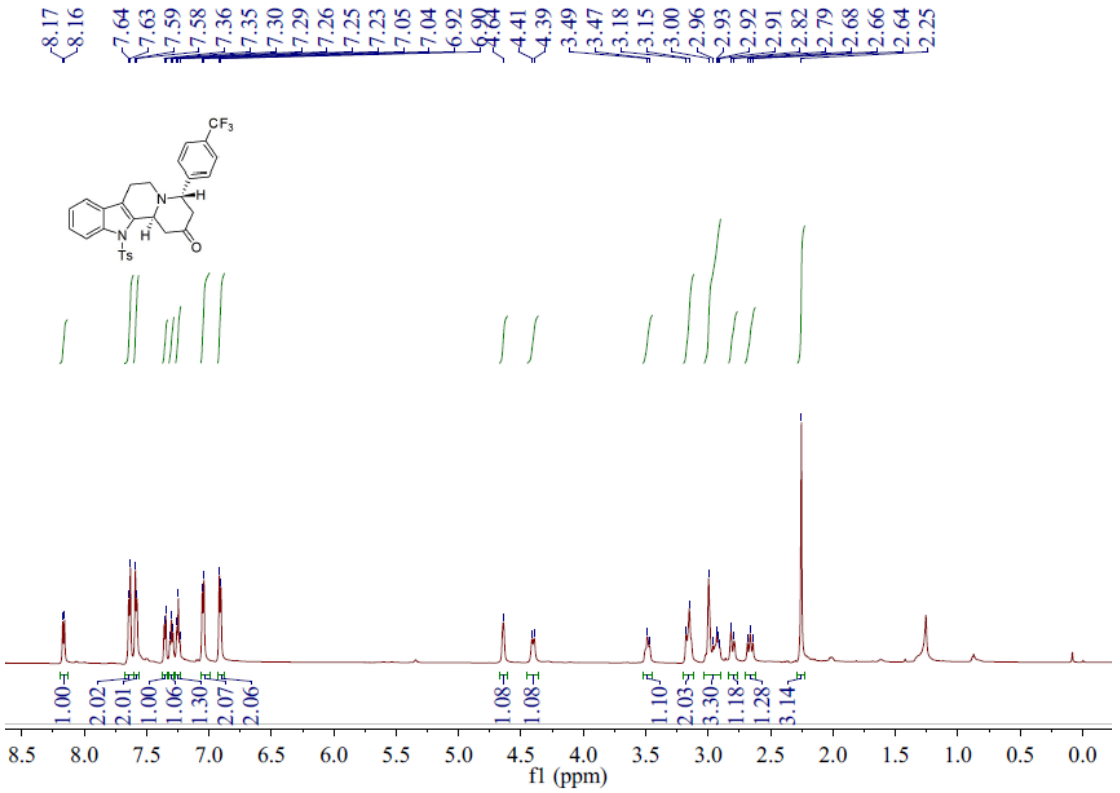

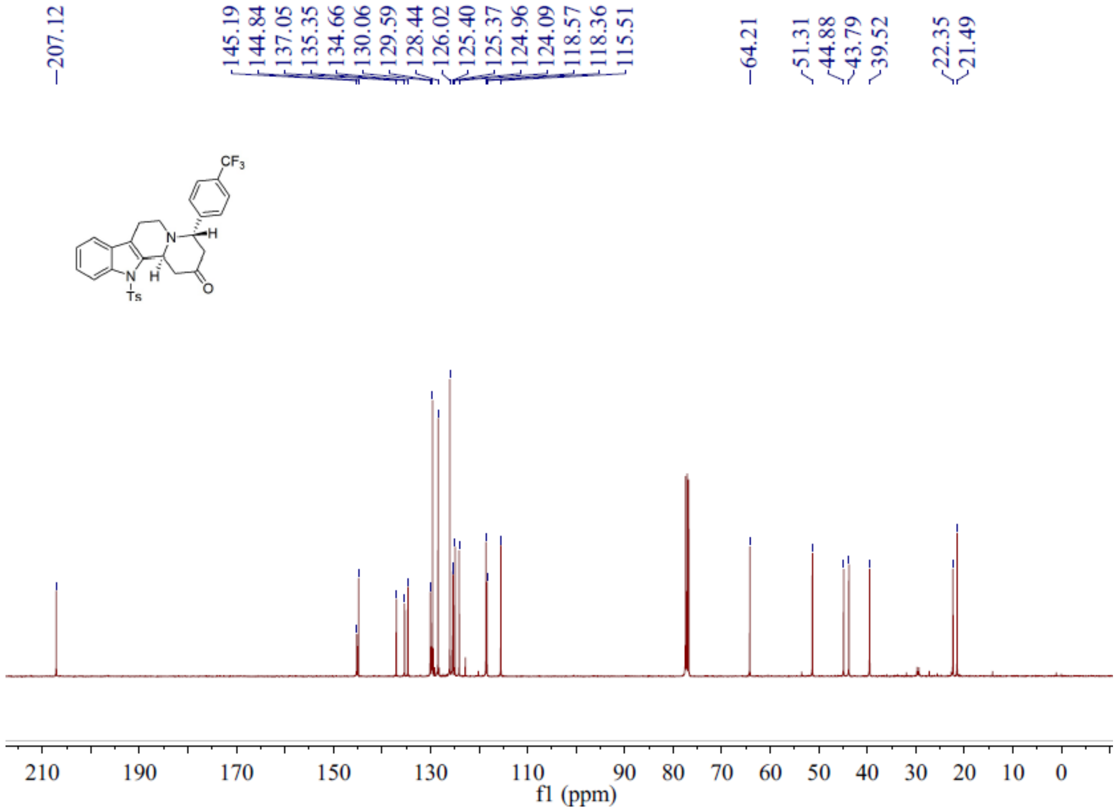

(4R,12bS)-4-(3-nitrophenyl)-12-tosyl-1,3,4,6,7,12b-hexahydroindolo[2,3-a]quinolizin-2(12H)-one (6ai)

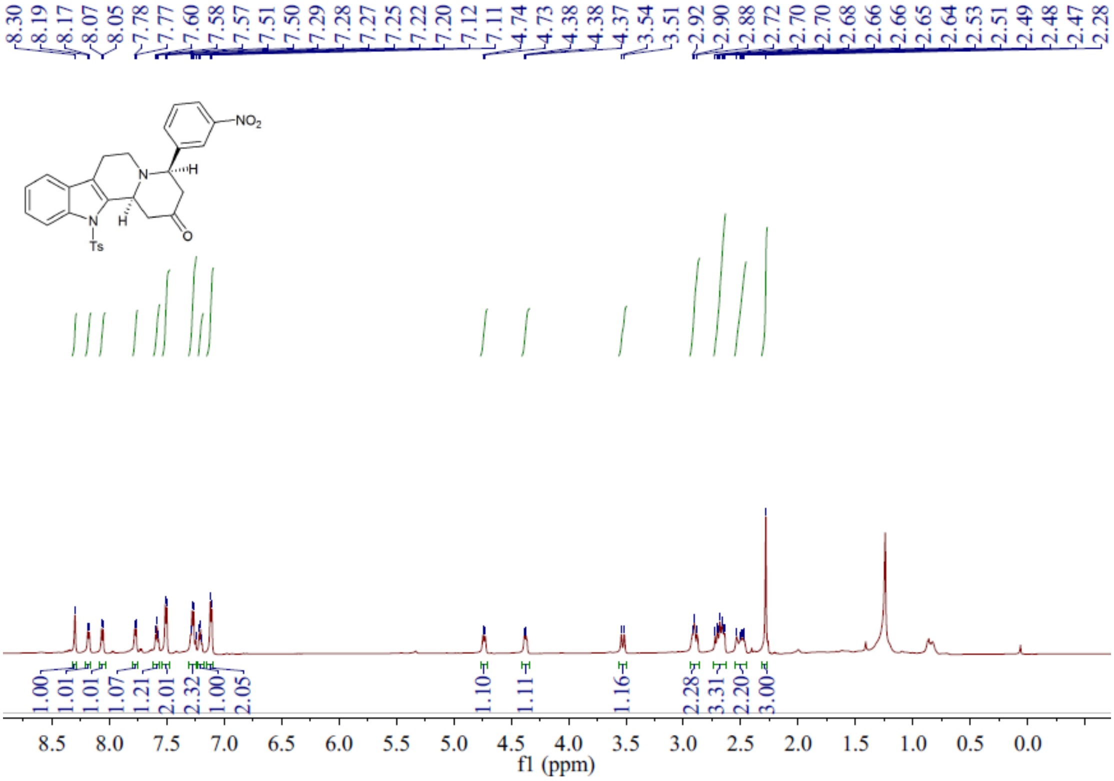

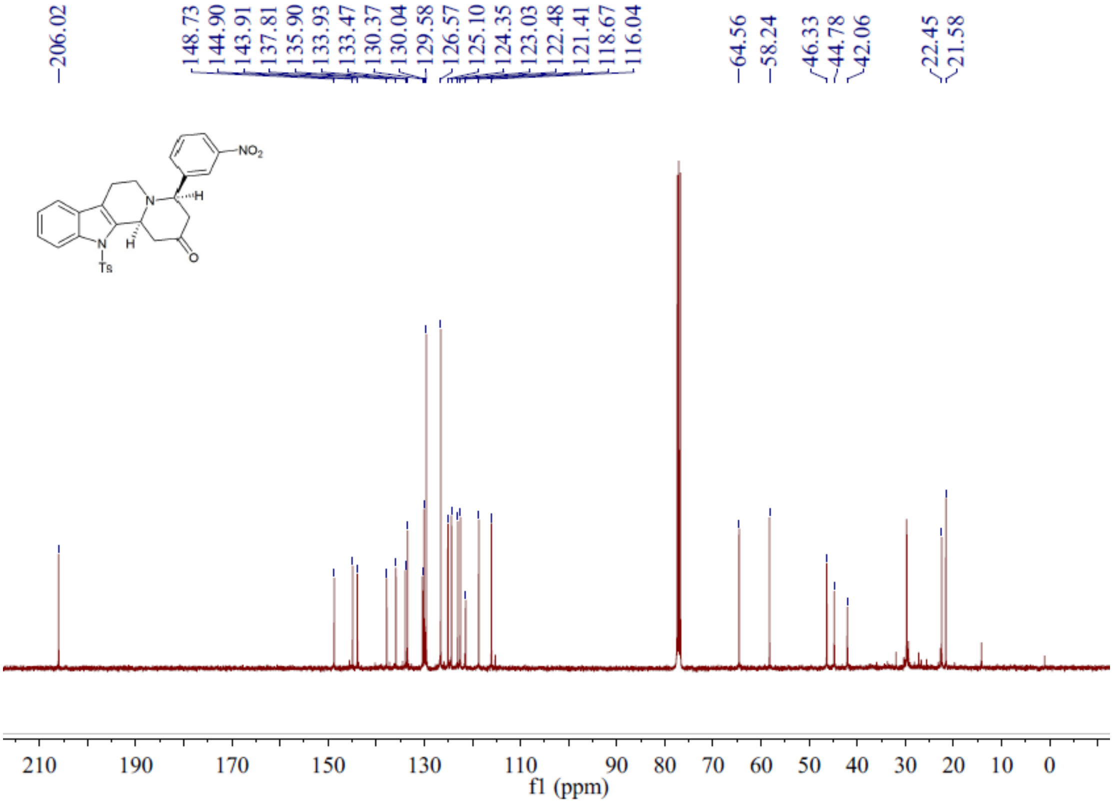

(4S,12bS)-4-(3-nitrophenyl)-12-tosyl-1,3,4,6,7,12b-hexahydroindolo[2,3-a]quinolizin-2(12H)-one (7ai)

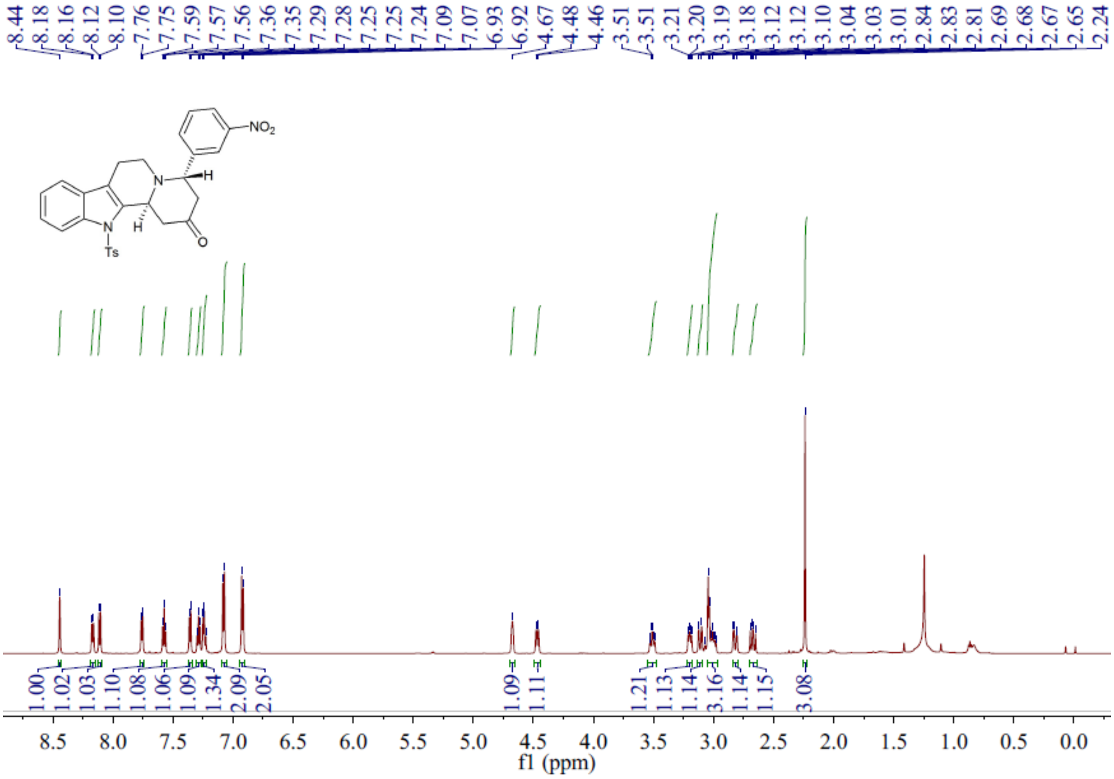

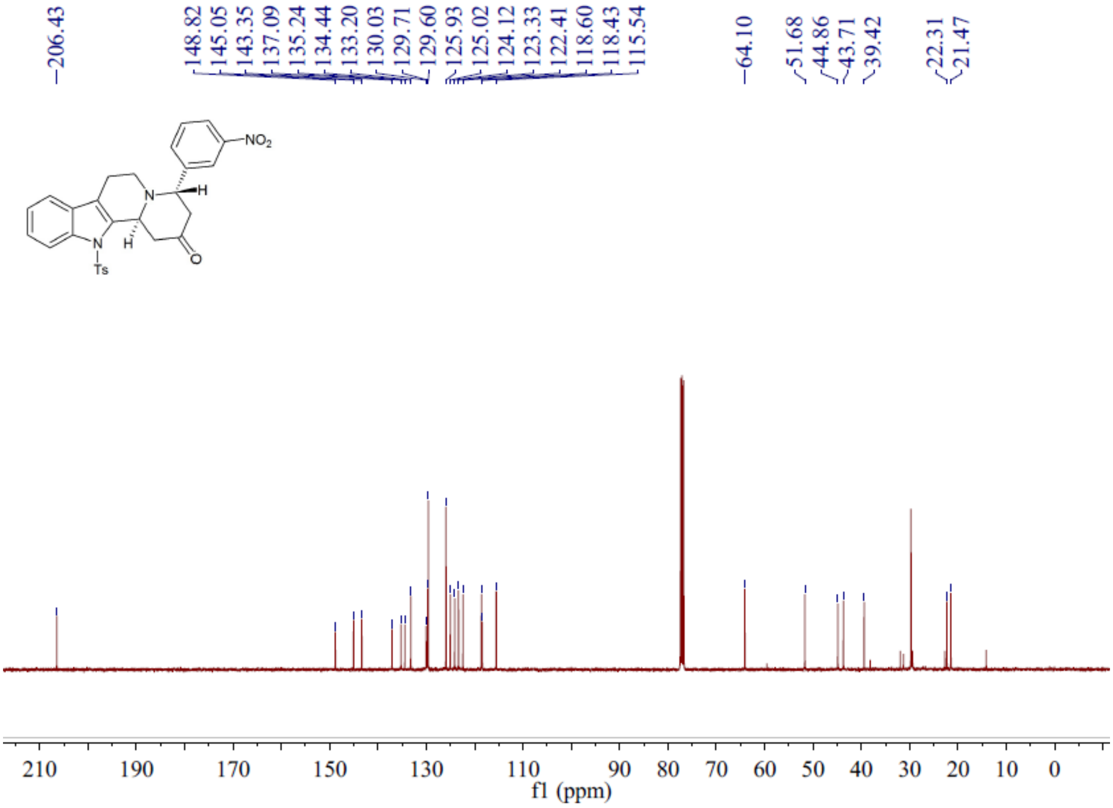

(4R,12bS)-4-(perfluorophenyl)-12-tosyl-1,3,4,6,7,12b-hexahydroindolo[2,3-a]quinolizin-2(12H)-one (6aj)

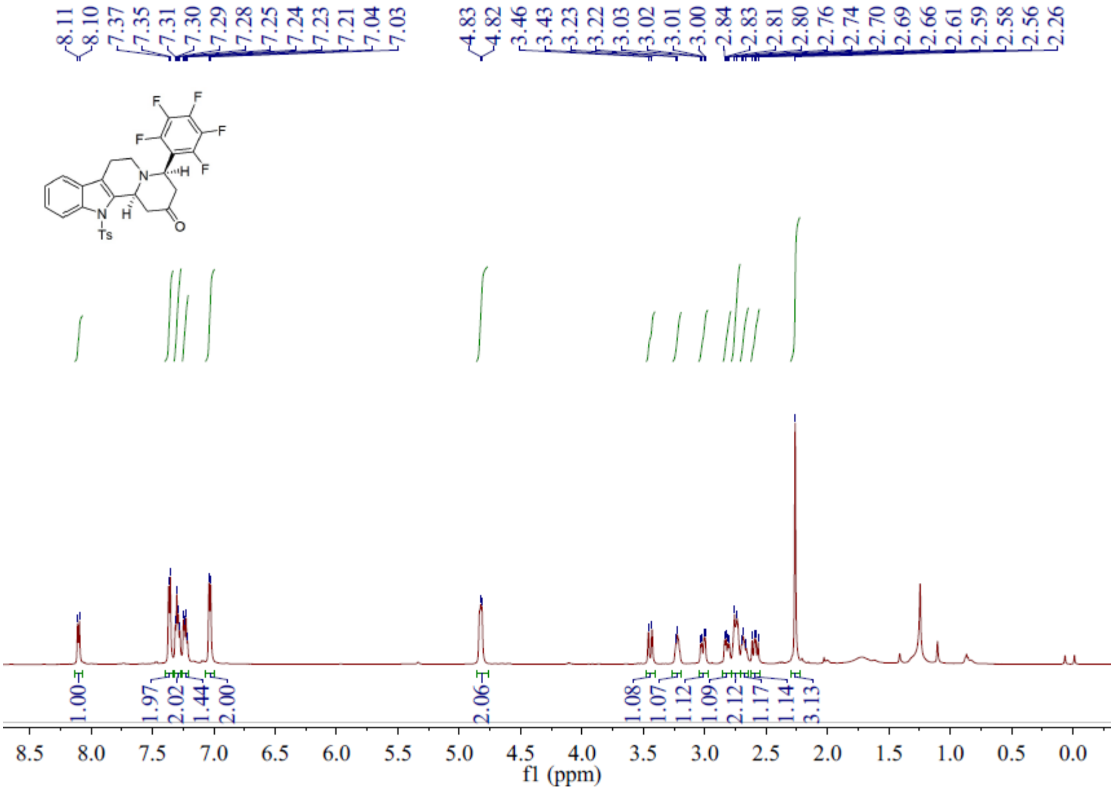

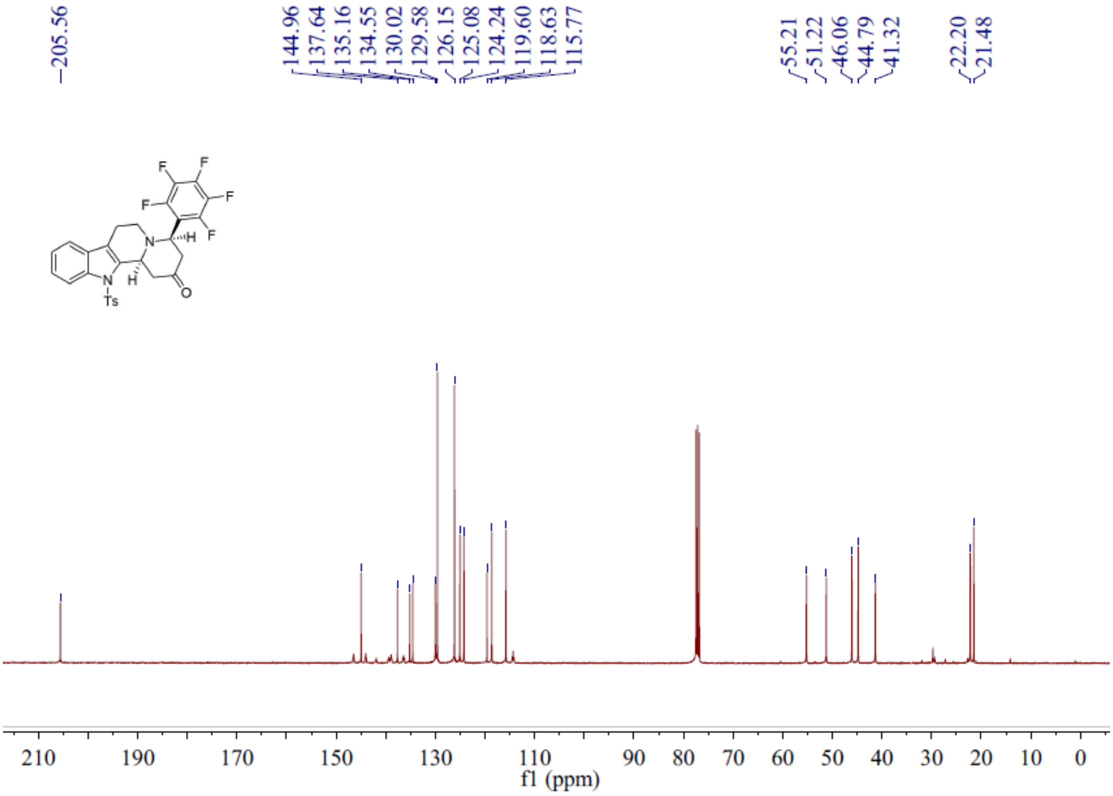

(4R,12bS)-4-(2,3-dichlorophenyl)-12-tosyl-1,3,4,6,7,12b-hexahydroindolo[2,3-a]quinolizin-2(12H)-one (6ak)

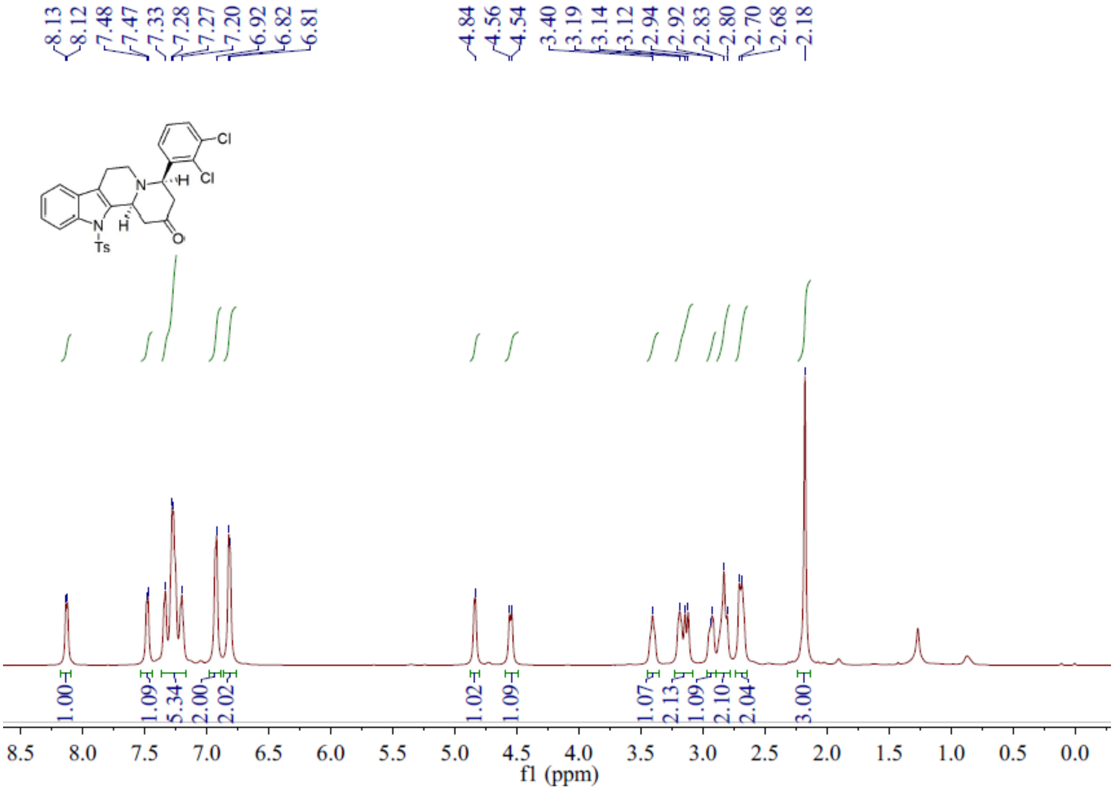

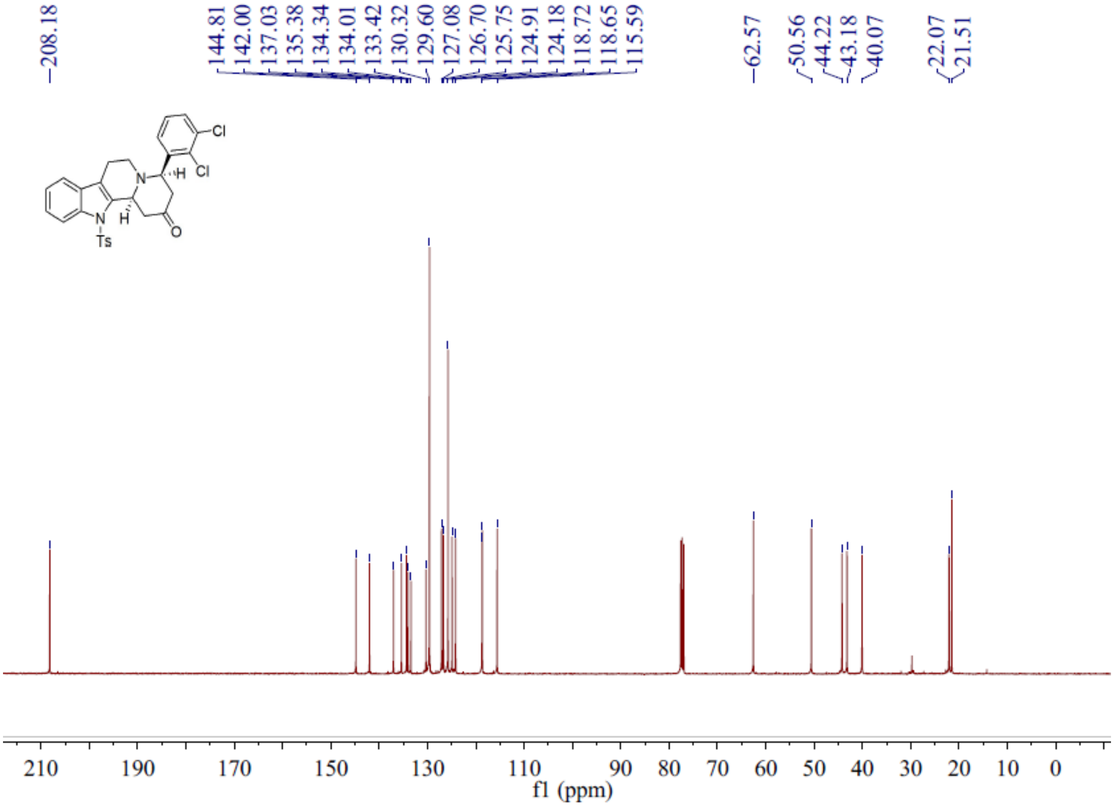

(4R,12bS)-4-(2,4-dinitrophenyl)-12-tosyl-1,3,4,6,7,12b-hexahydroindolo[2,3-a]quinolizin-2(12H)-one (6al)

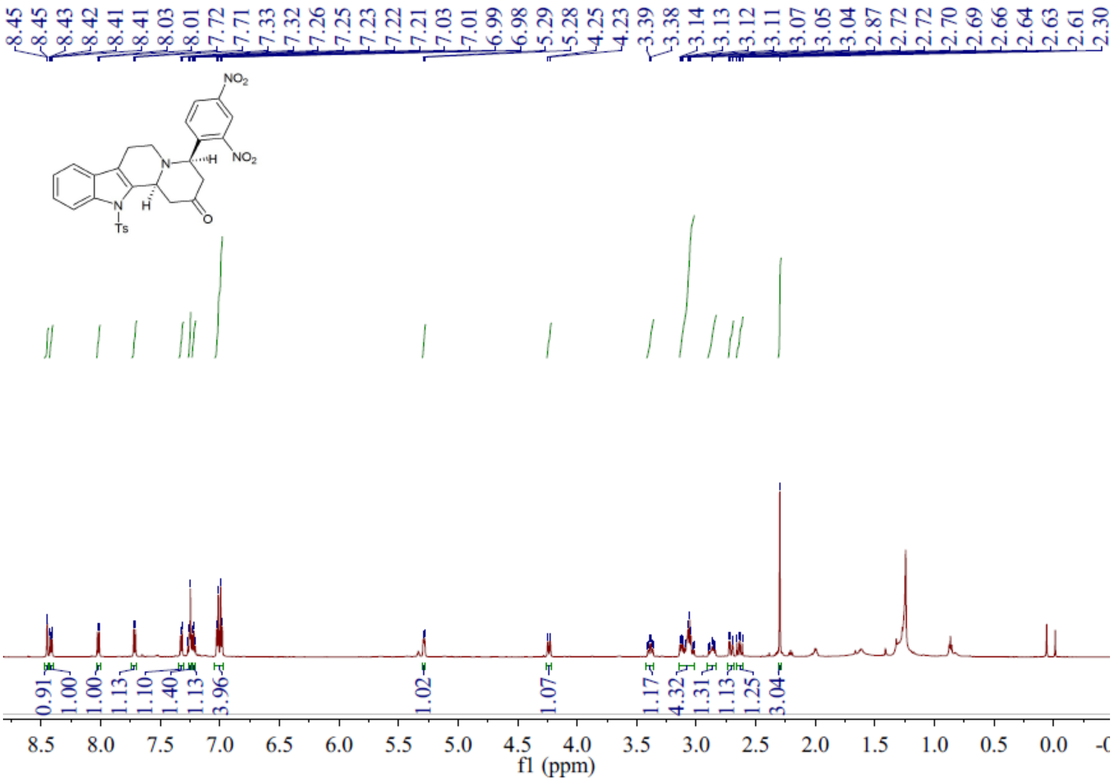

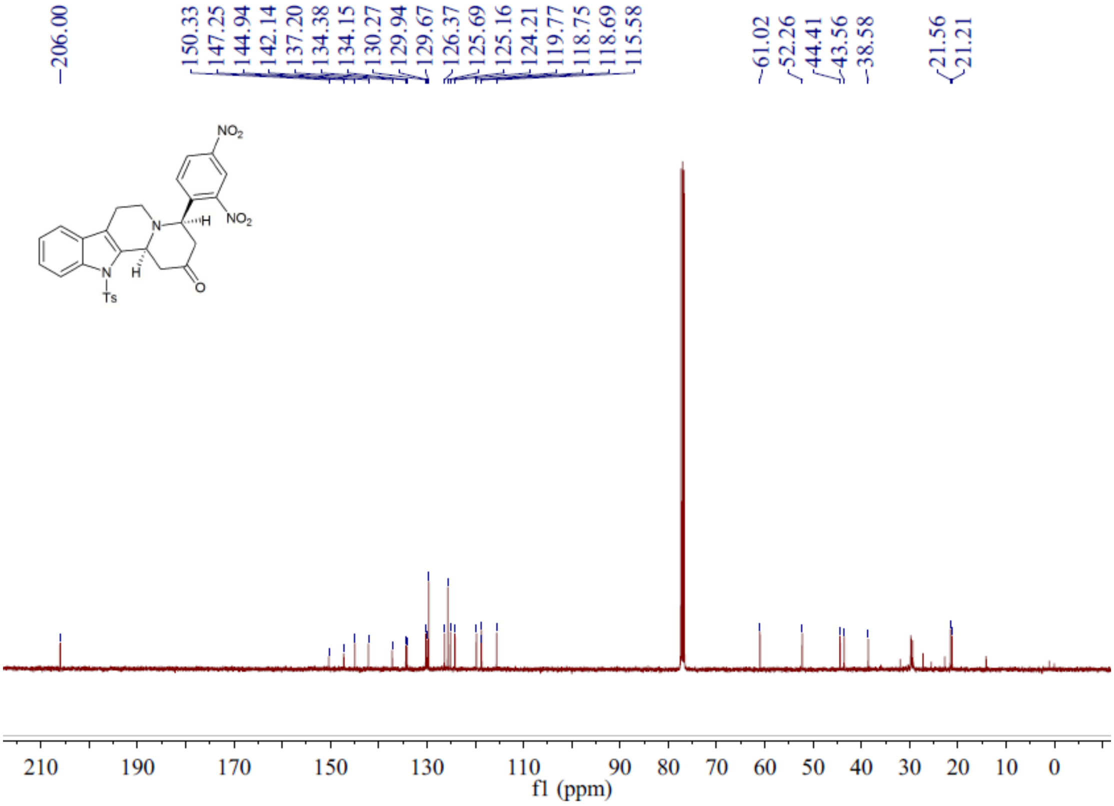

(4R,12bS)-4-(thiophen-2-yl)-12-tosyl-1,3,4,6,7,12b-hexahydroindolo[2,3-a]quinolizin-2(12H)-one (6am)

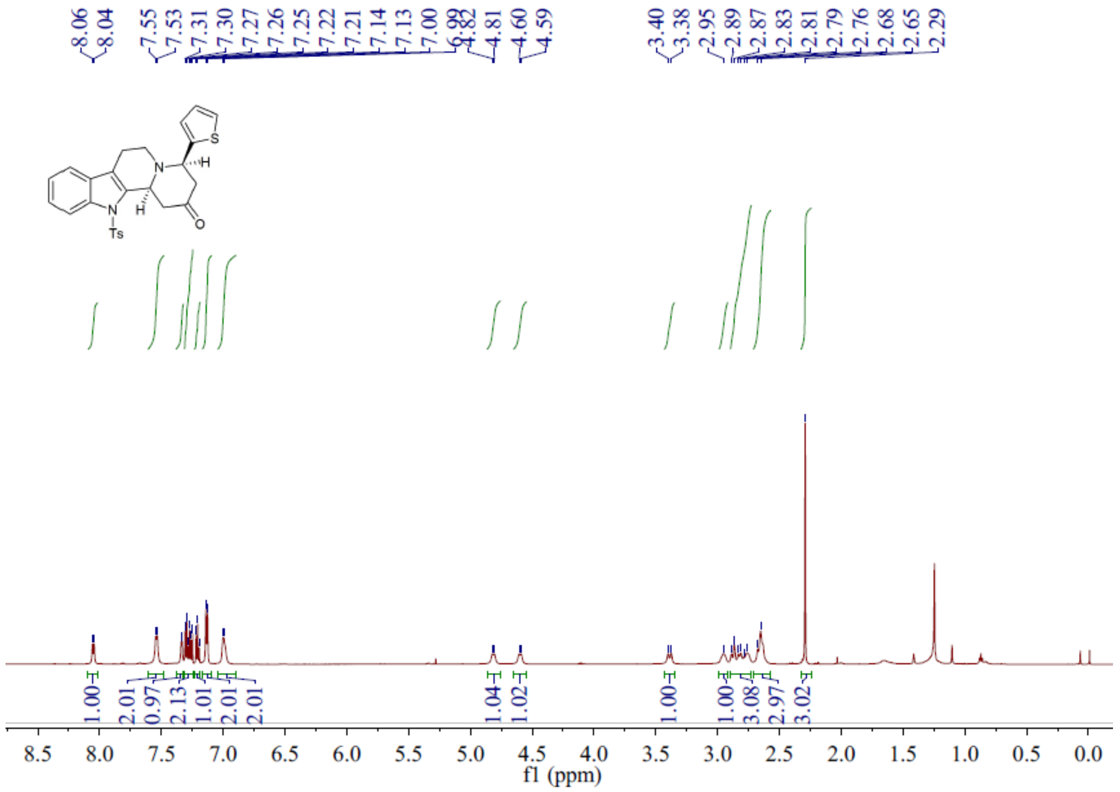

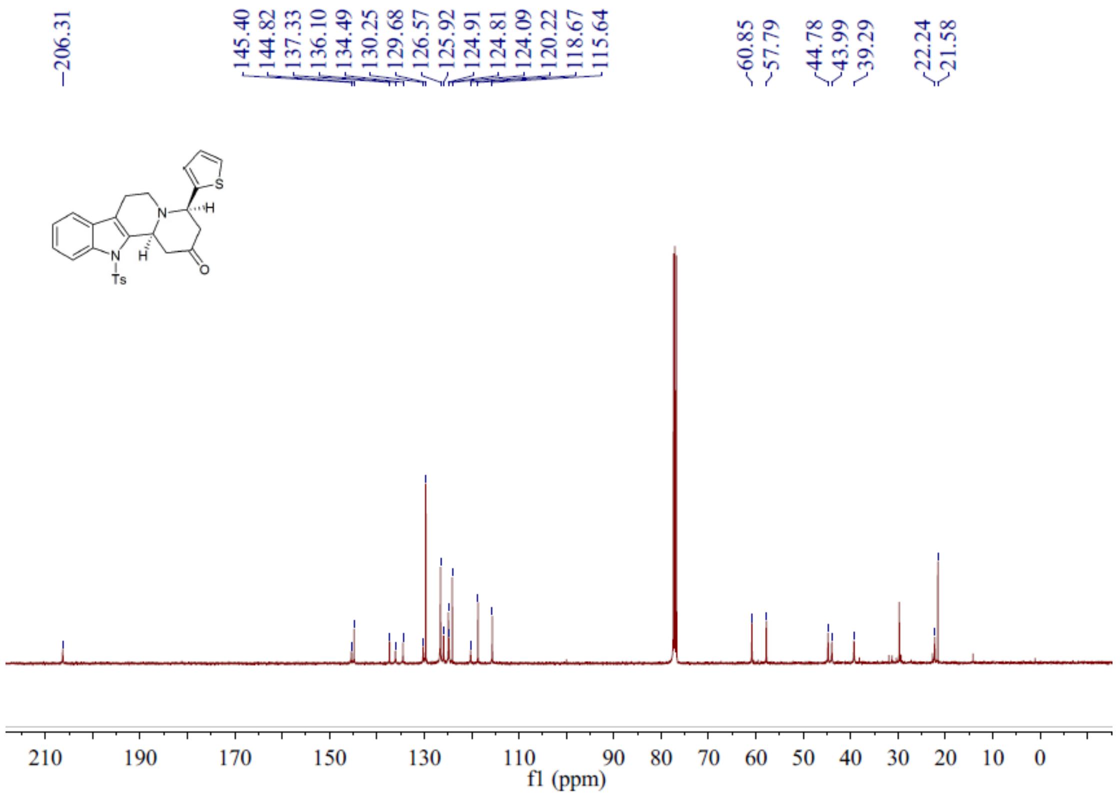

(4S,12bS)-4-(furan-2-yl)-12-tosyl-1,3,4,6,7,12b-hexahydroindolo[2,3-a]quinolizin-2(12H)-one (7an)

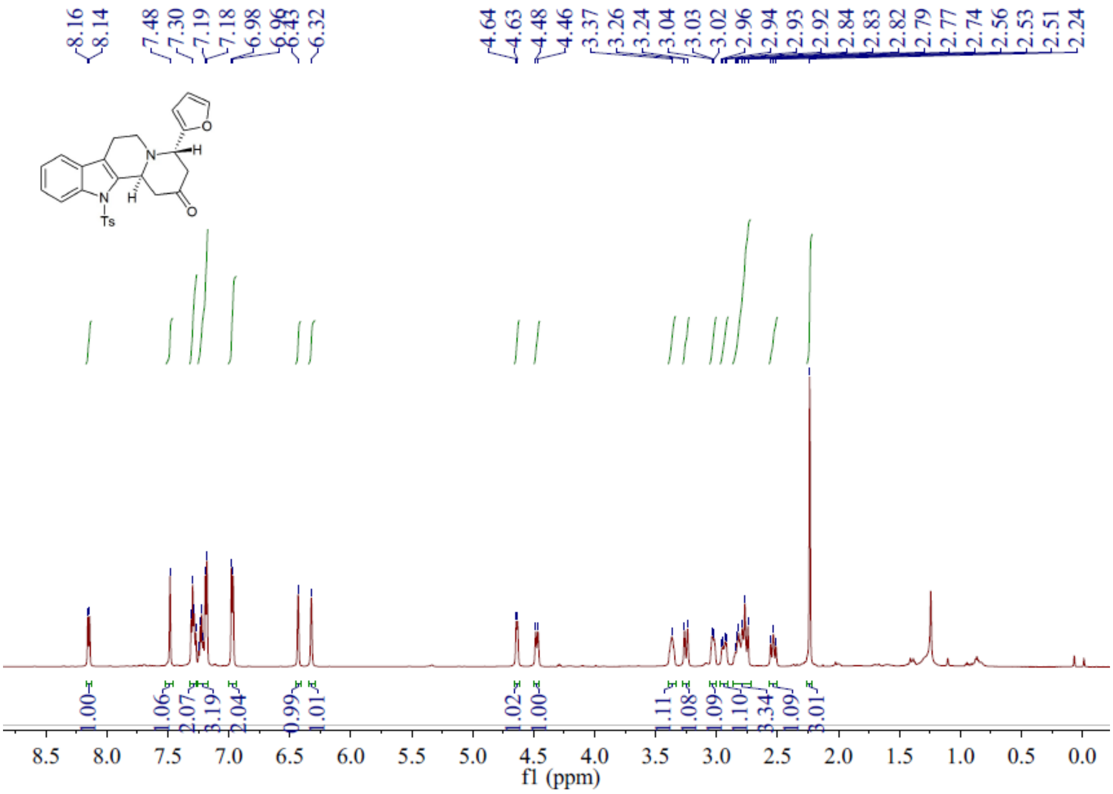

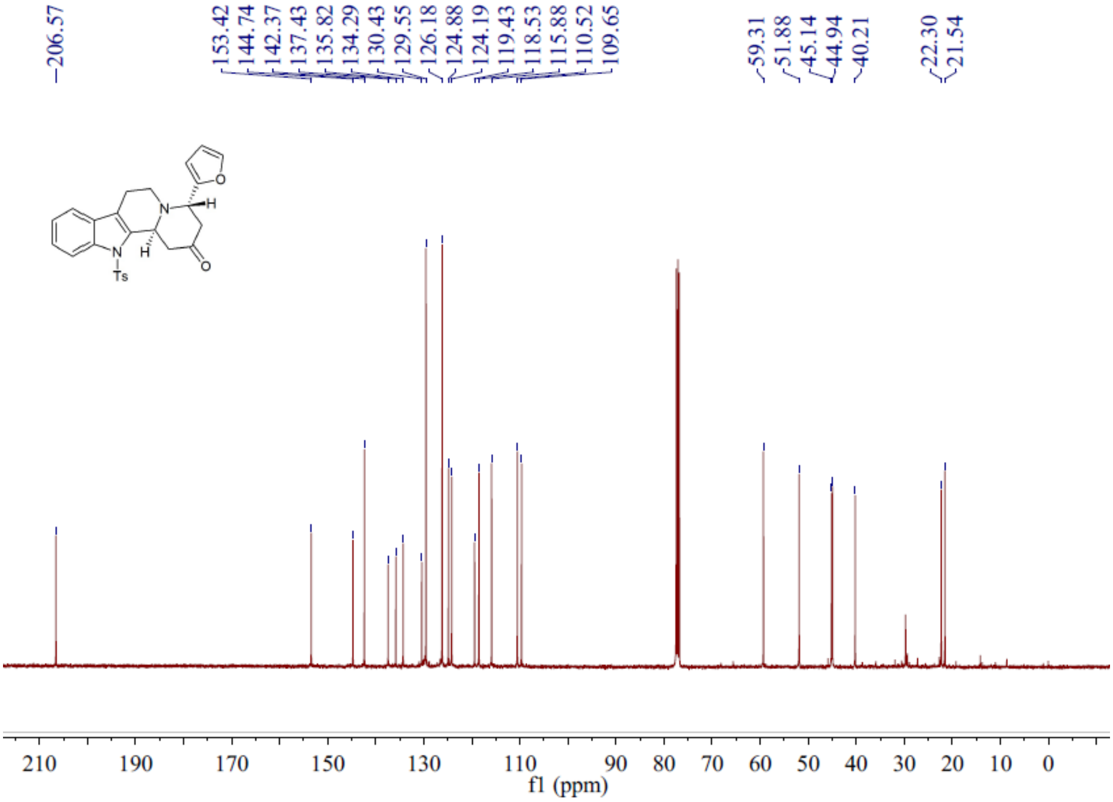

(4R,12bS)-4-(2-methylprop-1-enyl)-12-tosyl-1,3,4,6,7,12b-hexahydroindolo[2,3-a]quinolizin-2(12H)-one (6ao)

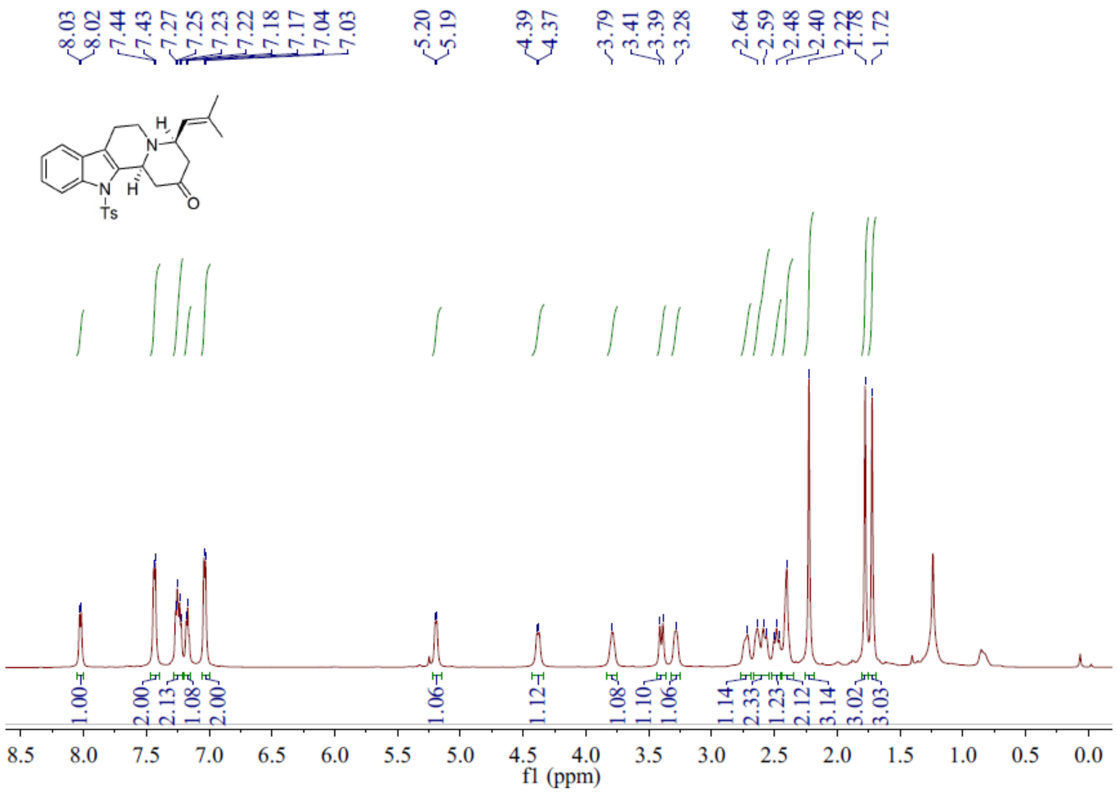

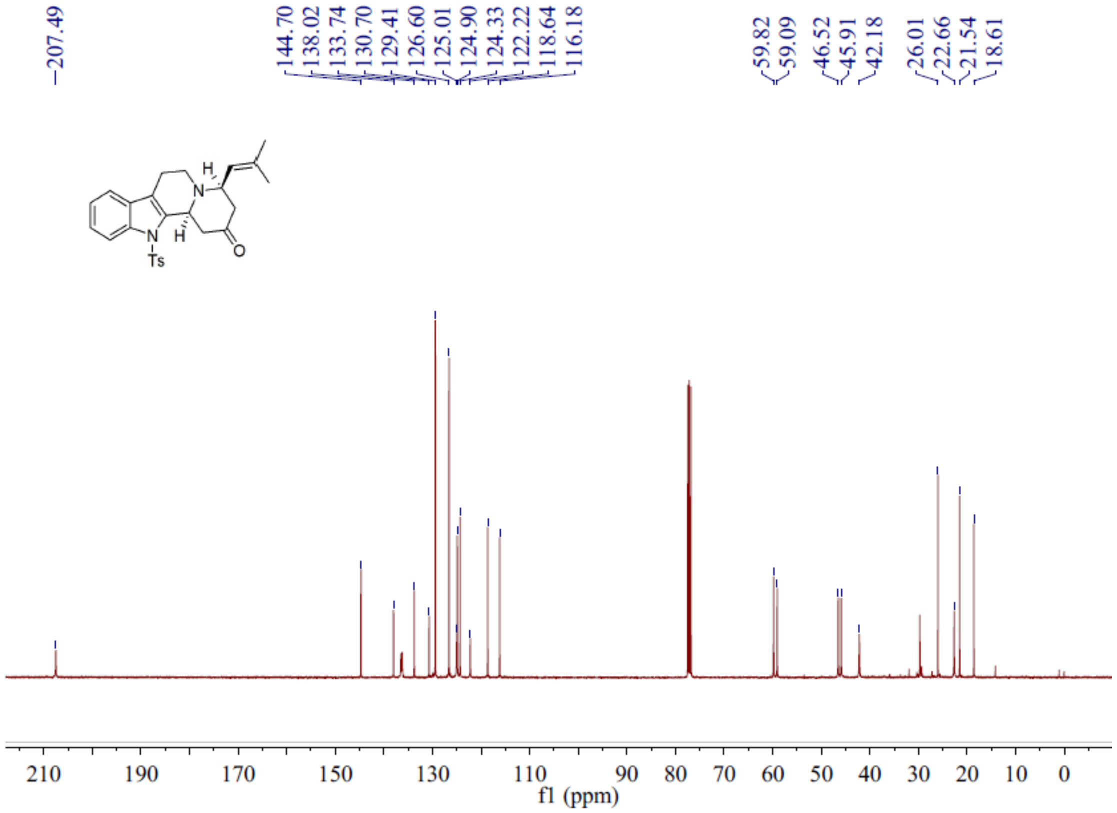

7. HPLC Analysis for Products

(4R,12bS)-4-(4-methoxy-2-methylphenyl)-12-tosyl-1,3,4,6,7,12b-hexahydroindolo[2,3-a]quinolizin-2(12H)-one (6aa)

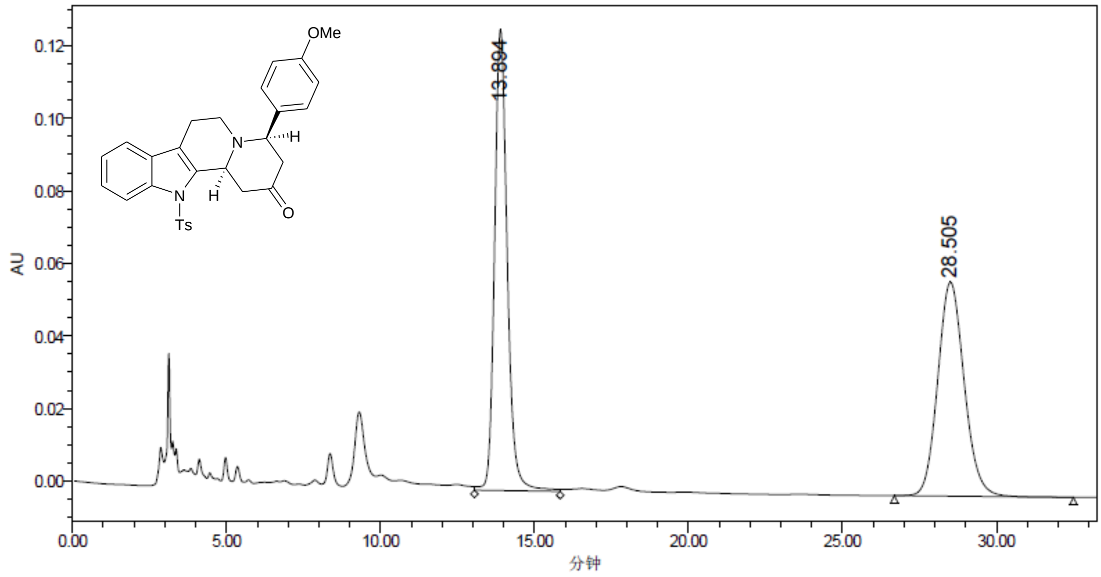

	RT (min)	Area (磺*sec)	% Area	Height (磺)	% Height
1	13.894	3642844	50.82	127182	68.29
2	28.505	3524769	49.18	59051	31.71

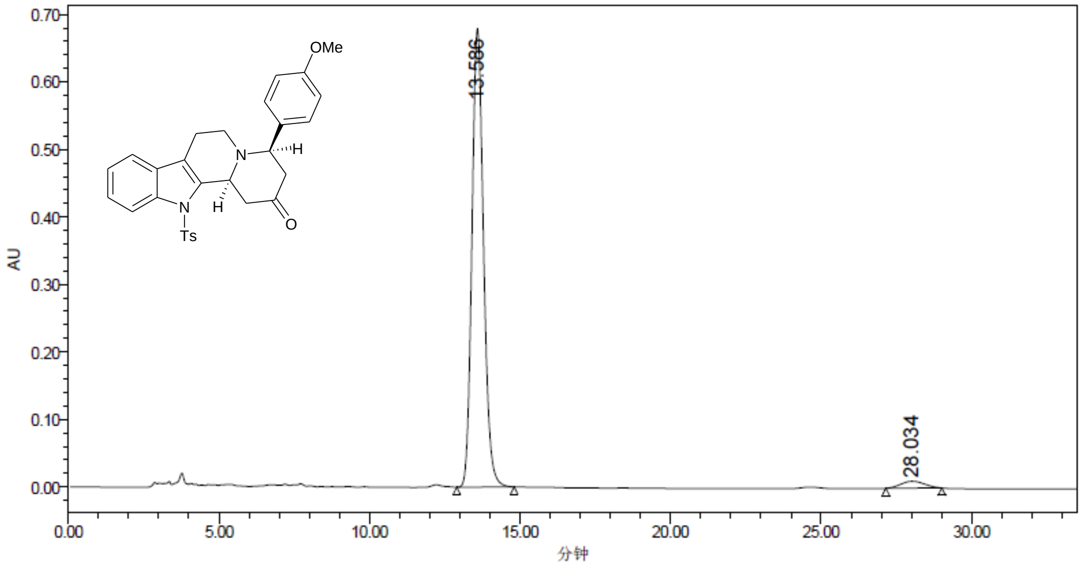

	RT (min)	Area (磺*sec)	% Area	Height (磺)	% Height
1	13.586	18161529	97.03	678618	98.52
2	28.034	556071	2.97	10192	1.48

(4S,12bS)-4-(4-methoxyphenyl)-12-tosyl-1,3,4,6,7,12b-hexahydroindolo[2,3-a]quinolizin-2(12H)-one (7aa)

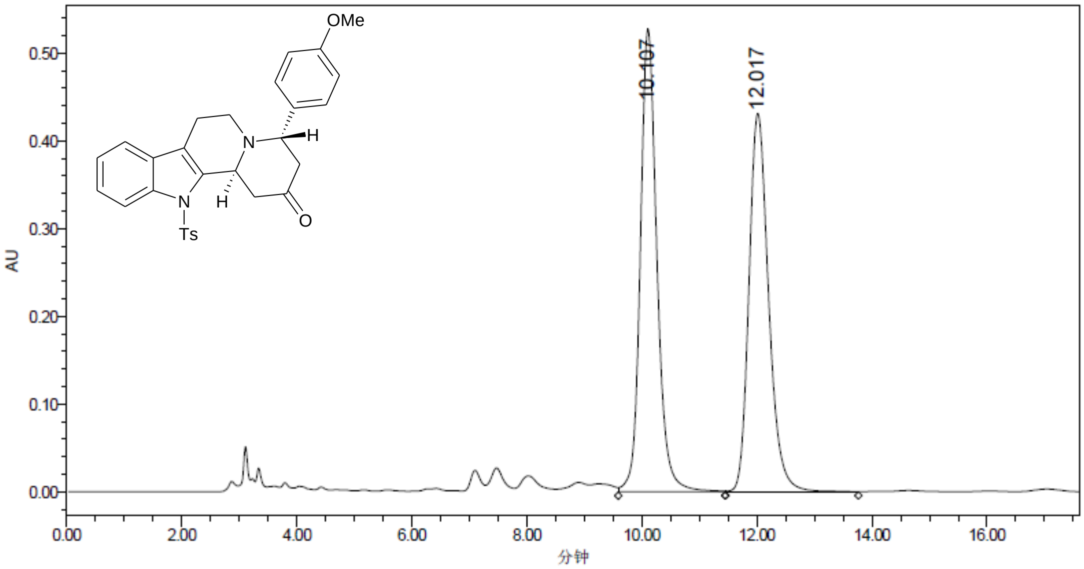

	RT (min)	Area (磺*sec)	% Area	Height (磺)	% Height
1	10.107	10596536	50.82	527347	55.00
2	12.017	10252604	49.18	431519	45.00

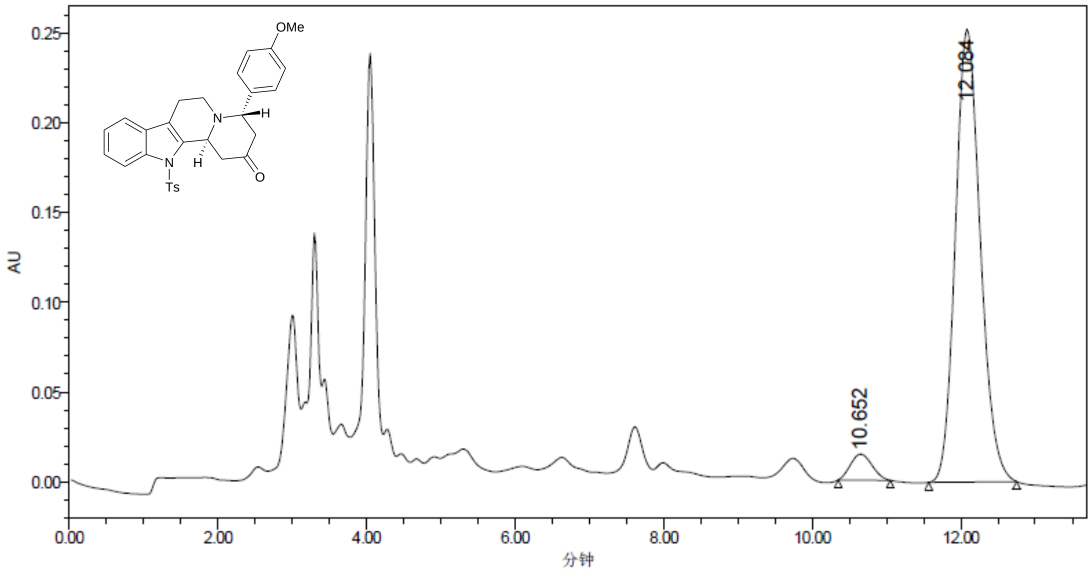

	RT (min)	Area (磺*sec)	% Area	Height (磺)	% Height
1	10.652	293405	4.76	14551	5.46
2	12.084	5874584	95.24	251924	94.54

(4R,12bS)-4-phenyl-12-tosyl-1,3,4,6,7,12b-hexahydroindolo[2,3-a]quinolizin-2(12H)-one (6ab)

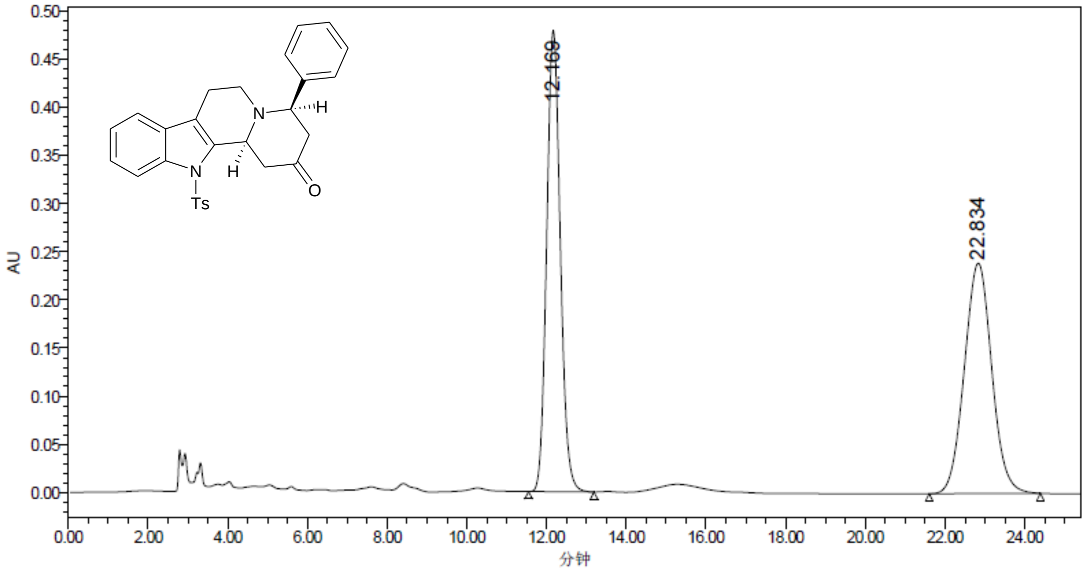

	RT (min)	Area (礦*sec)	% Area	Height (礦)	% Height
1	12.169	11387974	49.90	478778	66.67
2	22.834	11435492	50.10	239343	33.33

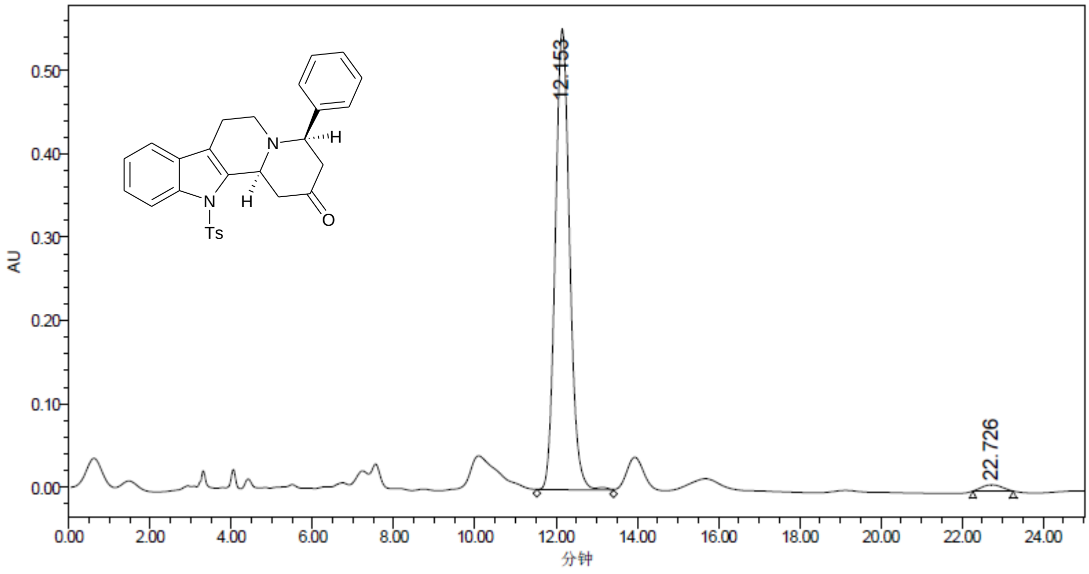

	RT (min)	Area (礦*sec)	% Area	Height (礦)	% Height
1	12.153	13583888	98.15	552406	98.66
2	22.726	255998	1.85	7497	1.34

(4S,12bS)-4-phenyl-12-tosyl-1,3,4,6,7,12b-hexahydroindolo[2,3-a]quinolizin-2(12H)-one (7ab)

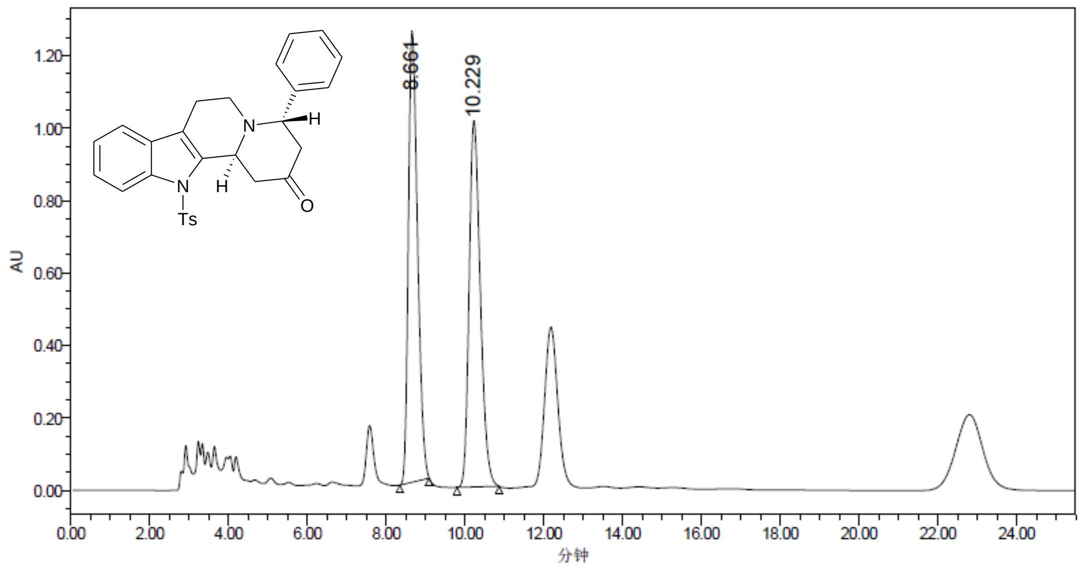

	RT (min)	Area (磺*sec)	% Area	Height (磺)	% Height
1	8.661	20074362	50.20	1244838	55.19
2	10.229	19912491	49.80	1010634	44.81

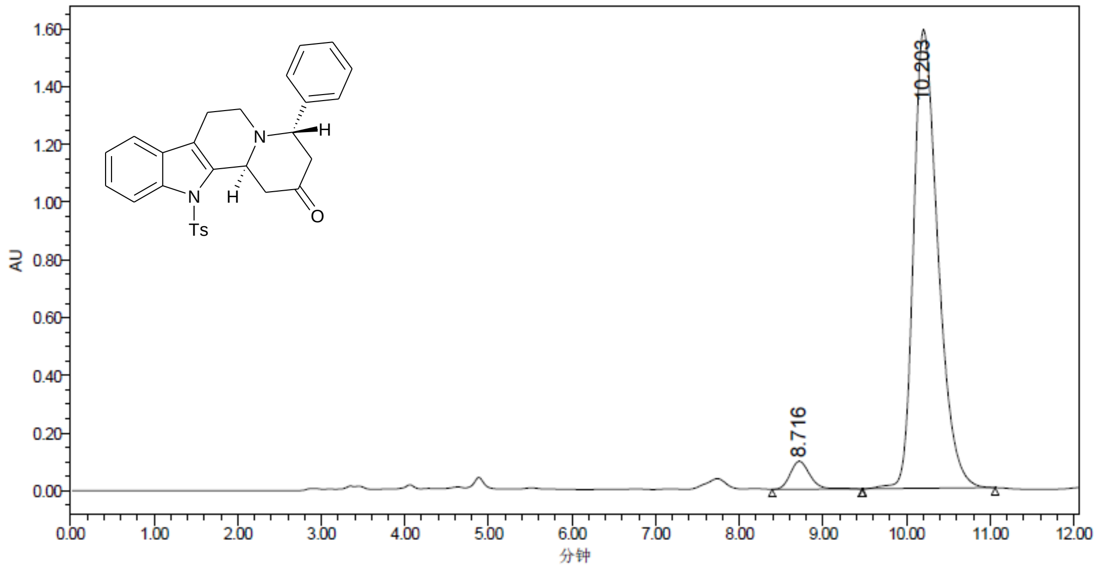

	RT (min)	Area (磺*sec)	% Area	Height (磺)	% Height
1	8.716	1572979	4.65	96349	5.72
2	10.203	32232470	95.35	1588796	94.28

(4R,12bS)-4-o-tolyl-12-tosyl-1,3,4,6,7,12b-hexahydroindolo[2,3-a]quinolizin-2(12H)-one (6ac)

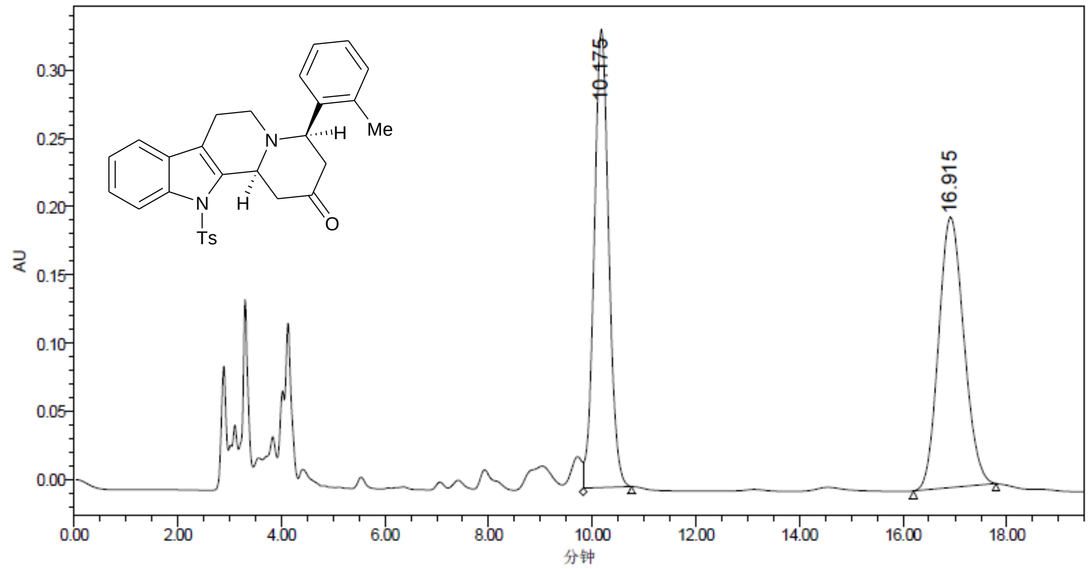

	RT (min)	Area (磺*sec)	% Area	Height (磺)	% Height
1	10.175	6537485	49.33	335202	62.87
2	16.915	6714304	50.67	197990	37.13

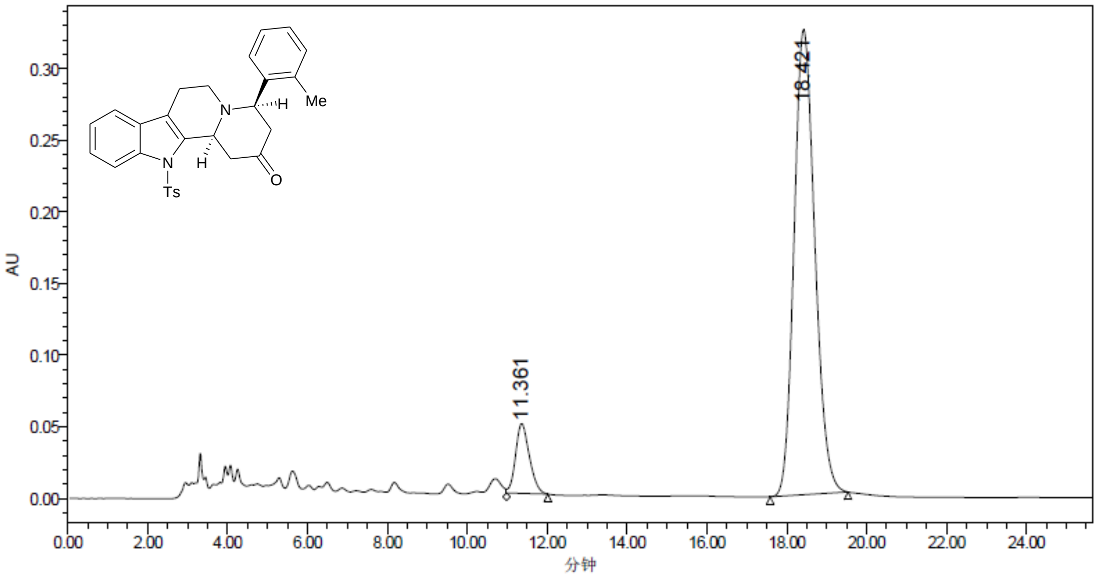

	RT (min)	Area (磺*sec)	% Area	Height (磺)	% Height
1	11.361	1172439	9.09	48688	13.05
2	18.421	11732088	90.91	324503	86.95

(4S,12bS)-4-o-tolyl-12-tosyl-1,3,4,6,7,12b-hexahydroindolo[2,3-a]quinolizin-2(12H)-one (7ac)

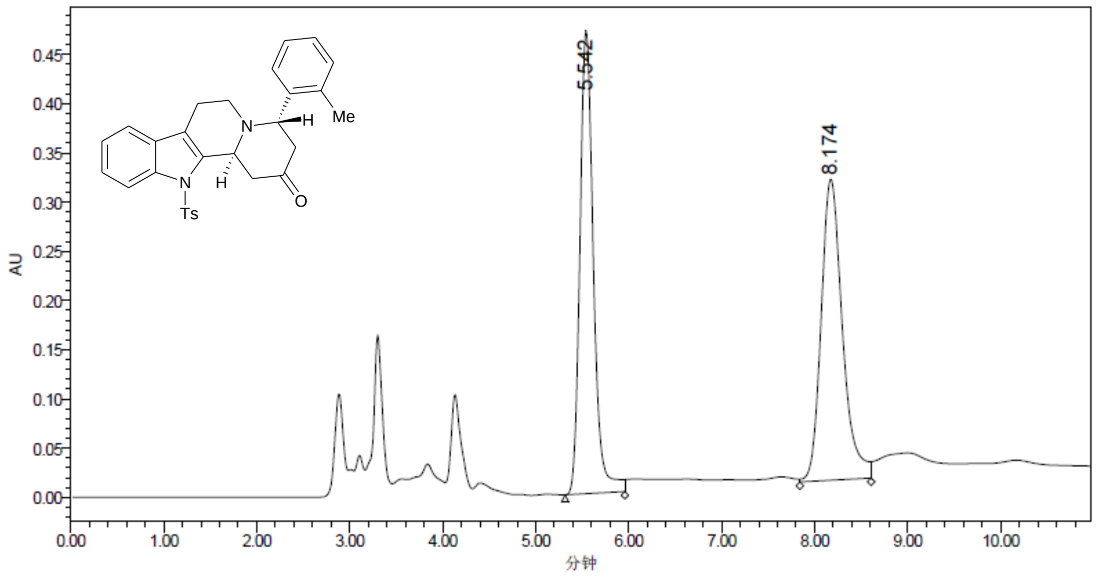

	RT (min)	Area (磺*sec)	% Area	Height (磺)	% Height
1	5.542	4737483	49.34	470092	60.57
2	8.174	4864579	50.66	305963	39.43

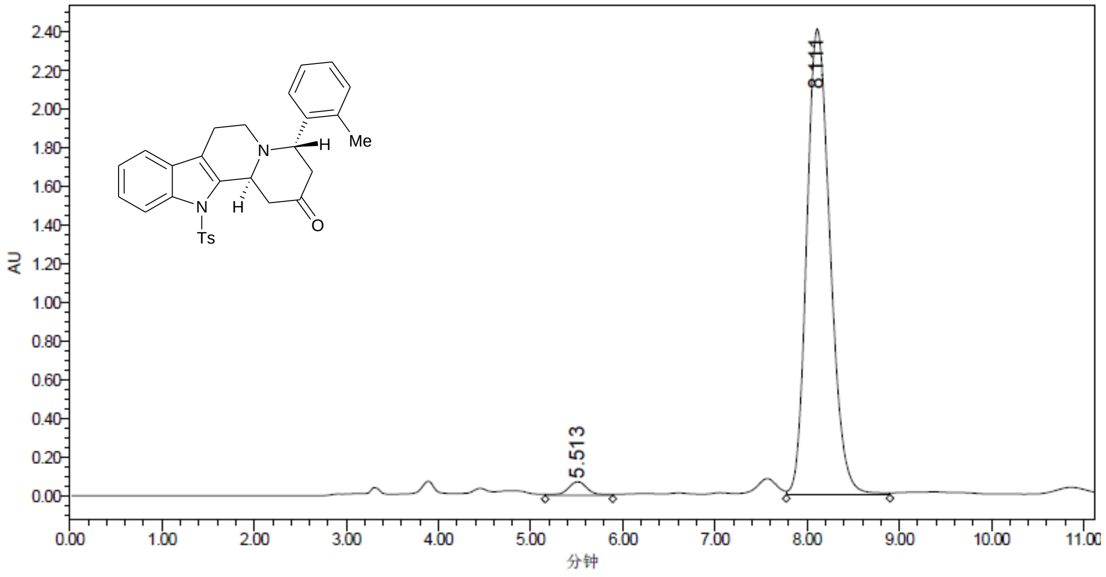

	RT (min)	Area (磺*sec)	% Area	Height (磺)	% Height
1	5.513	1035586	2.38	69720	2.81
2	8.111	42416839	97.62	2408481	97.19

(4R,12bS)-4-m-tolyl-12-tosyl-1,3,4,6,7,12b-hexahydroindolo[2,3-a]quinolizin-2(12 H)-one (6ad)

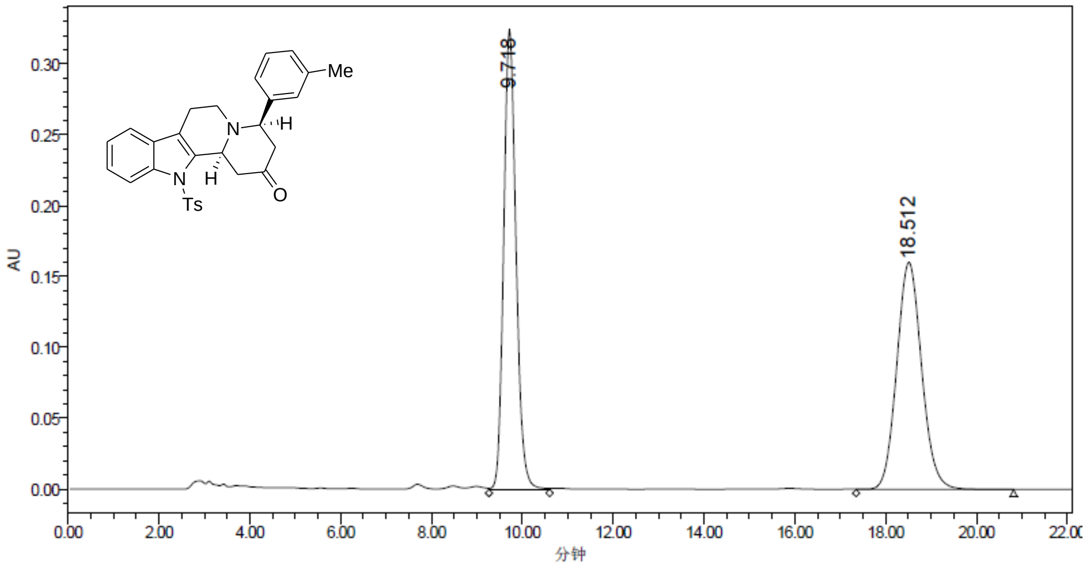

	RT (min)	Area (礦*sec)	% Area	Height (礦)	% Height
1	9.718	6108548	49.96	324377	66.92
2	18.512	6117766	50.04	160340	33.08

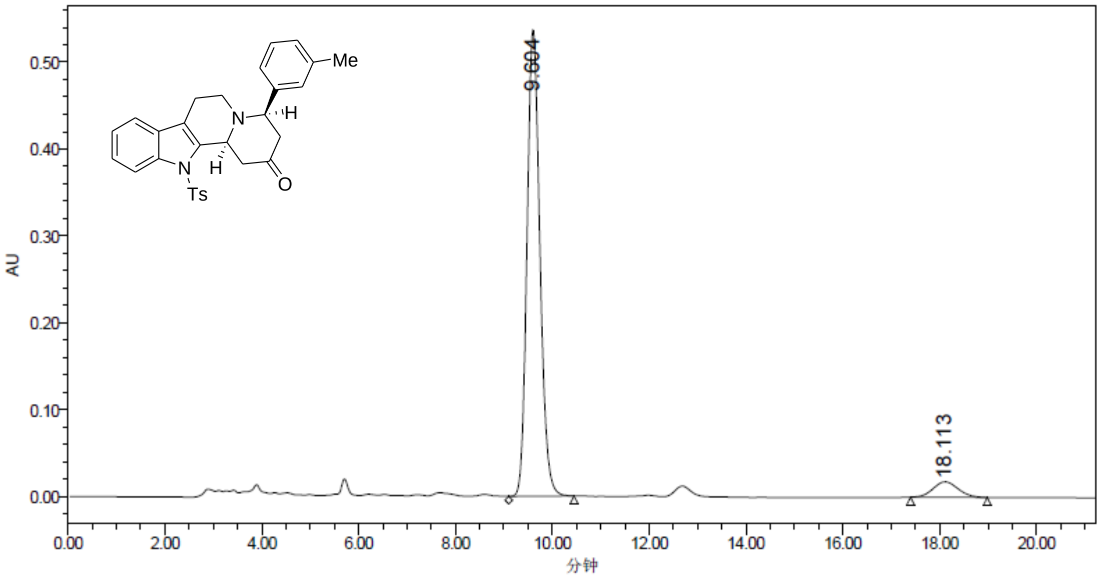

	RT (min)	Area (礦*sec)	% Area	Height (礦)	% Height
1	9.604	9880938	93.94	537006	96.79
2	18.113	637430	6.06	17788	3.21

(4S,12bS)-4-m-tolyl-12-tosyl-1,3,4,6,7,12b-hexahydroindolo[2,3-a]quinolizin-2(12 H)-one (7ad)

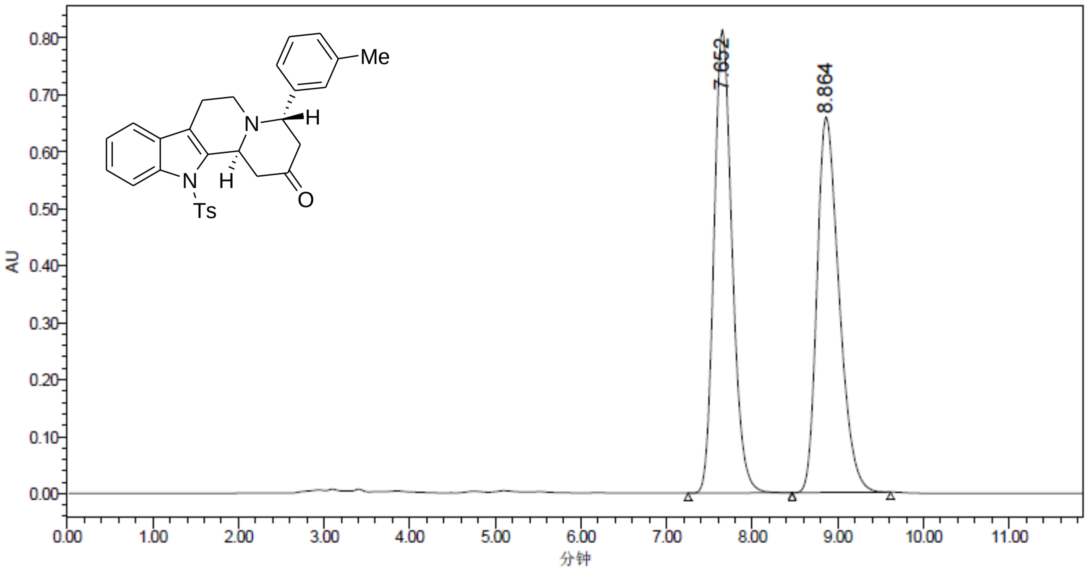

	RT (min)	Area (礦*sec)	% Area	Height (礦)	% Height
1	7.652	11912822	50.20	813573	55.26
2	8.864	11817736	49.80	658771	44.74

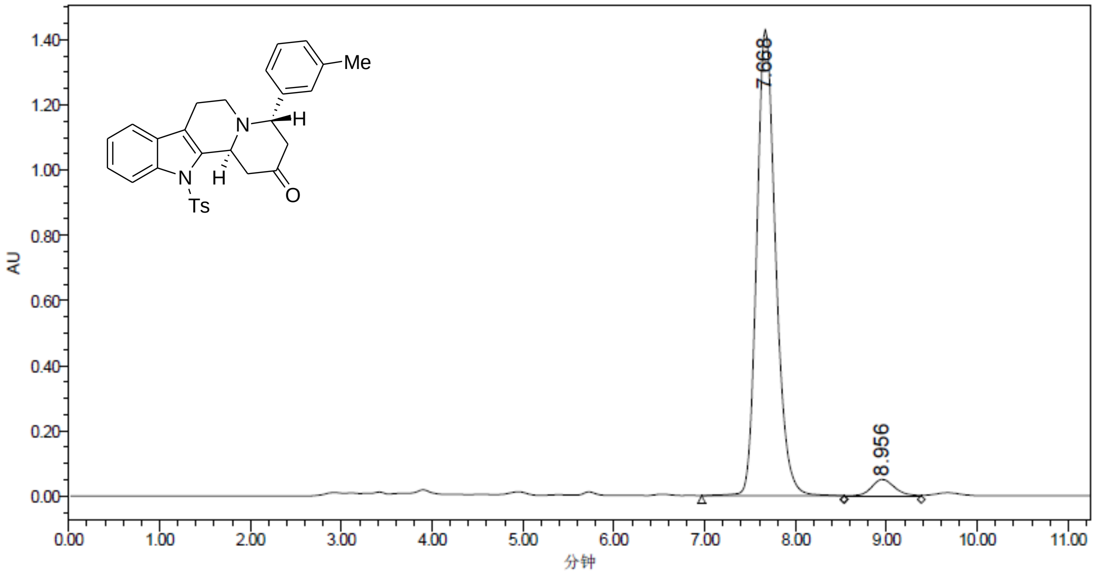

	RT (min)	Area (礦*sec)	% Area	Height (礦)	% Height
1	7.668	20620022	96.04	1429444	96.63
2	8.956	849450	3.96	49847	3.37

(4R,12bS)-4-p-tolyl-12-tosyl-1,3,4,6,7,12b-hexahydroindolo[2,3-a]quinolizin-2(12H)-one (6ae)

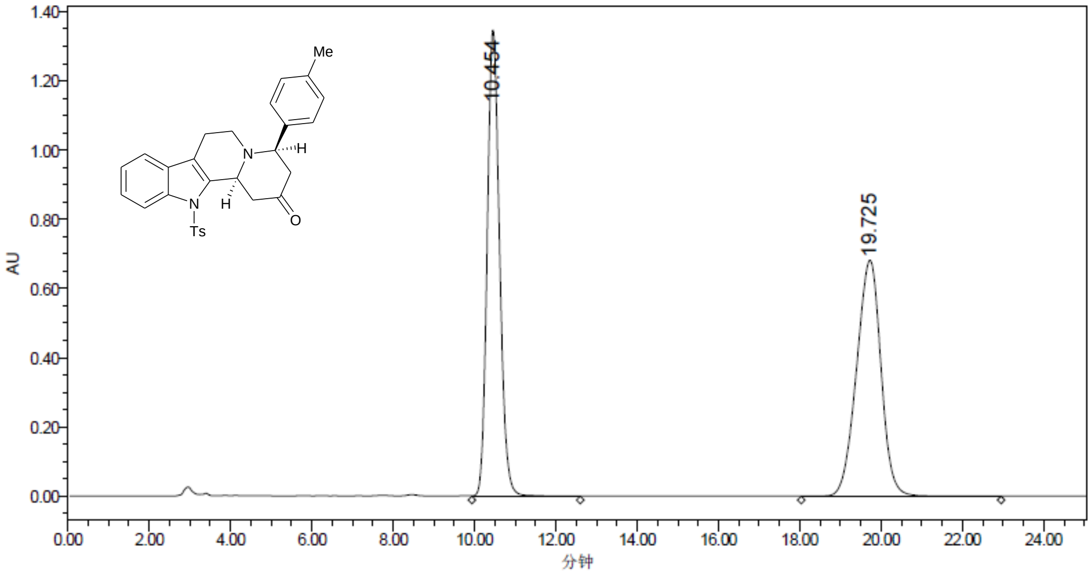

	RT (min)	Area (礦*sec)	% Area	Height (礦)	% Height
1	10.454	28458999	49.99	1347378	66.40
2	19.725	28465245	50.01	681956	33.60

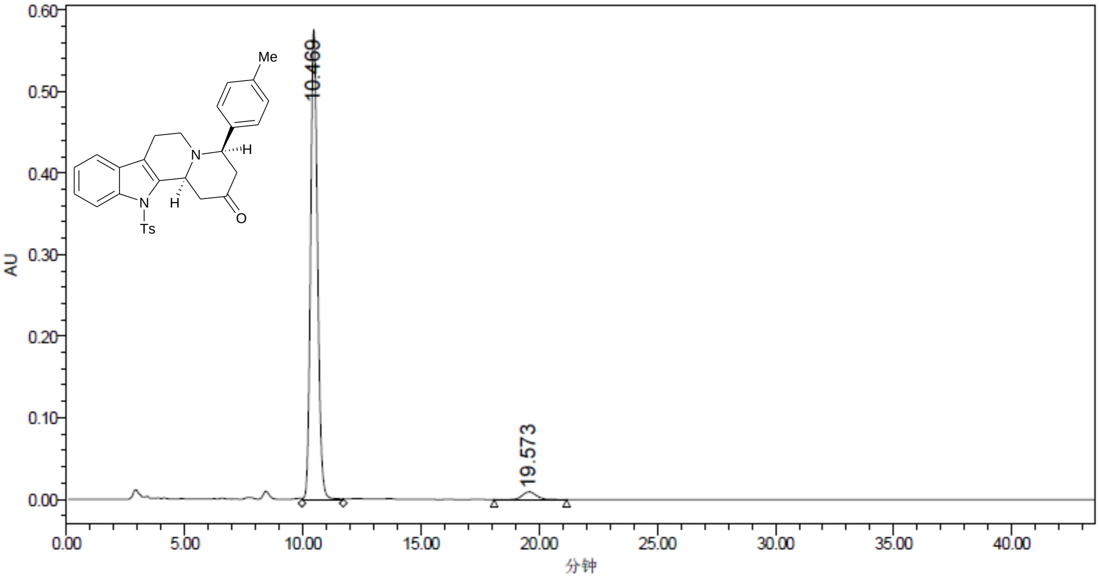

	RT (min)	Area (礦*sec)	% Area	Height (礦)	% Height
1	10.469	11839119	96.87	576254	98.40
2	19.573	383035	3.13	9345	1.60

(4S,12bS)-4-p-tolyl-12-tosyl-1,3,4,6,7,12b-hexahydroindolo[2,3-a]quinolizin-2(12H)-one (7ae)

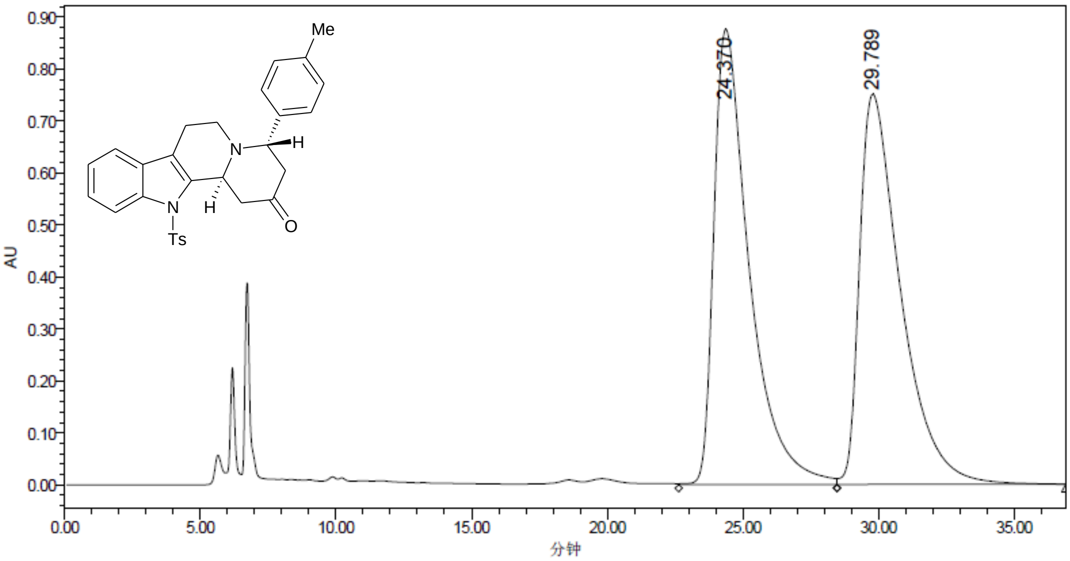

	RT (min)	Area (礦*sec)	% Area	Height (礦)	% Height
1	24.370	79401256	50.18	876103	53.86
2	29.789	78837453	49.82	750455	46.14

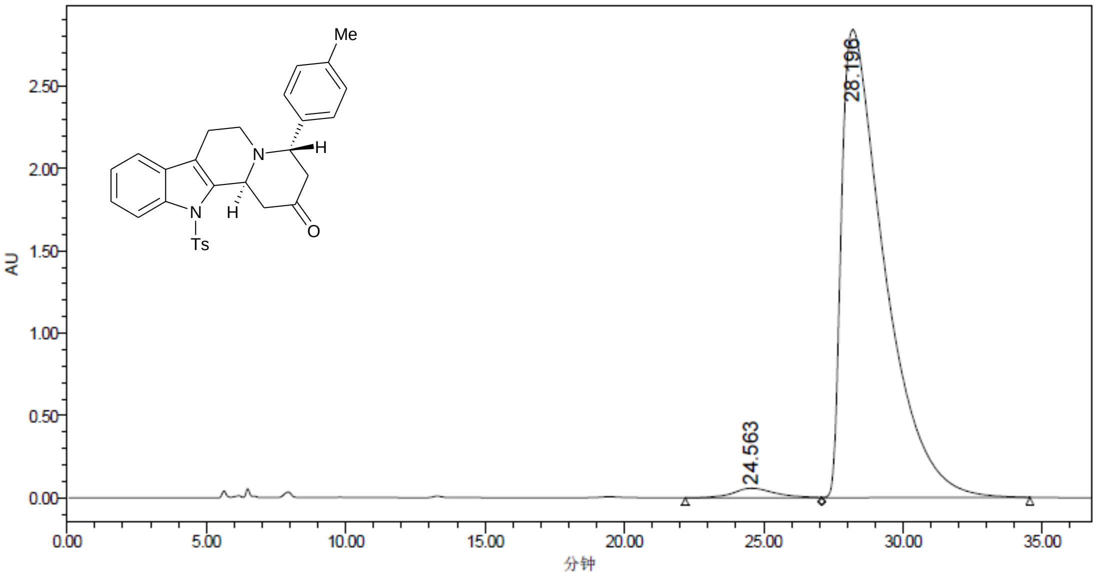

	RT (min)	Area (礦*sec)	% Area	Height (礦)	% Height
1	24.563	6137584	1.97	58533	2.02
2	28.196	304820813	98.03	2841250	97.98

(4R,12bS)-4-(4-chlorophenyl)-12-tosyl-1,3,4,6,7,12b-hexahydroindolo[2,3-a]quinolizin-2(12H)-one (6af)

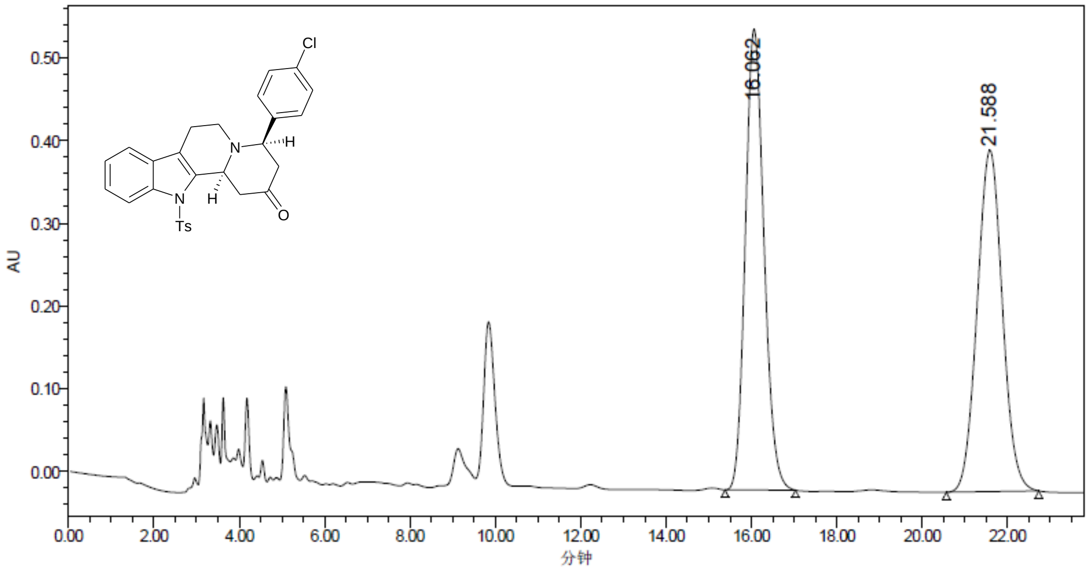

	RT (min)	Area (磺*sec)	% Area	Height (磺)	% Height
1	16.062	16932795	50.01	557035	57.43
2	21.588	16925162	49.99	412826	42.57

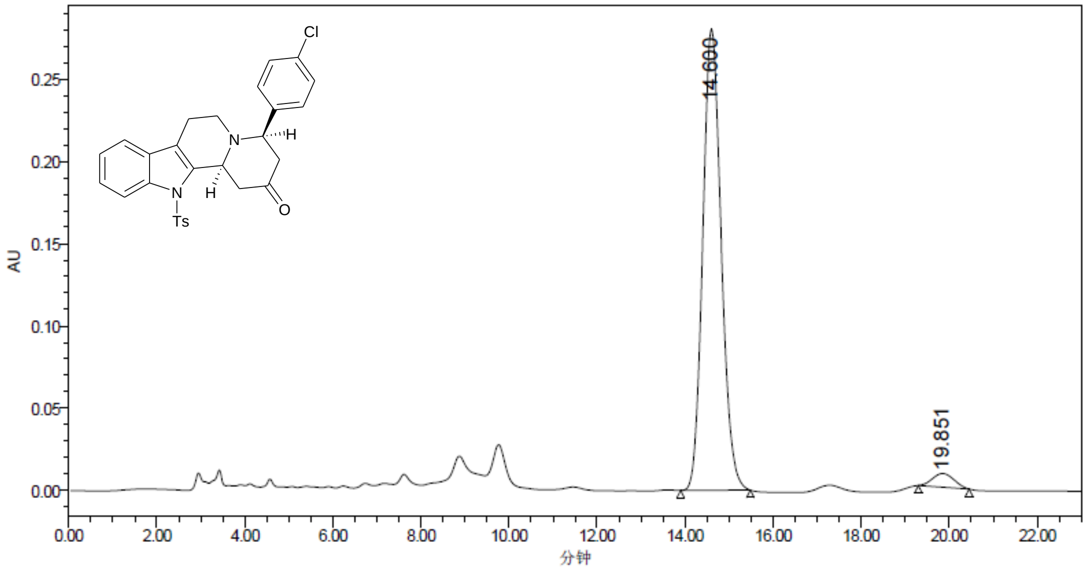

	RT (min)	Area (磺*sec)	% Area	Height (磺)	% Height
1	14.600	8140705	96.65	280112	97.12
2	19.851	282139	3.35	8304	2.88

(4S,12bS)-4-(4-chlorophenyl)-12-tosyl-1,3,4,6,7,12b-hexahydroindolo[2,3-a]quinolizin-2(12H)-one (7af)

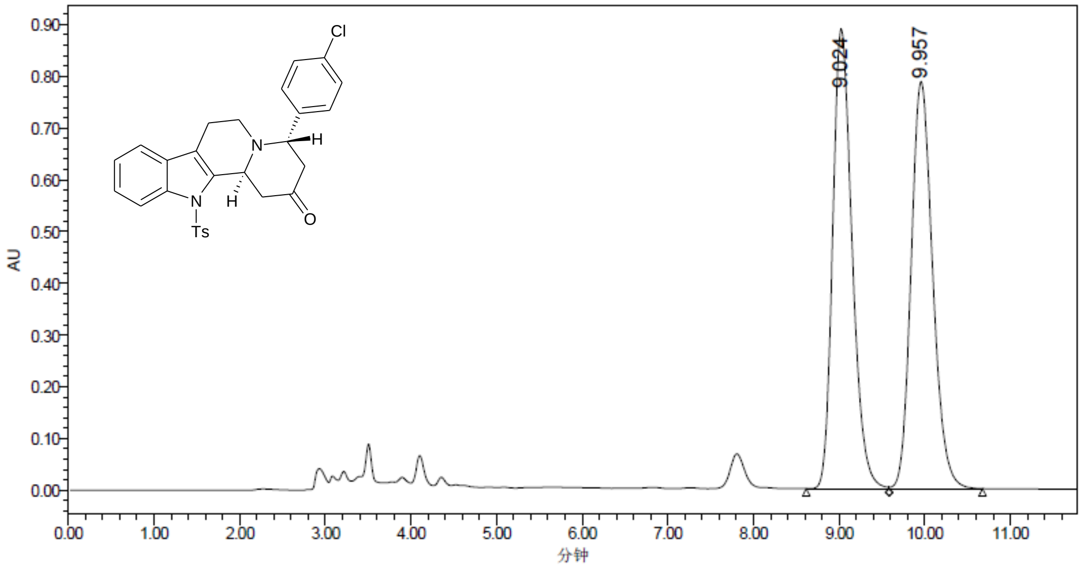

	RT (min)	Area (礦*sec)	% Area	Height (礦)	% Height
1	9.024	13971050	50.36	887905	53.01
2	9.957	13770748	49.64	786940	46.99

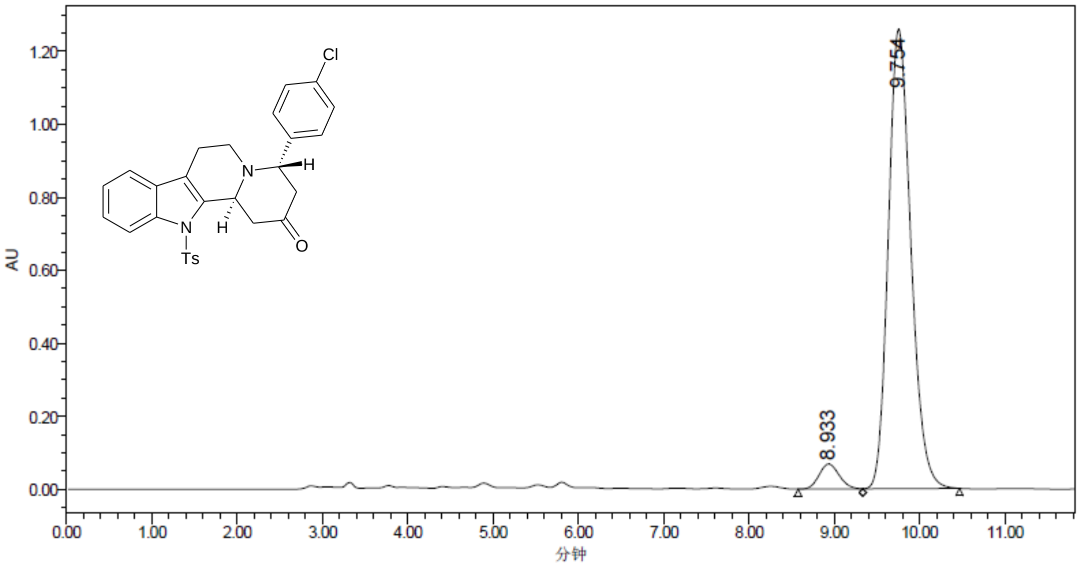

	RT (min)	Area (礦*sec)	% Area	Height (礦)	% Height
1	8.933	1117930	4.61	68283	5.15
2	9.754	23125555	95.39	1258557	94.85

(4R,12bS)-4-(4-bromophenyl)-12-tosyl-1,3,4,6,7,12b-hexahydroindolo[2,3-a]quinolizin-2(12H)-one (6ag)

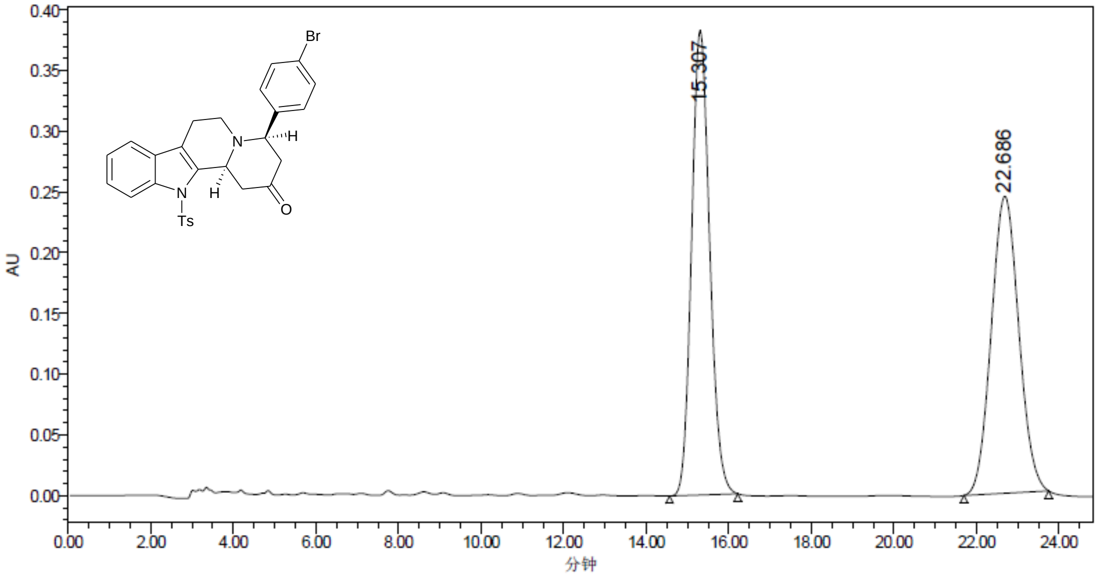

	RT (min)	Area (礦*sec)	% Area	Height (礦)	% Height
1	15.307	11636180	50.66	382761	61.00
2	22.686	11333437	49.34	244743	39.00

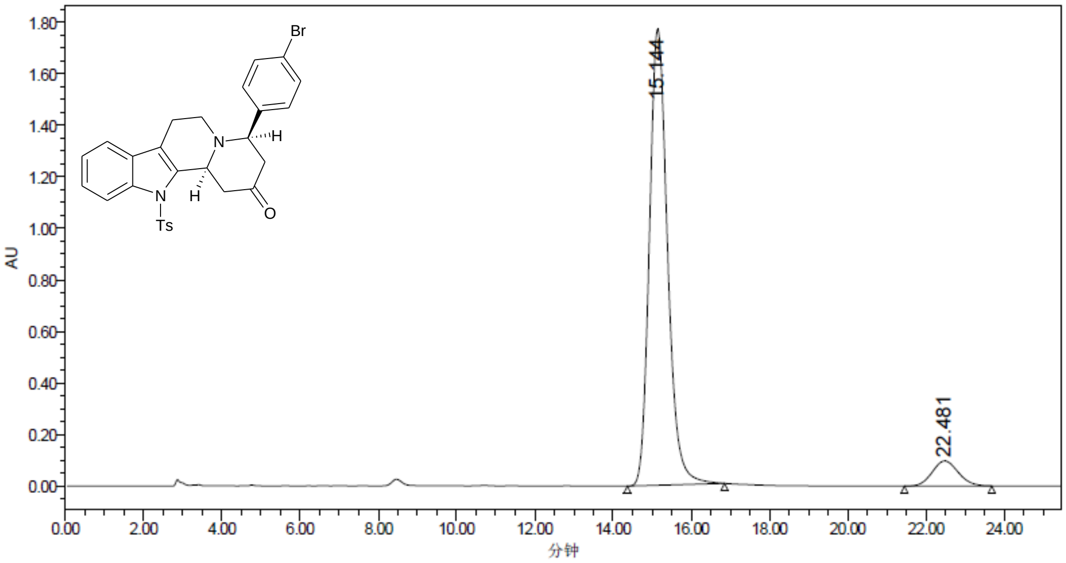

	RT (min)	Area (礦*sec)	% Area	Height (礦)	% Height
1	15.144	56963946	92.66	1770535	94.79
2	22.481	4513051	7.34	97402	5.21

(4S,12bS)-4-(4-bromophenyl)-12-tosyl-1,3,4,6,7,12b-hexahydroindolo[2,3-a]quinolizin-2(12H)-one (7ag)

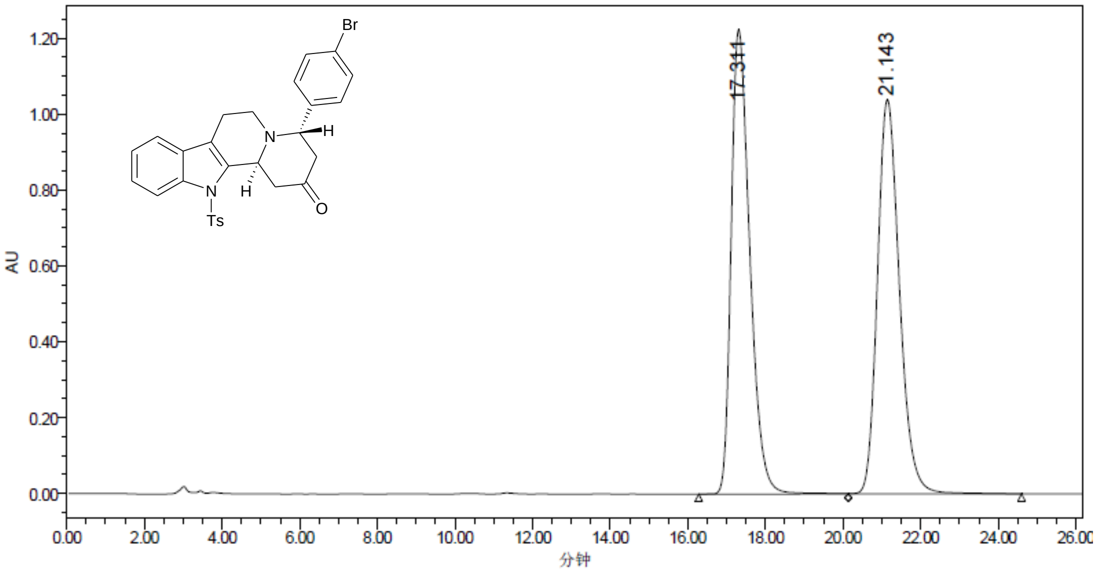

	RT (min)	Area (礦*sec)	% Area	Height (礦)	% Height
1	17.311	40808456	49.90	1224676	54.09
2	21.143	40972003	50.10	1039319	45.91

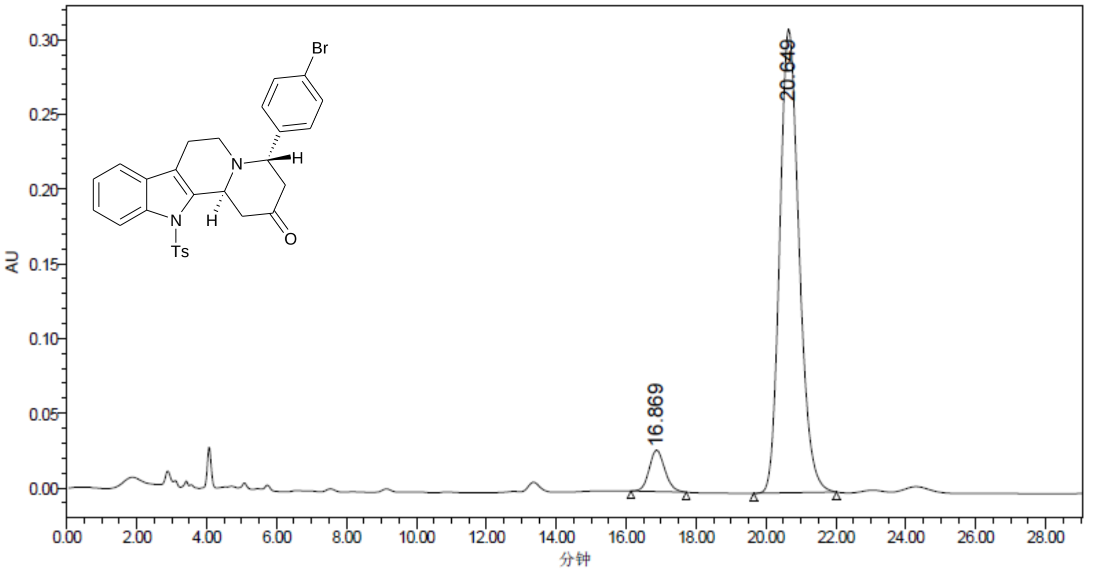

	RT (min)	Area (礦*sec)	% Area	Height (礦)	% Height
1	16.869	860801	6.72	27624	8.18
2	20.649	11949098	93.28	309944	91.82

(4R,12bS)-12-tosyl-4-(4-(trifluoromethyl)phenyl)-1,3,4,6,7,12b-hexahydroindolo[2,3-a]quinolizin-2(12H)-one (6ah)

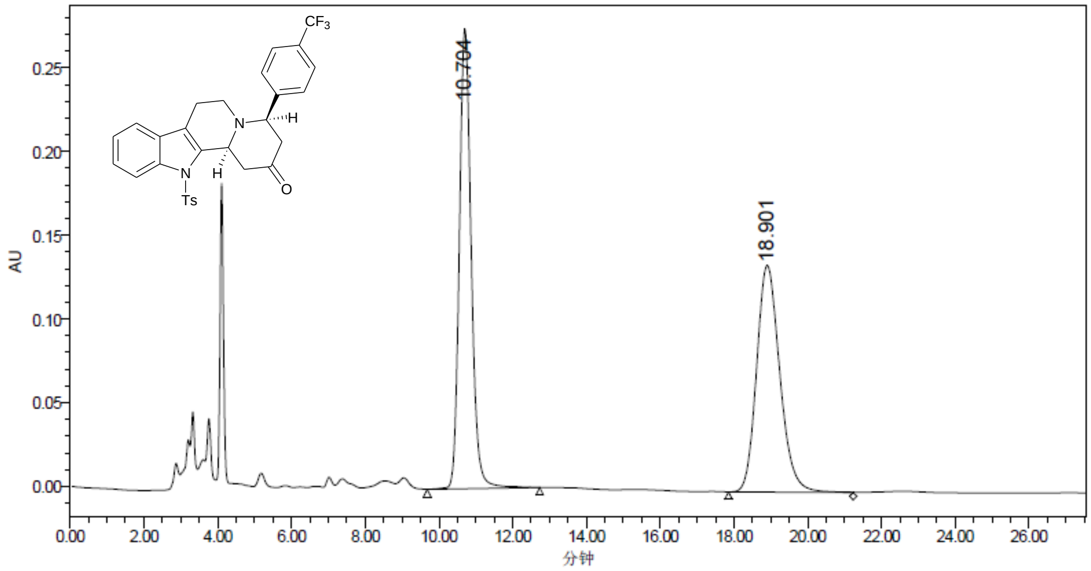

	RT (min)	Area (磺*sec)	% Area	Height (磺)	% Height
1	10.704	6143607	50.94	274565	66.97
2	18.901	5916034	49.06	135395	33.03

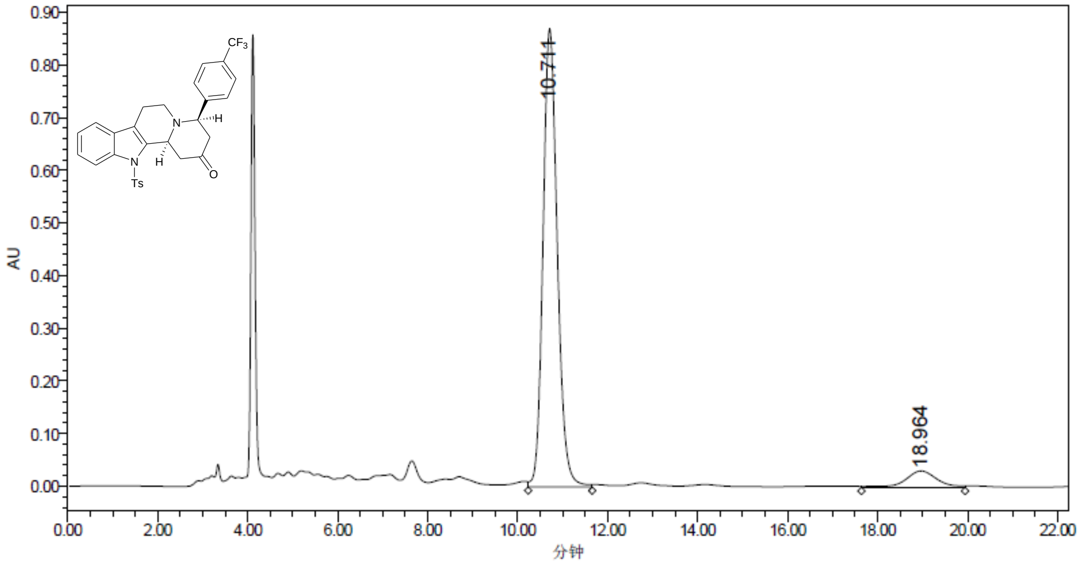

	RT (min)	Area (磺*sec)	% Area	Height (磺)	% Height
1	10.711	19144272	92.78	869692	96.56
2	18.964	1490615	7.22	31020	3.44

(4S,12bS)-12-tosyl-4-(4-(trifluoromethyl)phenyl)-1,3,4,6,7,12b-hexahydroindolo[2,3-a]quinolizin-2(12H)-one (7ah)

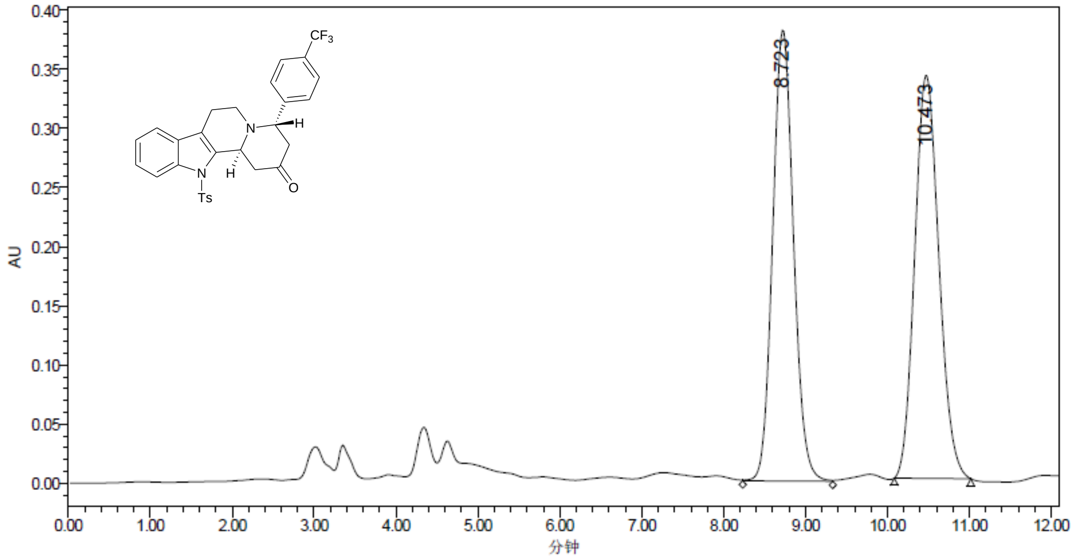

	RT (min)	Area (磺*sec)	% Area	Height (磺)	% Height
1	8.723	6732925	49.08	381466	52.81
2	10.473	6984577	50.92	340901	47.19

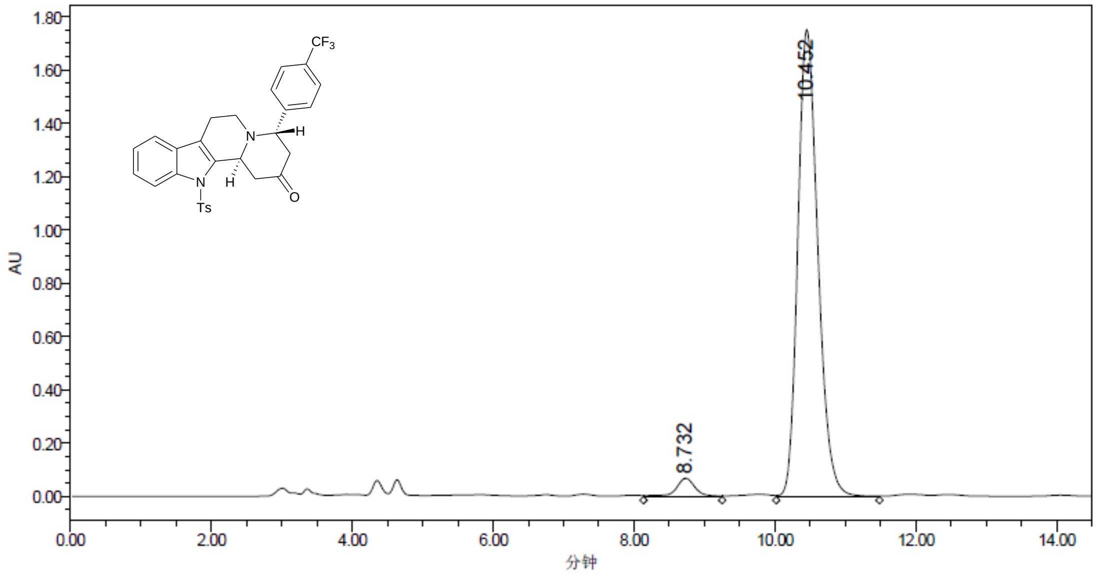

	RT (min)	Area (磺*sec)	% Area	Height (磺)	% Height
1	8.732	1276671	3.56	68463	3.76
2	10.452	34628979	96.44	1752243	96.24

(4R,12bS)-4-(3-nitrophenyl)-12-tosyl-1,3,4,6,7,12b-hexahydroindolo[2,3-a]quinolizin-2(12H)-one (6ai)

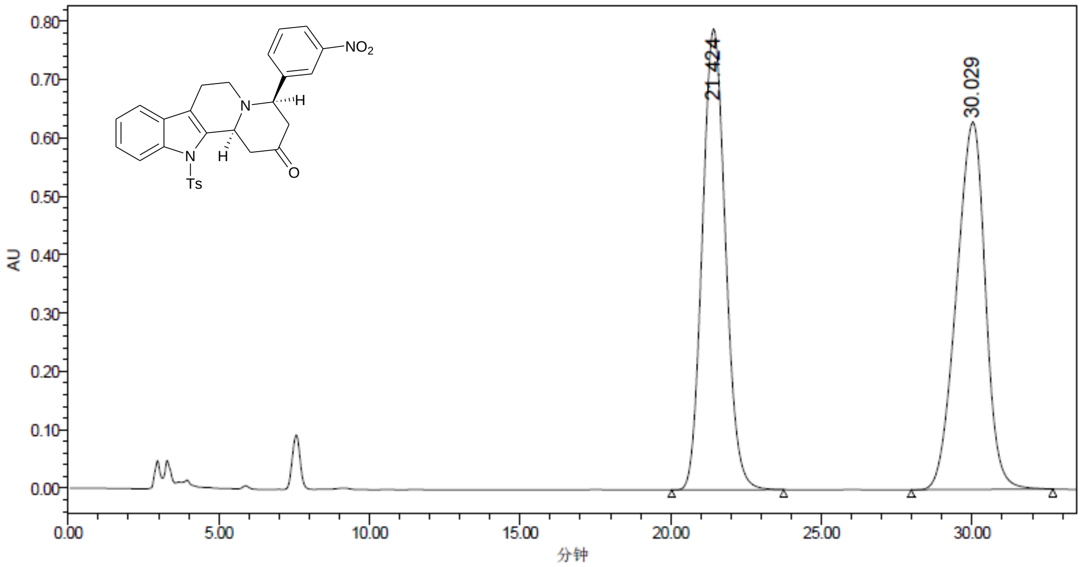

	RT (min)	Area (礦*sec)	% Area	Height (礦)	% Height
1	21.424	42004164	50.00	789309	55.65
2	30.029	42010200	50.00	629132	44.35

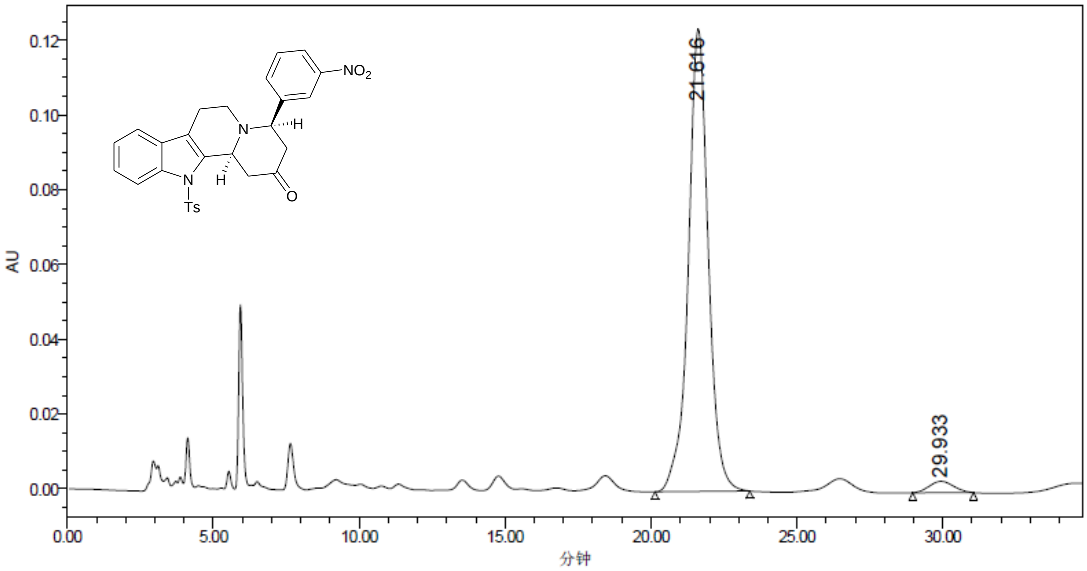

	RT (min)	Area (礦*sec)	% Area	Height (礦)	% Height
1	21.616	5876348	97.10	123642	97.60
2	29.933	175397	2.90	3037	2.40

(4S,12bS)-4-(3-nitrophenyl)-12-tosyl-1,3,4,6,7,12b-hexahydroindolo[2,3-a]quinolizin-2(12H)-one (7ai)

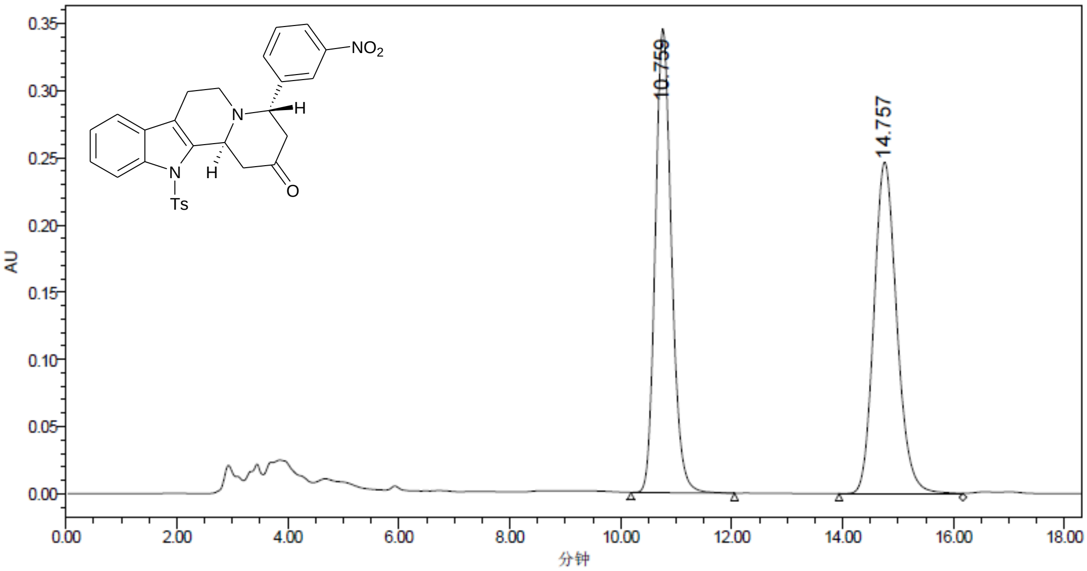

	RT (min)	Area (礦*sec)	% Area	Height (礦)	% Height
1	10.759	7032030	49.91	345501	58.32
2	14.757	7058709	50.09	246936	41.68

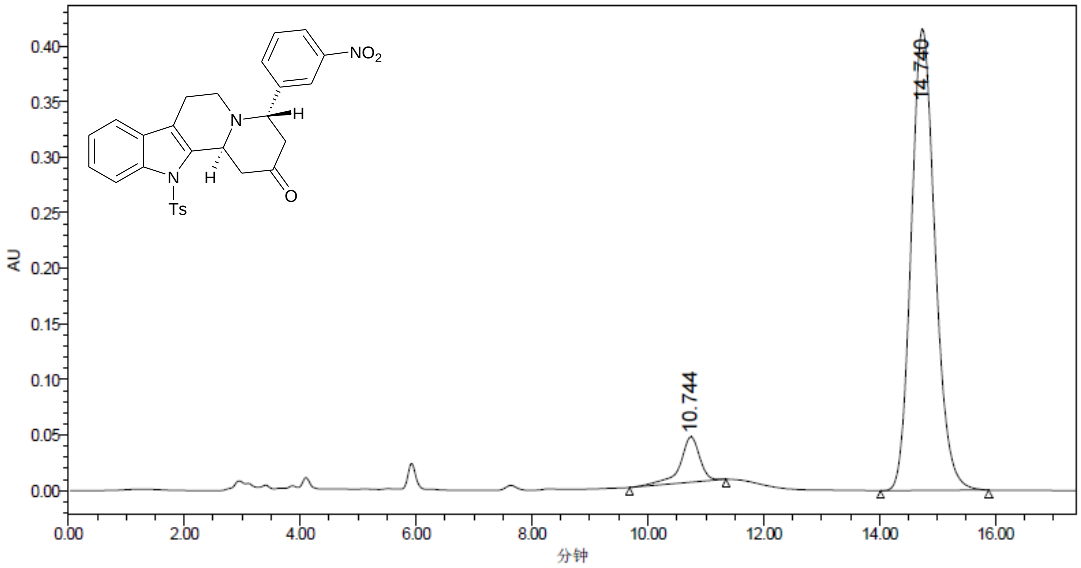

	RT (min)	Area (礦*sec)	% Area	Height (礦)	% Height
1	10.744	960581	7.50	40724	8.93
2	14.740	11849259	92.50	415281	91.07

(4R,12bS)-4-(perfluorophenyl)-12-tosyl-1,3,4,6,7,12b-hexahydroindolo[2,3-a]quinolizin-2(12H)-one (6aj)

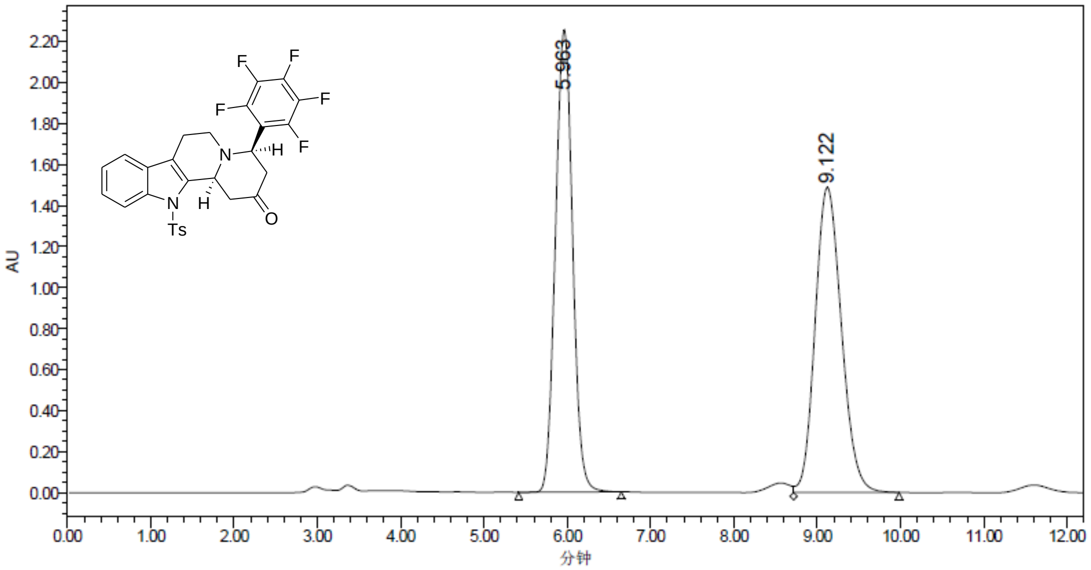

	RT (min)	Area (礦*sec)	% Area	Height (礦)	% Height
1	5.963	32239971	49.72	2256084	60.25
2	9.122	32596630	50.28	1488455	39.75

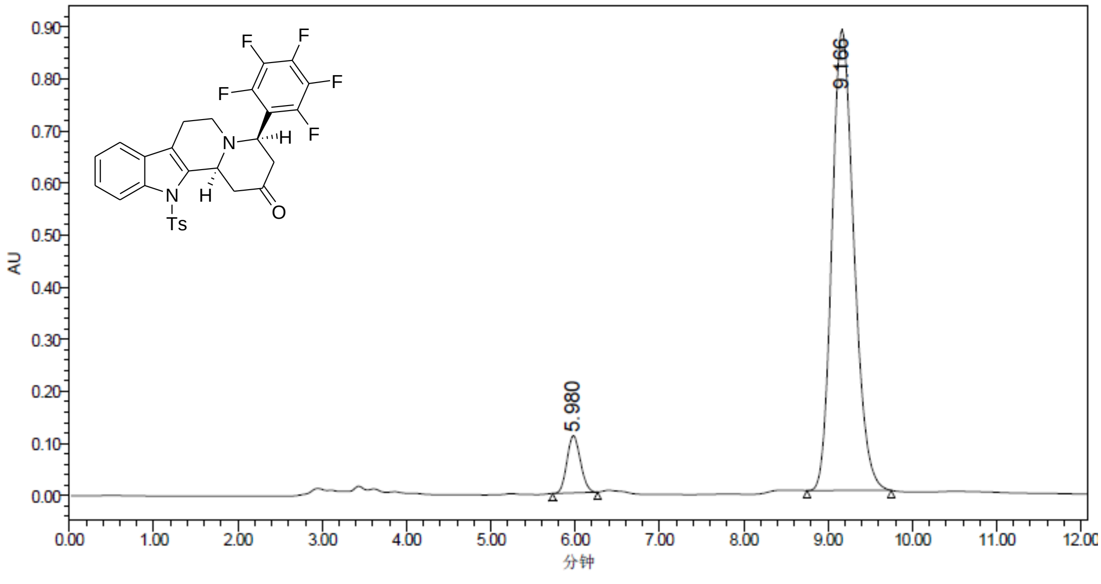

	RT (min)	Area (礦*sec)	% Area	Height (礦)	% Height
1	5.980	1220550	7.08	109814	11.05
2	9.166	16021575	92.92	884218	88.95

(4R,12bS)-4-(2,3-dichlorophenyl)-12-tosyl-1,3,4,6,7,12b-hexahydroindolo[2,3-a]quinolizin-2(12H)-one (6ak)

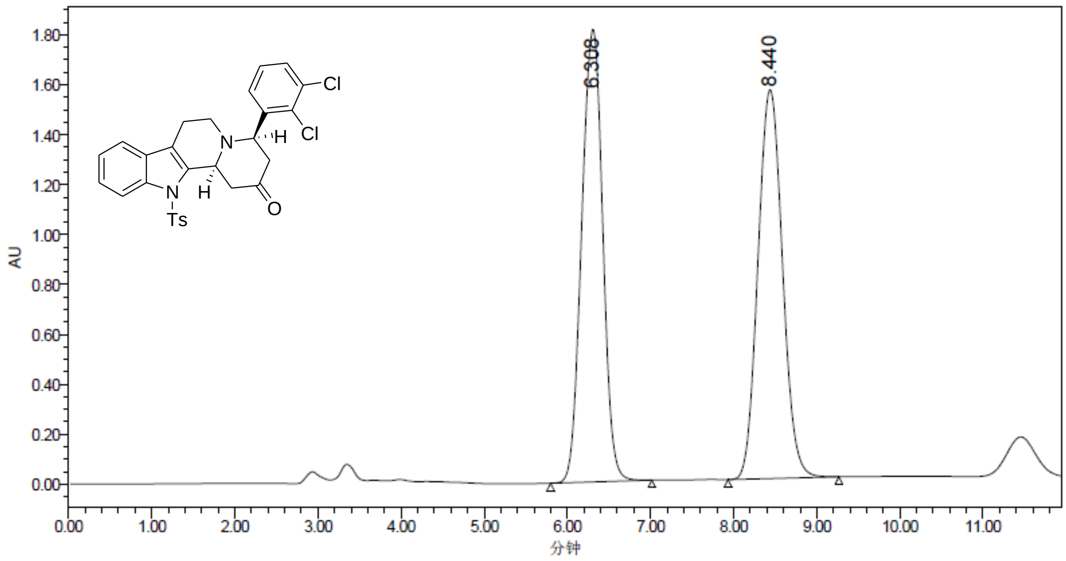

	RT (min)	Area (礦*sec)	% Area	Height (礦)	% Height
1	6.308	31820119	49.95	1814144	53.78
2	8.440	31878757	50.05	1558899	46.22

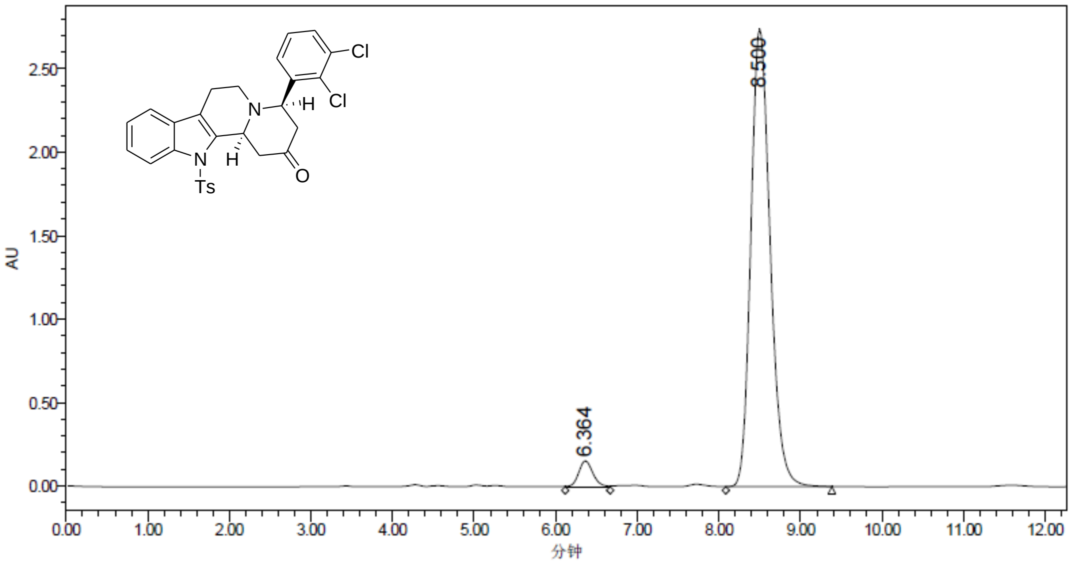

	RT (min)	Area (礦*sec)	% Area	Height (礦)	% Height
1	6.364	1851596	3.95	154739	5.35
2	8.500	44972089	96.05	2740268	94.65

(4R,12bS)-4-(2,4-dinitrophenyl)-12-tosyl-1,3,4,6,7,12b-hexahydroindolo[2,3-a]quinolizin-2(12H)-one (6al)

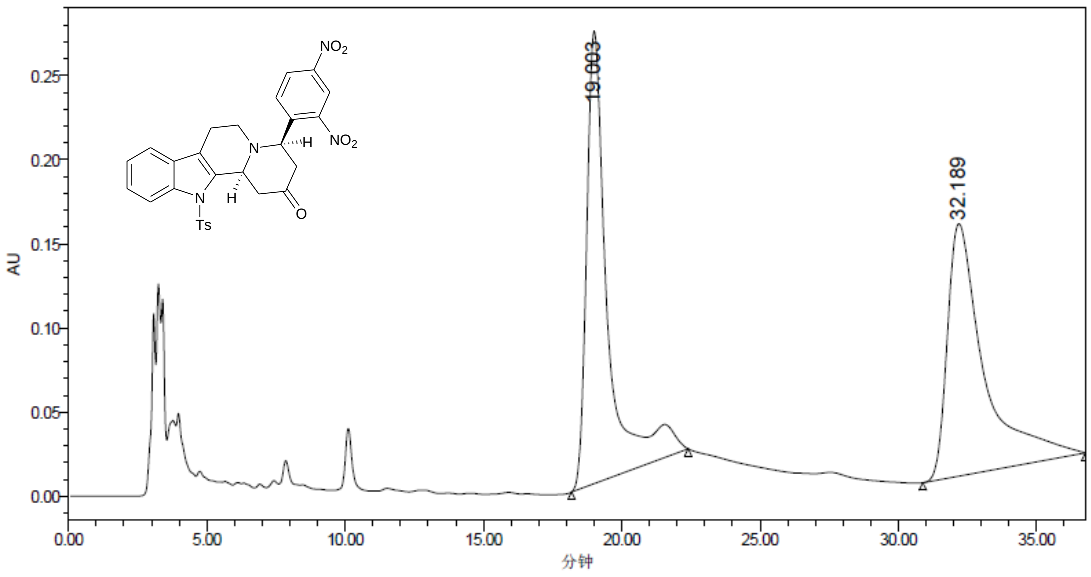

	RT (min)	Area (磺*sec)	% Area	Height (磺)	% Height
1	19.003	14250485	50.62	268794	64.18
2	32.189	13898744	49.38	150047	35.82

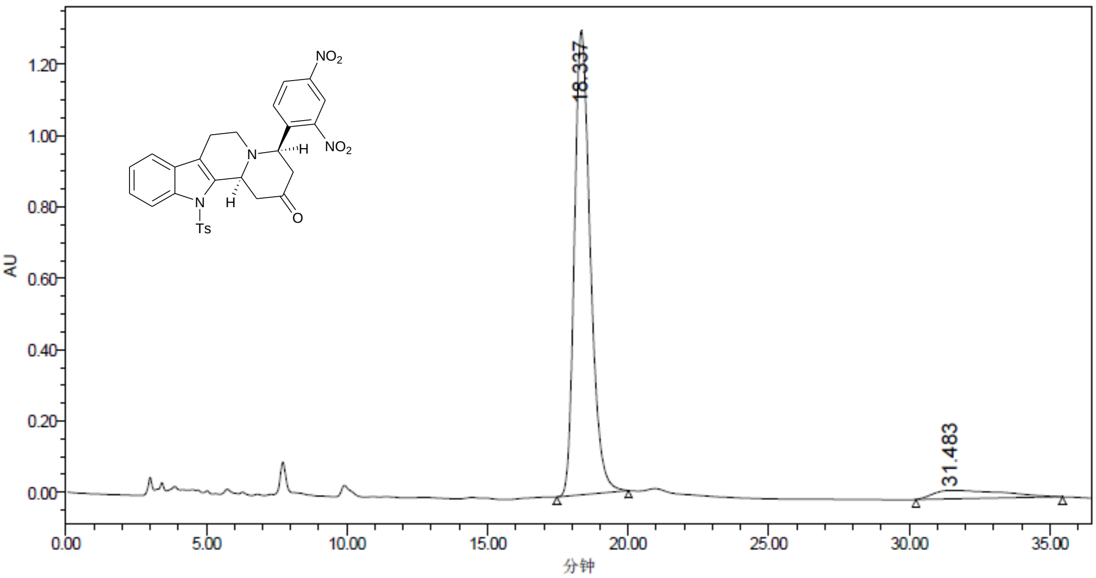

	RT (min)	Area (磺*sec)	% Area	Height (磺)	% Height
1	18.337	51267062	92.68	1301561	98.19
2	31.483	4051579	7.32	23930	1.81

(4R,12bS)-4-(thiophen-2-yl)-12-tosyl-1,3,4,6,7,12b-hexahydroindolo[2,3-a]quinolizin-2(12H)-one (6am)

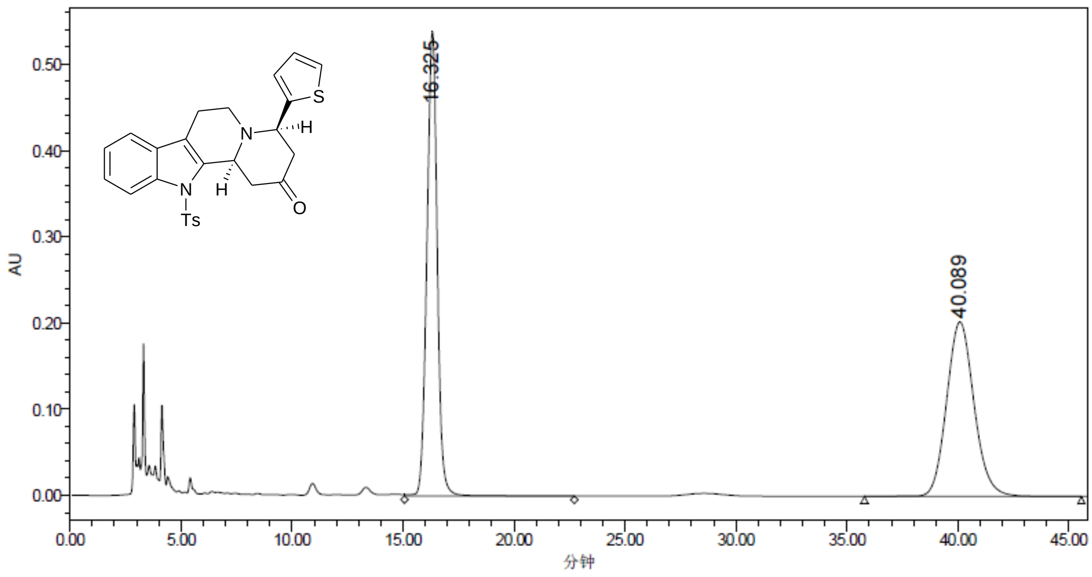

	RT (min)	Area (礦*sec)	% Area	Height (礦)	% Height
1	16.325	17463526	50.08	538494	72.68
2	40.089	17409898	49.92	202463	27.32

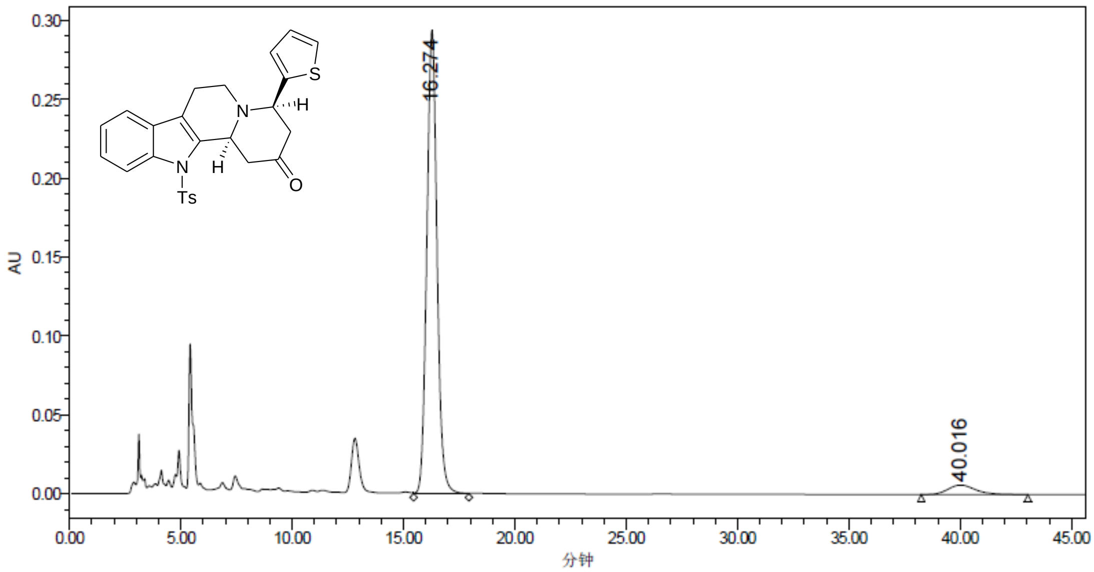

	RT (min)	Area (礦*sec)	% Area	Height (礦)	% Height
1	16.274	9368534	94.65	293546	97.98
2	40.016	529384	5.35	6059	2.02

(4S,12bS)-4-(thiophen-2-yl)-12-tosyl-1,3,4,6,7,12b-hexahydroindolo[2,3-a]quinolizin-2(12H)-one (7am)

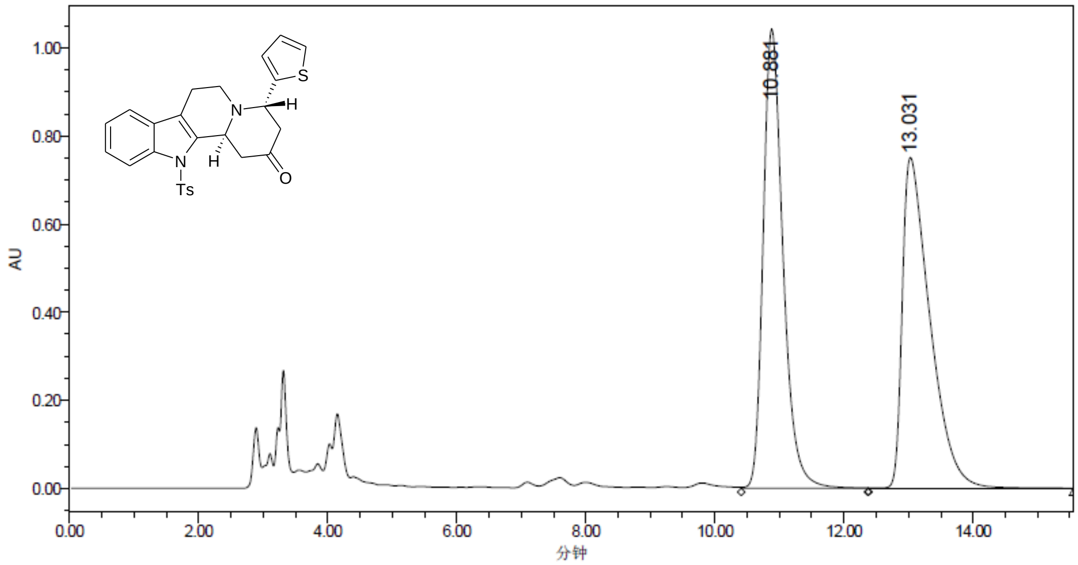

	RT (min)	Area (礦*sec)	% Area	Height (礦)	% Height
1	10.881	22255907	49.98	1042172	58.12
2	13.031	22270348	50.02	750883	41.88

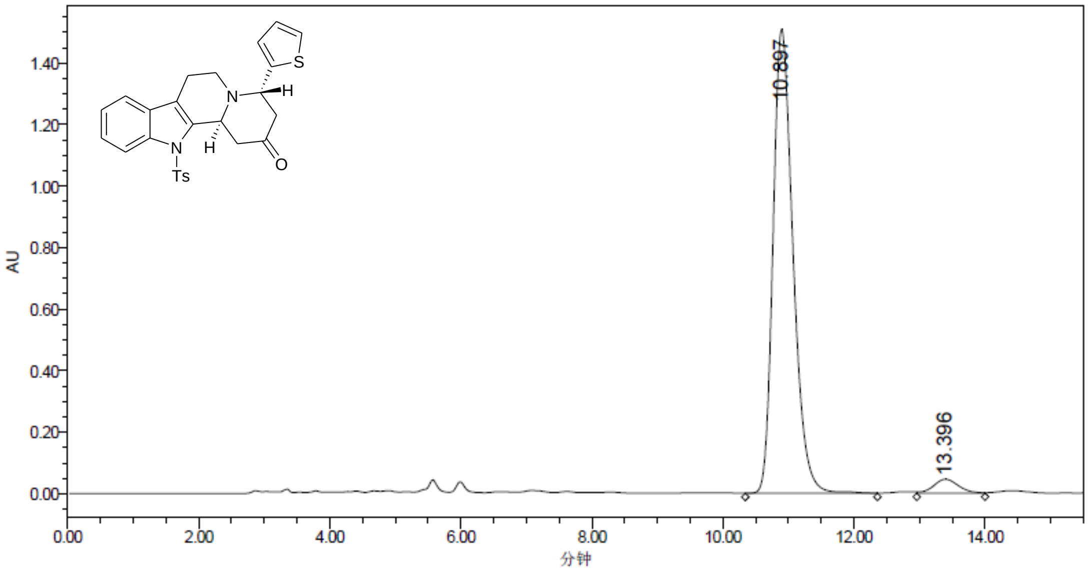

	RT (min)	Area (礦*sec)	% Area	Height (礦)	% Height
1	10.897	31898421	96.43	1508190	97.09
2	13.396	1180110	3.57	45183	2.91

(4R,12bS)-4-(furan-2-yl)-12-tosyl-1,3,4,6,7,12b-hexahydroindolo[2,3-a]quinolizin-2(12H)-one (6an)

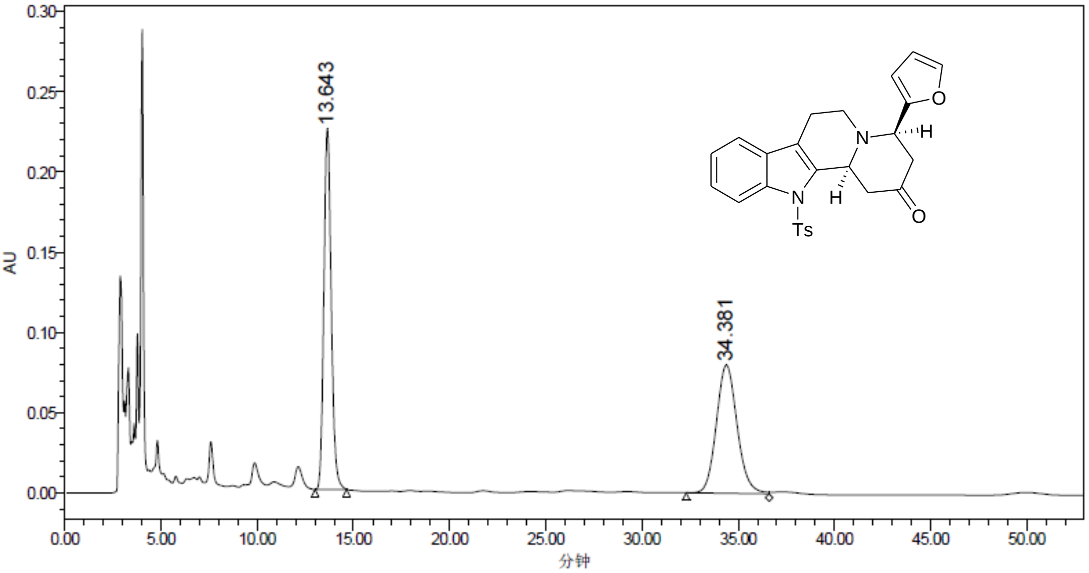

	RT (min)	Area (磺*sec)	% Area	Height (磺)	% Height
1	13.643	5978172	49.88	224792	73.78
2	34.381	6007709	50.12	79890	26.22

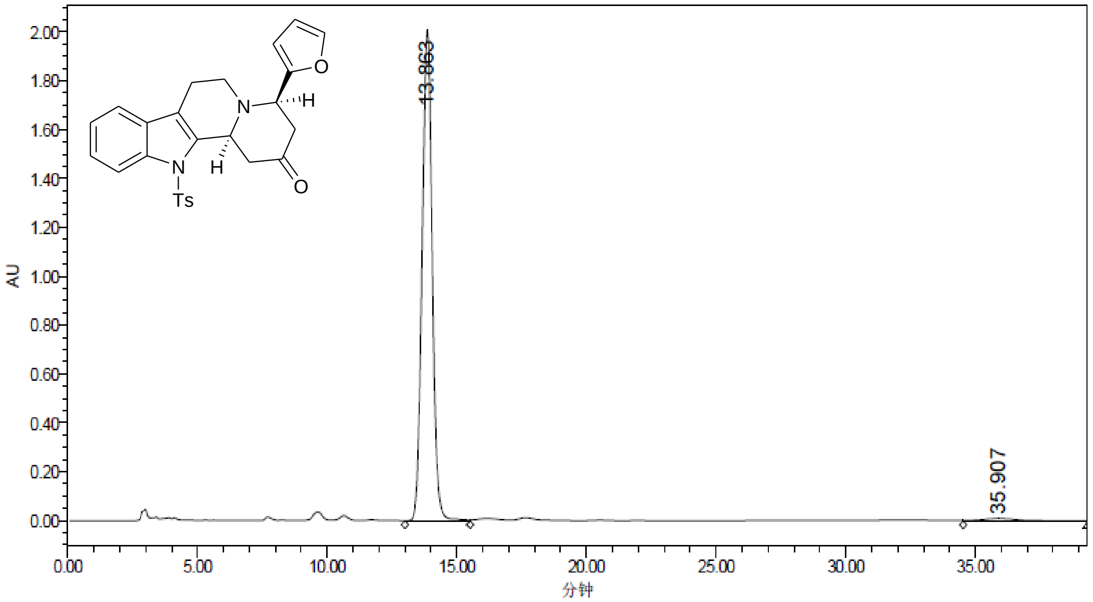

	RT (min)	Area (磺*sec)	% Area	Height (磺)	% Height
1	13.863	54302094	98.16	2007336	99.51
2	35.907	1015123	1.84	9952	0.49

(4S,12bS)-4-(furan-2-yl)-12-tosyl-1,3,4,6,7,12b-hexahydroindolo[2,3-a]quinolizin-2(12H)-one (7an)

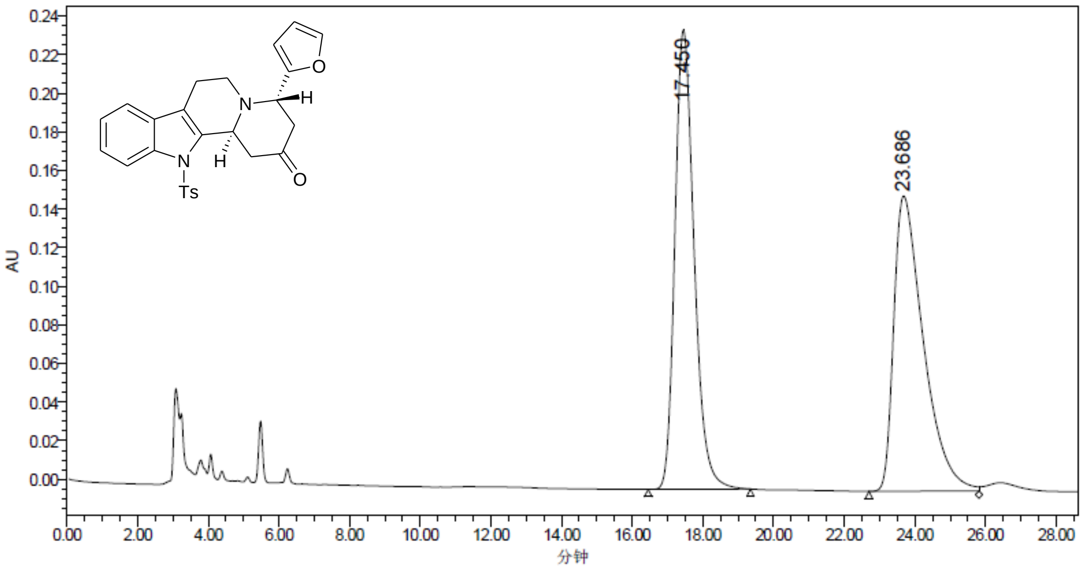

	RT (min)	Area (礦*sec)	% Area	Height (礦)	% Height
1	17.450	8971460	50.20	237939	60.87
2	23.686	8901728	49.80	152949	39.13

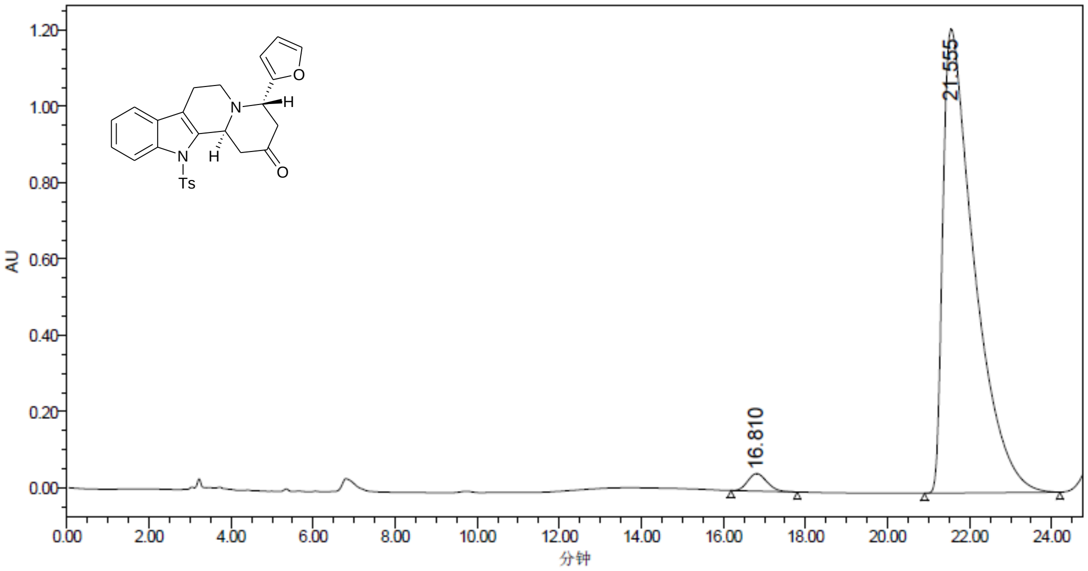

	RT (min)	Area (礦*sec)	% Area	Height (礦)	% Height
1	16.810	1492725	2.26	45821	3.63
2	21.555	64463512	97.74	1216228	96.37

(4R,12bS)-4-(2-methylprop-1-enyl)-12-tosyl-1,3,4,6,7,12b-hexahydroindolo[2,3-a] quinolizin-2(12H)-one (6ao)

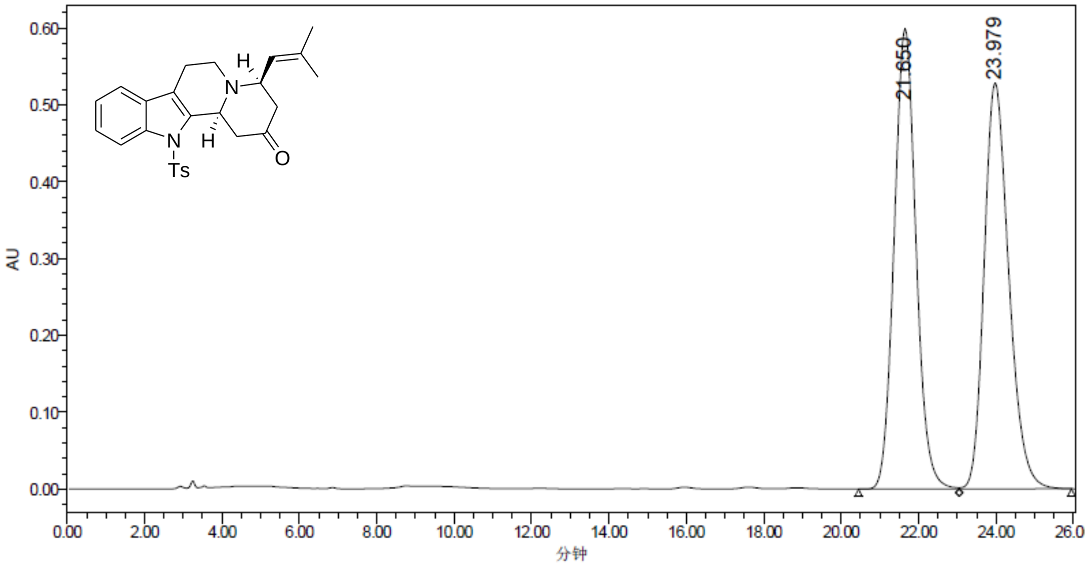

	RT (min)	Area (礦*sec)	% Area	Height (礦)	% Height
1	21.650	23468318	50.07	598416	53.14
2	23.979	23398599	49.93	527754	46.86

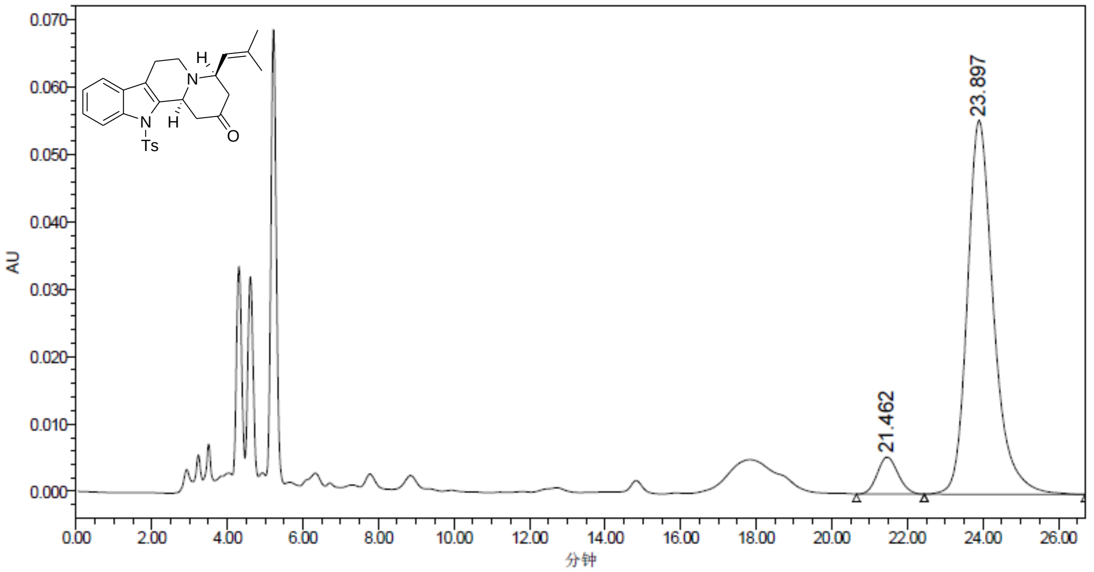

	RT (min)	Area (礦*sec)	% Area	Height (礦)	% Height
1	21.462	210616	7.28	5492	9.01
2	23.897	2681560	92.72	55463	90.99
